# ﻿Revision of the endemic African genus *Dinapsis* (Dinapsini, Megalyridae, Hymenoptera) with description of seven new species

**DOI:** 10.3897/zookeys.1112.82307

**Published:** 2022-07-12

**Authors:** Simon van Noort, Scott Richard Shaw, Robert S. Copeland

**Affiliations:** 1 Research and Exhibitions Department, South African Museum, Iziko Museums of South Africa, PO Box 61, Cape Town 8000 South Africa South African Museum, Iziko Museums of South Africa Cape Town South Africa; 2 Department of Biological Sciences, University of Cape Town, Private Bag, Rondebosch, 7701, South Africa University of Cape Town Cape Town South Africa; 3 U.W. Insect Museum, Department of Ecosystem Science and Management (3354), University of Wyoming, 1000 East University Avenue, Laramie, Wyoming 82071-3354, USA University of Wyoming Laramie United States of America; 4 International Centre of Insect Physiology and Ecology (ICIPE), P.O. Box 30772 Nairobi, Kenya International Centre of Insect Physiology and Ecology (ICIPE) Nairobi Kenya; 5 Department of Entomology, National Museum of Natural History, Smithsonian Institution, Washington DC, USA National Museum of Natural History, Smithsonian Institution Washington United States of America

**Keywords:** Africa, barcode, holotype, identification key, new species, parasitoid, taxonomy, wasp

## Abstract

The endemic Afrotropical genus *Dinapsis* is revised, and seven new species are described and illustrated: *D.bicolor* van Noort & Shaw, **sp. nov.**, *D.gamka* van Noort & Shaw, **sp. nov.**, *D.igneus* van Noort & Shaw, **sp. nov.**, *D.spinitibia* van Noort & Shaw, **sp. nov.**, *D.taita* van Noort & Shaw, **sp. nov.**, *D.tricolor* Shaw & van Noort, **sp. nov.**, *D.zulu* Shaw & van Noort, **sp. nov.** The distribution of the Central African Republican species *D.centralis* Shaw & van Noort, 2009 is extended to include Cameroon, Kenya and Uganda. *Dinapsisturneri* Waterston, 1922, previously only known from the poorly preserved holotype female, is redescribed based on newly collected specimens. The distribution of this Western Cape species is extended to include the Eastern and Northern Cape provinces of South Africa. Four distinct species groups within the genus are proposed and diagnosed. An illustrated identification key to all described species of *Dinapsis* is provided. Online interactive Lucid keys to Afrotropical megalyrid genera and *Dinapsis* species are available at http://www.waspweb.org.

## ﻿Introduction

The Megalyridae comprise eight extant genera and five extinct genera (Shaw 1987, 1988, 1990a, 1990b, 2003, 2005; Mita et al. 2007; Poinar and Shaw 2007; Vilhelmsen et al. 2010). Two of these, *Dinapsis* Waterston, 1922 and *Megalyridia* Hedqvist, 1959 are endemic to the Afrotropical region (Waterston 1922; Hedqvist 1959, 1967; Shaw 1990b; van Noort and Shaw, 2009) and are the only two indigenous genera. The Australian species, *Megalyrafasciipennis* Westwood, 1832 was accidentally introduced into South Africa in freshly cut timber (Gess 1964; van Noort and Picker 2011). Prior to this revision two *Dinapsis* species, *D.turneri* Waterston, 1922 from South Africa and *Dinapsiscentralis* Shaw & van Noort, 2009 from Central African Republic, were the only known *Dinapsis* species from the African mainland (Waterston 1922; Shaw and van Noort 2009). Ten *Dinapsis* species have been described from Madagascar (Hedqvist 1967; Shaw and van Noort 2009; Madl 2010; Mita and Shaw 2020), but many undescribed species are known from Madagascar.

*Dinapsis* was originally placed in the family Dinapsidae, erected by Waterston (1922) when he described the genus. The extinct genus *Prodinapsis* Brues, 1923 described from Baltic amber, was also initially assigned to the family Dinapsidae (Brues, 1923), but both genera were subsequently transferred to the Megalyridae (Brues, 1933). Subsequent authors followed this classification, either treating the dinapsines as a tribe (Hedqvist 1959; Shaw 1990b) or a subfamily (Baltazar 1962; Rasnitsyn 1977). The fossil genus *Maimetsha* Rasnitsyn, 1975 from the late Cretaceous period was originally placed in a new family Maimetshidae (Rasnitsyn 1975) but was reassigned to the Megalyridae and later placed within the tribe Dinapsini (Shaw 1988, 1990b). However, Perrichot (2009) and Vilhelmsen et al. (2010) considered that *Maimetsha* and *Guyotemaimetsha* should be excluded from Megalyridae. The current classification divides the family Megalyridae into six tribes: Rigelini, Megalyridiini, Megalyrini, Prodinapsini, Dinapsini, and Cryptalyrini (Shaw 1990b). All extant Dinapsini are assigned to *Dinapsis* or *Ettchellsia* Cameron, 1909 (Shaw 1990b; Vilhelmsen et al. 2010; Mita and Shaw 2012; Chen et al. 2021). The family Megalyridae is regarded as monophyletic based on the presence of a pronotal spiracle (i.e., the anterior thoracic spiracle is externally visible and completely surrounded by pronotal cuticle, a character state that is shared with extant Ceraphronoidea, but in a few megalyrid fossils the spiracle has an opening towards the posterior margin of the pronotum); presence of well-defined subantennal grooves; and uniquely reduced hind wing venation with a short RS vein stub (Gibson 1985; Shaw 1988, 1990b; Mason 1993; Gauld and Hanson 1995; Mita et al. 2007; Poinar and Shaw 2007; Perrichot 2009; Vilhelmsen et al. 2010).

The hosts of most megalyrid genera and species are unknown, but available evidence suggests that they are idiobiont endoparasitoids of concealed insect larvae. The only detailed biological observations are for a few species of *Megalyra* (Shaw 1990a, b). The most common Australian species, *Megalyrafasciipennis* Westwood, parasitises large larvae of wood-boring Cerambycidae (Hacker 1913, 1915; Rodd 1951). One small Australian species, *Megalyratroglodytes* Naumann, 1987 attacks the larvae of mud-nesting Crabronidae on rock faces (Naumann 1983, 1987). The evolutionary transition from parasitism of wood-boring beetles to attacking aculeate wasp larvae has also evolved several times in the Ichneumonidae (Jussila and Kapyla 1975; Gauld 1988, 1991; Quicke 2015), a process likely to have followed a progression of host shifts, from beetles in wood to wasps nesting in wood, and subsequently a transition to wasps nesting in other situations (Shaw and van Noort 2009). The biology of all *Dinapsis* species is unknown.

We here revise the mainland African and Mauritian species of *Dinapsis* and provide identification keys to all the described *Dinapsis* species, including those named so far from Madagascar. There are numerous additional undescribed species from Madagascar that will be treated in a future revision. Images and online interactive Lucid identification keys to the Afrotropical megalyrid genera and *Dinapsis* species are available at WaspWeb (http://www.waspweb.org) (van Noort 2022).

## ﻿Materials and methods

As far as possible morphological terminology follows the Hymenoptera Anatomy Ontology project (HAO Portal hymao.org); where terms are not defined on HAO, Huber and Sharkey (1993), Mita and Shaw (2020), and Shaw (1990a, 2003) are followed. Microsculpture terminology follows Harris (1979).

The following abbreviations are used in this paper:

**F1-F12** sequential antennal flagellomere segments, basal to distal;

**LOL** lateral ocellar line, shortest distance between inner margins of median and lateral ocelli;

**OOL** ocular ocellar line, shortest distance from inner eye orbit and outer margin of posterior ocellus;

**POL** posterior ocellar line, shortest distance between inner margins of posterior ocelli;

**T1-T5** sequential tarsomere segments, basal to distal.

A leg from each of ten specimens of *Dinapsis*, representing seven species, was submitted to BOLD for DNA barcoding. DNA extracts were obtained from the legs at BOLD using a glass fibre protocol (Ivanova et al. 2006). Total genomic DNA was re-suspended in 30 μl of dH2O, a 658 base pairs (bp) region near the 5’ terminus of the CO1 gene was amplified using standard primers (LepF1–LepR1) following established protocols (http://v4.boldsystems.org/index.php), and a composite sequence was generated for all successful amplifications. All information for the sequences associated with each individual specimen barcoded can be retrieved from the Barcode of Life Data System (BOLD) (Ratnasingham and Hebert 2007).

Images were acquired at SAMC with a Leica LAS 4.9 imaging system, comprising a Leica Z16 microscope with a Leica DFC450 Camera and 0.63× video objective attached. The imaging process, using an automated Z-stepper, was managed using the Leica Application Suite v. 4.9 software installed on a desktop computer. Diffused lighting was achieved using a Leica LED 5000 Dome. Additional images were acquired at the University of Wyoming using Leica Application Suite (Leica Microsystems) and image stacking software, Combine ZM and Zeren Stacker v. 1.04. SEM images were acquired at UWIM using an Environmental Scanning Electron Microscope at the UW Microscopy Core Facility. Dry uncoated specimens were mounted on metal stubs for SEM study at low operating voltages and photography, then the glue was dissolved, and the specimens were remounted, undamaged, on pins and points with their original data. An SEM label was added to indicate which specimens were studied. All images included in this paper, as well as additional images, and online interactive keys to *Dinapsis* species are available on WaspWeb (https://www.waspweb.org) (van Noort 2022).

Lucid pathway and Lucid matrix keys were developed using Lucid Builder v. 4.0.23. Character matrices were generated and edited using Microsoft Excel; matrices were then used as input into Lucid matrix key production (Penev et al. 2009). The online interactive keys were produced using Lucid meeting the requirements of publishing both static and dynamic interactive keys under an open access model (Penev et al. 2009). All keys were illustrated using high quality annotated images, highlighting diagnostic characters. The images are integrated into the key above each couplet resulting in a user-friendly output. This key format reduces the requirement of familiarity with morphological terminology associated with a particular taxonomic group, because the characters are visually illustrated making the keys usable by a wide range of end-users including ecologists and conservationists. Online identification keys are presented in two different formats on WaspWeb: traditional static dichotomous keys where a choice needs to be made at each key couplet to continue, which are also presented as an interactive Lucid pathway (dichotomous) key; and Lucid matrix keys where relevant states from multiple character features can be selected independently until identification is achieved. For more information concerning Lucid keys visit http://www.lucidcentral.org.

Distribution maps were generated using SimpleMappr, an online data resource in the public domain (Shorthouse 2010).

### ﻿Depositories

**CAS**California Academy of Sciences, San Francisco, USA (Brian Fisher)

**DEBU**University of Guelph Insect Collection, Canada (Steven Paiero)

**ICIPE** International Centre of Insect Physiology and Ecology, Nairobi, Kenya (Robert Copeland)

**MNHN**Muséum National d’Historie naturelle, Paris, France (Agnièle Touret-Alby)

**MZLU**Zoologiska Museet Lunds Universitet, Lund, Sweden (Christer Hansson)

**NHMD** Natural History Museum of Denmark, University of Copenhagen, Denmark (Lars Vilhelmsen)

**NHMUK**The Natural History Museum, London, England (Natalie Dale-Skey)

**NMSA**KwaZulu-Natal Museum, Pietermaritzburg, South Africa (Tricia Pillay)

**NMKE**National Museum of Kenya, Nairobi, Kenya (Laban Njoroge)

**SAMC**Iziko South African Museum, Cape Town, South Africa (Simon van Noort)

**USNM**Smithsonian Institution, US National Museum of Natural History, USA (Matt Buffington)

**UWIM** UW Insect Museum, Laramie, USA (Scott Shaw)

## ﻿Systematics

### ﻿Identification keys

Standard dichotomous keys to genera of African Megalyridae and to the described species of *Dinapsis* are presented below. Online interactive Lucid pathway and Lucid matrix keys are available on WaspWeb (van Noort 2022). The LIF3 file for the online Lucid matrix key to all Afrotropical species of Megalyridae is provided as Suppl. material 1. Lucid Interchange Format v. 3 (LIF3) files are XML based files that store all the Lucid3 key data, allowing exchange of the key with other key developers such as Intkey (DELTA), or MX. The provision of this LIF3 data set allows future workers to edit the key and to add newly described taxa. The data file for the published key that is stored on the publisher’s website and in e-archives has the rights of “first publication” identified by its bibliography data, location, and citation (Sharkey et al. 2009). The concept of publication, citation, preservation, and re-use of data files to interactive keys under the open access model is detailed in Penev et al. (2009).

### ﻿Identification key to genera of African Megalyridae

**Table:** 

1	Mesoscutum with a weak median sulcus (A); propodeum with a shallow, longitudinal median sulcus (A); eyes not surrounded by an orbital carina (B); fore wing with veins 1mcu and Cu1 present (C)	***Megalyra* (one introduced species in South Africa: *Megalyrafasciipennis*)**
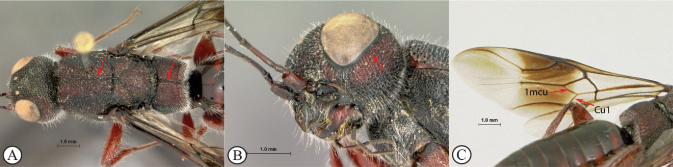		
–	Mesoscutum with a strong longitudinal median sulcus (a); propodeum without a longitudinal median sulcus (a); eyes surrounded by an orbital carina (b); fore wing veins 1mcu and Cu1 spectral (c, d)	**2**
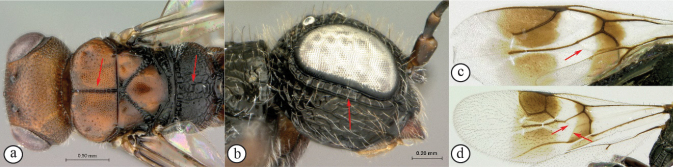		
**2**	Fore wing with one submarginal cell (A); radial vein reduced, radial cell open (A); propodeum rugulose without parallel longitudinal carinae (B)	***Megalyridia*** (**monotypical genus: *Megalyridiacapensis* is restricted to Eastern, Northern, and Western Cape provinces of South Africa)**
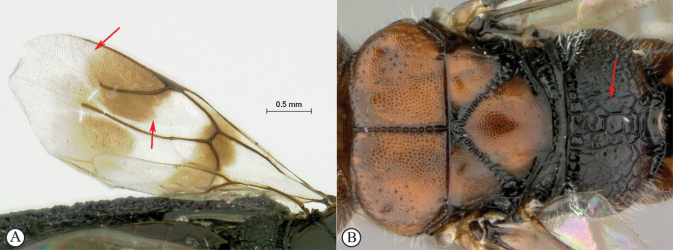		
–	Fore wing with two submarginal cells (a); radial cell closed (a); propodeum with distinctive pattern of six longitudinal, nearly parallel carinae, each tract with lateral transverse carinae that may be reduced in number (b)	***Dinapsis***
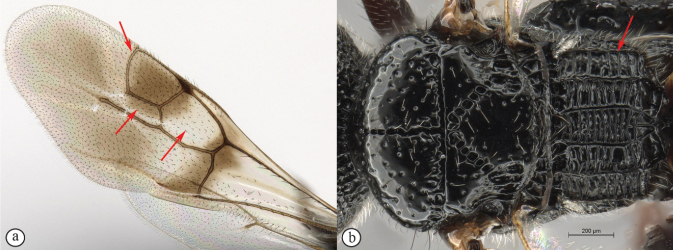		

#### ﻿ Dinapsis

Taxon classificationAnimaliaHymenopteraMegalyridae

Waterston

87539990-8FA9-5155-9110-C0F7A7CAADDD

##### Type species.

*Dinapsisturneri* Waterston, 1922, by original designation (South Africa, Western Cape, Ceres).

**Figure 1.**
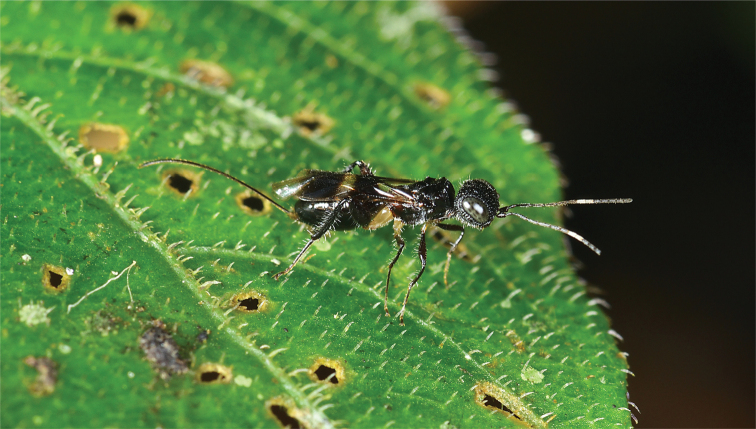
*Dinapsisalbicoxa* female (Ranomafana National Park, 21˚14'18.48"S, 47˚23'40.50"E, alt. 1138 m, Madagascar). Photograph copyright Steve Marshall (used with permission).

##### Diagnosis.

**Figure 2.**
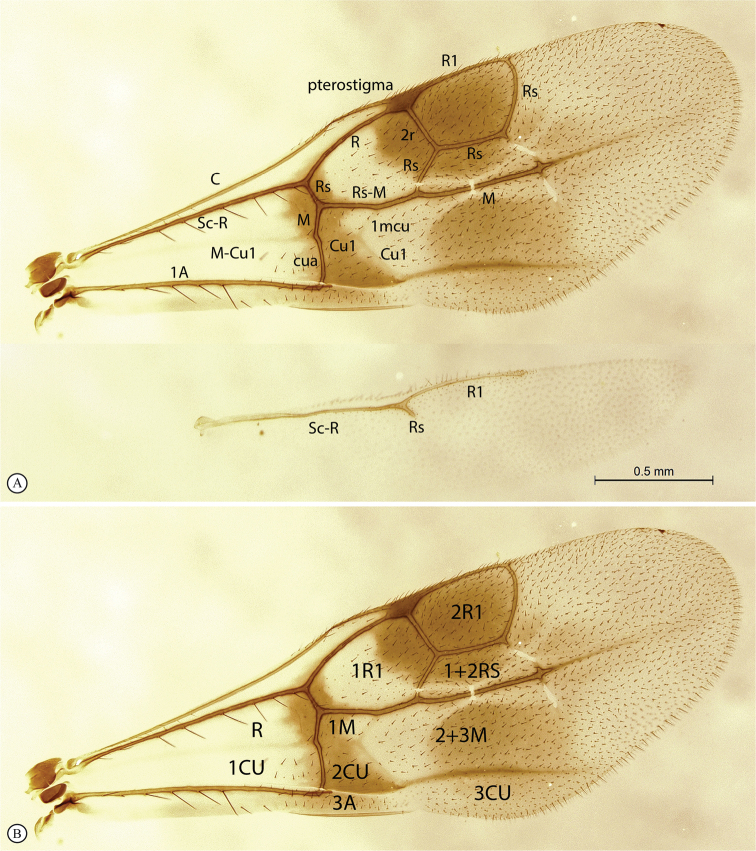
**A** venation names of fore and hind wing of *Dinapsisturneri* holotype **B** fore wing cell names of *Dinapsisturneri* holotype. The closed cell 2R1 with vein Rs reaching the wing margin is a synapomorphy for the genus *Dinapsis* within the Megalyridae.

*Dinapsis* species have a distinctive forewing venation pattern, with vein Rs curving abruptly to the anterior wing margin to form a short, abruptly truncate marginal cell (cell 2R1) (Fig. 2A, B) (Waterston 1922; Hedqvist 1967; Shaw 1990b). They also have a distinctive pattern of six longitudinal, nearly parallel, propodeal carinae (Figs 6E, 25D) (Waterston 1922; Hedqvist 1967; Shaw 1990b). However, the patterns and degree of development of the propodeal transverse carinae vary among species.

##### Recognition.

The genus *Dinapsis* can be keyed out from the two other megalyrid genera present in Africa using the above generic key that is also available as an online interactive Lucid key on WaspWeb (https://www.waspweb.org) (van Noort 2022).

##### Biology.

Unknown. Specimens are most commonly collected in Malaise traps, and less frequently in yellow pan traps, via tree canopy fogging, or through leaf litter sifting. To our knowledge only a single living specimen of this genus has ever been photographed (Fig. 1).

##### Species richness.

The genus *Dinapsis* was previously known from ten Madagascan and two continental African species (Waterston 1922; Hedqvist 1967; Shaw and van Noort 2009; Madl 2010; Mita and Shaw 2020). The current revision has elevated species richness of the genus to 19 named species:

*D.albicauda* Mita & Shaw, 2020 (Madagascar)

*D.albicoxa* Hedqvist, 1967 (Madagascar)

*D.bicolor* van Noort & Shaw, sp. nov. (South Africa)

*D.centralis* Shaw & van Noort, 2009 (Central African Republic, Cameroon, Kenya, Uganda)

*D.cresta* Mita & Shaw, 2020 (Madagascar)

*D.gamka* van Noort & Shaw, sp. nov. (South Africa)

*D.hirtipes* Hedqvist, 1967 (Madagascar)

*D.igneus* van Noort & Shaw, sp. nov. (Mauritius)

*D.luteus* Mita & Shaw, 2020 (Madagascar)

*D.nubilis* Hedqvist, 1967 (Madagascar)

*D.oculohirta* Hedqvist, 1967 (Madagascar)

*D.planifrons* Mita & Shaw, 2020 (Madagascar)

*D.scriptus* Mita & Shaw, 2020 (Madagascar)

*D.seyrigi* Hedqvist, 1967 (Madagascar)

*D.spinitibia* van Noort & Shaw, sp. nov. (Tanzania)

*D.taita* van Noort & Shaw, sp. nov. (Burundi, Kenya)

*D.tricolor* Shaw & van Noort, sp. nov. (Kenya, South Africa)

*D.turneri* Waterston, 1922 (South Africa)

*D.zulu* Shaw & van Noort, sp. nov. (South Africa)

##### Distribution.

Burundi, Cameroon, Central African Republic, Kenya, Madagascar, Mauritius, South Africa, Tanzania, Uganda (Figs 43, 44). The currently recorded distribution is a highly biased artefact due to under-sampling in the Afrotropical region (see discussion).

##### Barcoding.

Of the ten *Dinapsis* samples submitted to BOLD (representing seven species: *D.bicolor*, *D.gamka*, *D.igneus*, *D.taita*, *D.tricolor*, *D.turneri*, *D.zulu*) DNA was successfully extracted from seven specimens represented by five species (Table 1).

**Table 1.** Details of barcode data for the seven *Dinapsis* specimens from which DNA was successfully extracted.

*Dinapsis* species	Type status	Country	Sample ID	BOLD sequence code	BIN URI
***D.bicolor***	Holotype ♀	South Africa	38754_A03_SAM-HYM-P088338	FSA189521	BOLD:AEH7061
***D.igneus***	Holotype ♀	Mauritius	8754_A01_Din_ign_fem	FSA189321	None
***D.igneus***	Paratype ♂	Mauritius	38754_A02_Din_ign_mal	FSA189421	None
***D.taita***	Paratype ♀	Burundi	08672-MEGSPBURH9	KINS160911	BOLD:AAZ9109.
***D.tricolor***	Paratype ♀	Kenya	38754_A07_NMKE_Din_tric	FSA189921	None
***D.tricolor***	Paratype ♀	South Africa	38754_A08_NMSA-HYM-000546	FSA190021	None
***D.zulu***	Paratype ♀	South Africa	38754_A09_NMSA-HYM-000539	FSA190121	None

### ﻿*Dinapsis* species groups

***Dinapsishirtipes* species group**. This species-group was proposed by Mita and Shaw (2020) for a set of distinctive species from Madagascar with unusual head characteristics. The face is flat, and the dorsum of the head is raised such that the vertex and occiput are separated by a sharp collar-like division (see key couplet 1, images A, B). These modifications occur in both females and males but are more pronounced in females. This group consists of *D.hirtipes* and five recently described species (*D.albicauda*, *D.cresta*, *D.luteus*, *D.planifrons*, and *D.scriptus*), all from Madagascar. None has yet been found on the African mainland. The raised vertex is unique to this group of species and is considered as a synapomorphy supporting monophyly of this group. For more information on this species group see Mita and Shaw (2020).

***Dinapsisoculohirta* species group**. In this group of species, the eyes are “hairy,” being covered with numerous small setae (Fig. 19E). Eye setae occur in both males and females. The characteristic of setose eyes in *Dinapsis* was first mentioned by Hedqvist (1967), who used the feature to distinguish *D.oculohirta* from other species known from Madagascar at that time. Shaw and van Noort (2009) showed that the condition of setose eyes also occurs in *D.centralis* from the Central African Republic.

In this paper we define the *oculohirta* species group as comprising *D.oculohirta* from Madagascar, *D.centralis* from central Africa, as well as seven new species from the African mainland and Mauritius described below (*D.bicolor*, *D.gamka*, *D.igneus*, *D.spinitibia*, *D.taita*, *D.tricolor*, and *D.zulu*). *Dinapsisoculohirta*, described from Madagascar, has not been found on the African mainland yet. Otherwise, the named species in this group are from the African mainland. However, additional new species in this group from Madagascar are planned to be covered in a separate paper.

Despite these species being morphologically similar, this group is probably a paraphyletic grade, and may be just a convenient way to group many basal species in the genus. Hairy eyes also occur in the sister-genus *Ettchellsia* from south-east Asia, so out-group comparison suggests that the setose eyes of this species group are a plesiomorphic condition, and perhaps the lack of ocular setae (bare eyes) is a synapomorphy uniting the three other *Dinapsis* species groups. The species in the *oculohirta* group, if not comprising a monophyletic lineage, are at least inferred to be the most basal lineages in the genus.

The South African species *Dinapsisturneri* Waterston has for many years been difficult to assign into a group with other species, especially since until now it was known only from the holotype specimen. Morphologically the holotype of *D.turneri* looks similar to species in the *oculohirta* species group but the eyes do not appear to have setae. This point is debatable because the eyes of the holotype specimen are oddly shrunken and it is hard to observe whether setae are present or not (see Figs 34C, 34D, 34E). Setae, if present originally, might have been lost. Freshly collected specimens presented in this paper agree with the holotype of *D.turneri* in all respects, except that they do have setae on the eyes (Fig. 37E). Therefore, we conclude that the apparent lack of setae in the holotype specimen is due to post-mortem damage and subsequent loss, and we also assign *D.turneri* to the *oculohirta* species group.

***Dinapsisnubilus* species group.** In this species group the mesoscutum has distinctive lobe-like protuberances postero-laterally (see key couplet 4, images A, B). These lobes are parts of a projecting carina running obliquely on the lateral mesoscutal surface that appear lobe-like in dorsal view. A few species assigned to other groups (such as *D.seyrigi* and *D.tricolor*) to a lesser extent have the same carina projecting somewhat, but in these it is not so lobe-like in dorsal view. This feature is shared with *Dinapsisalbicoxa* Hedqvist, the only other species now assigned to this small, but distinctive group. This group is known only from Madagascar.

***Dinapsisseyrigi* species group**. In this species group the gena behind the orbital carina is smooth (see key couplet 3, image A). This group consists of the typical *Dinapsisseyrigi* and several other undescribed species from Madagascar. None has yet been found on the African mainland. The species are not frequently collected, but the smooth lateral areas of the head are quite distinctive. Some of the undescribed species have distinctive patches of silver setae on the sides of the mesopleuron. This is probably a monophyletic group (other groups all have very coarse sculpture on the sides of the head).

### ﻿Key to species of *Dinapsis*

**Table:** 

1	Vertex produced dorsally forming a raised carina (A, B); subquadrate in anterior view (A) (*Dinapsishirtipes* Hedqvist species group)	**14**
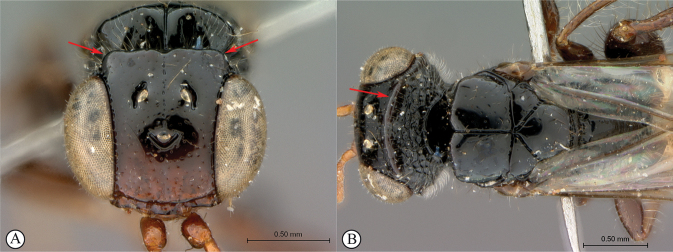		
–	Vertex normal, evenly rounded (a, b)	**2**
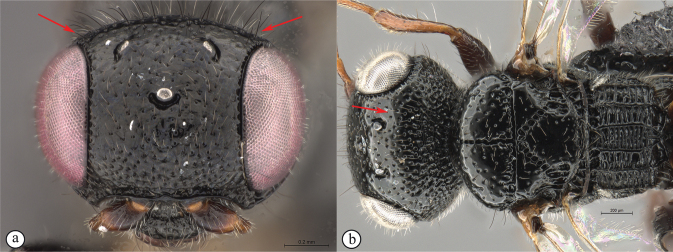		
2	Two large obvious sharply-pointed spines in close apposition on hind tibial apex (A); mesoscutum with sharply-projecting wave-shaped lateral teeth and strong transverse ridges (B)	***Dinapsisspinitibia* van Noort & Shaw, sp. nov.**
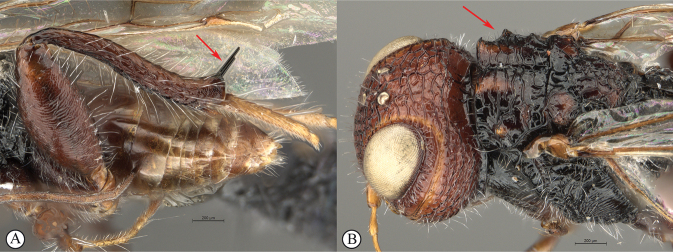		
–	Hind tibia without apical spines (a); mesoscutum laterally rounded, mesoscutal transverse ridges absent (b)	**3**
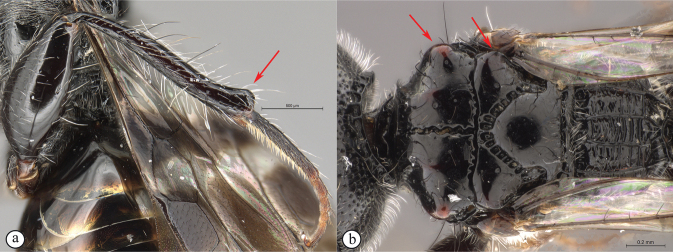		
3	Gena behind the orbital carina smooth (A); vertex polished, with only a few scattered minute punctures and setae (B); propodeum medially polished, mostly lacking transverse carinae between the submedian longitudinal carinae (B)	***Dinapsisseyrigi* Hedqvist**
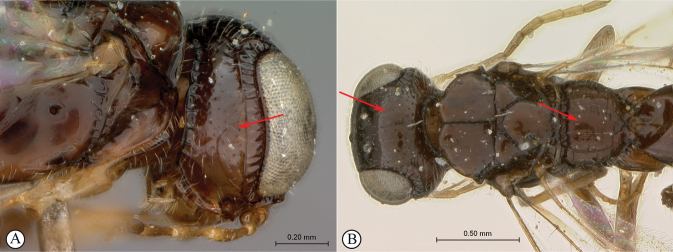		
–	Gena behind orbital carina sculptured, or densely punctate (a); vertex often sculptured (a), if smoother then with denser punctation and setae (b); propodeum medially usually with well-developed transverse carinae between the submedian longitudinal carinae (b)	**4**
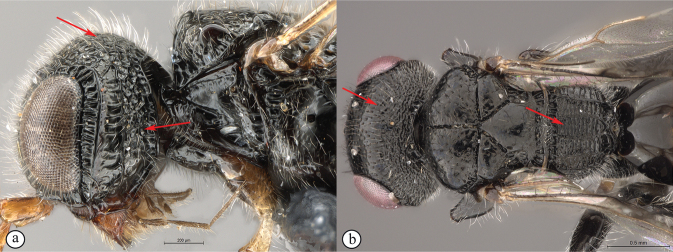		
4	Mesoscutum with a distinctive and strongly projecting carina (lateral mesoscutal margin projected as a flange) situated posterolaterally that appears as a lobe-like lateral protuberance in dorsal view (A, B); mesoscutum always smooth (A)	**5**
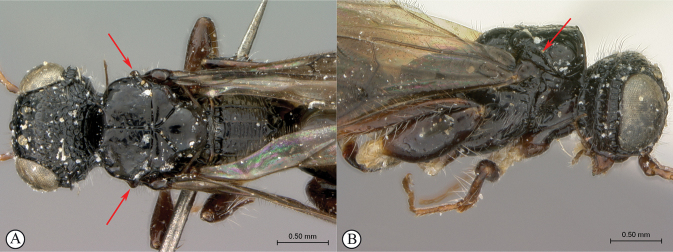		
–	Mesoscutum without a strongly projecting carina situated postero-laterally (a), or if carina slightly projecting (b) then adjacent mesoscutal surface with large fovea (b)	**6**
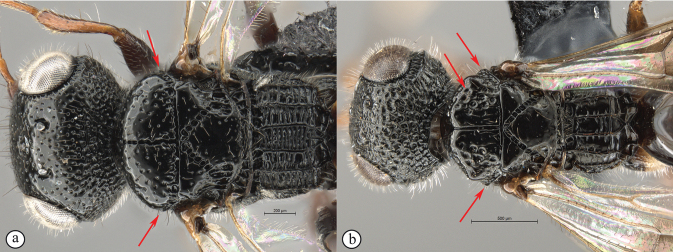		
5	Wings infuscate (brownish) without obvious bands (A); head weakly foveate-reticulate, face and vertex with polished areas between fovea, setae sparse and short (B)	***Dinapsisnubilis* Hedqvist**
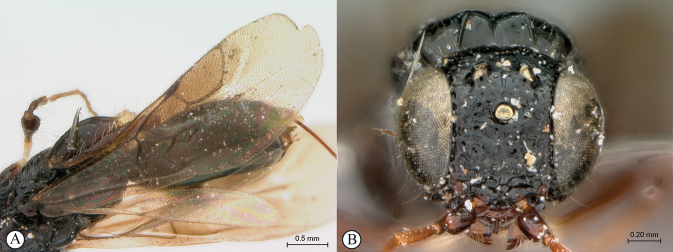		
–	Wings with transverse dark bands (a); head coarsely reticulate, with long setae (b)	***Dinapsisalbicoxa* Hedqvist**
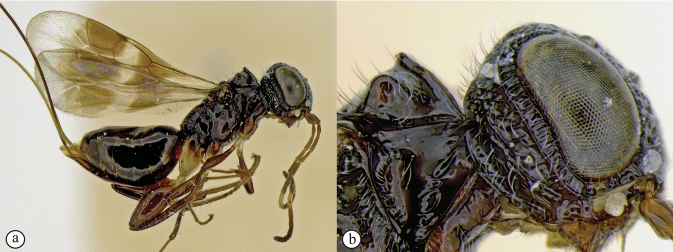		
6	Vertex with a medial row of punctures, which may be fused to form a shallow groove, between ocelli (A, B)	***Dinapsisoculohirta* Hedqvist**
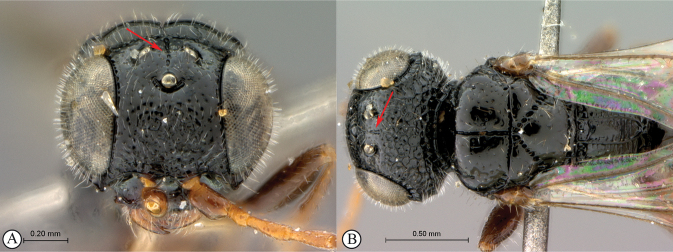		
–	Vertex without a medial row of punctures between ocelli (a, b)	**7**
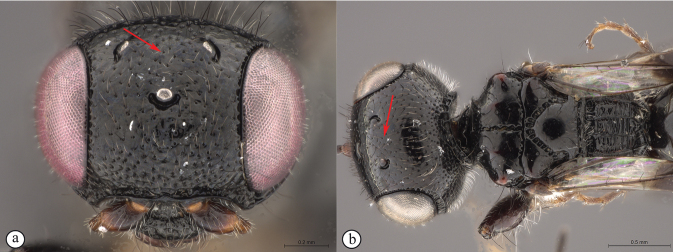		
7	Vertex and mesoscutal lobes coarsely rugulose-reticulate (A); scutoscutellar sulcus demarcated by broad, longitudinally striate furrow (A); flagellum uniformly dark, or occasionally lighter in basal 1/3 (B)	***Dinapsiscentralis* Shaw & van Noort**
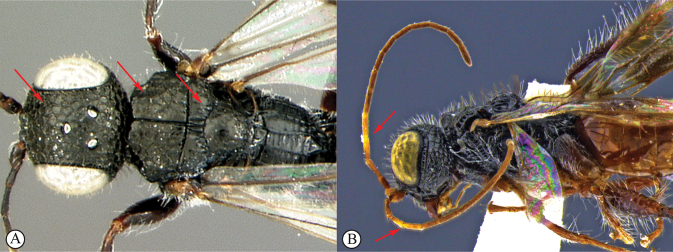		
–	Vertex finely or coarsely punctate, mesoscutal lobes polished with sparse punctures or scattered fovea (a); scutoscutellar sulcus demarcated by foveate furrow (a); flagellum often with medial white band (b)	**8**
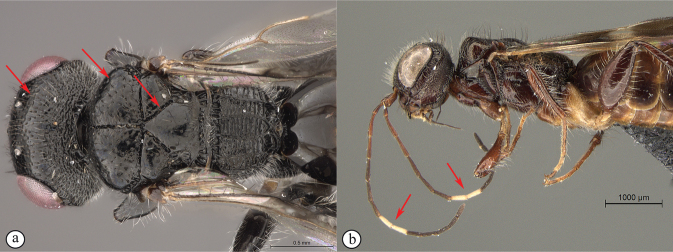		
8	Mesoscutum projecting anteriodorsally as a bilobed crest (A), distinct in lateral view (B); head and mesosoma densely rugulose-punctate, with metallic greenish bronze sheen (requires good lighting to discern) (A, B)	***Dinapsisigneus* van Noort & Shaw, sp. nov.**
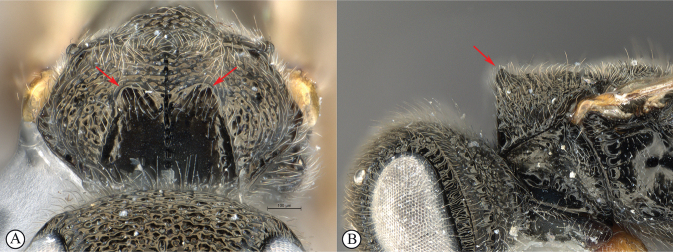		
–	Mesoscutum not projecting dorsally (a, b); mesosoma polished or punctate, sometimes with scattered foveae, non-metallic (a, b)	**9**
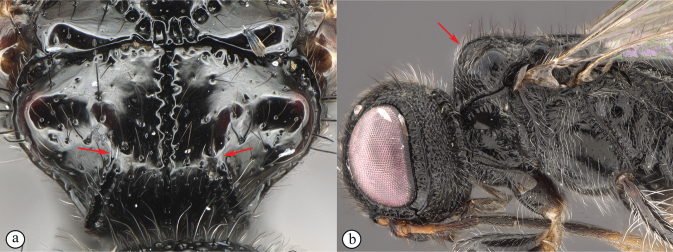		
9	Metasoma pale orange-brown, contrasting with black head and mesosoma (A, B); ovipositor short, subequal in length to metasoma (B)	***Dinapsisbicolor* van Noort & Shaw, sp. nov.**
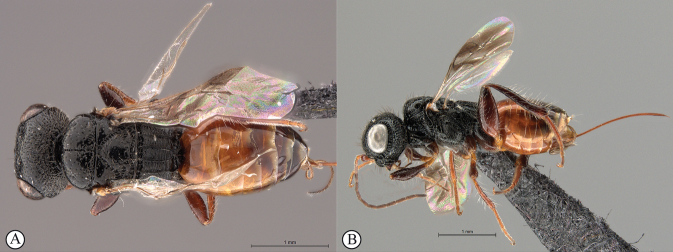		
–	Metasoma black or dark brown, body unicolourous (a, b), mesoscutum may have small orange patches; ovipositor long, as long as mesosoma and metasoma combined (b)	**10**
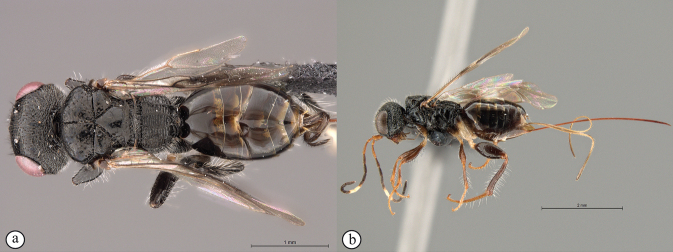		
10	Vertex coarsely punctate (A, B); dorsal mesoscutal areas laterally with fovea, contrasting with polished medial mesoscutal area (A, B)	**11**
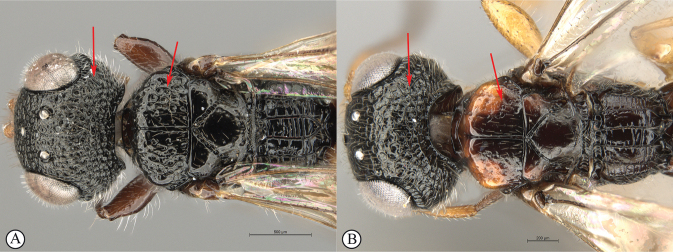		
–	Vertex finely punctate (a, b); dorsal mesoscutal areas laterally without fovea, polished with fine punctation (a, b)	**12**
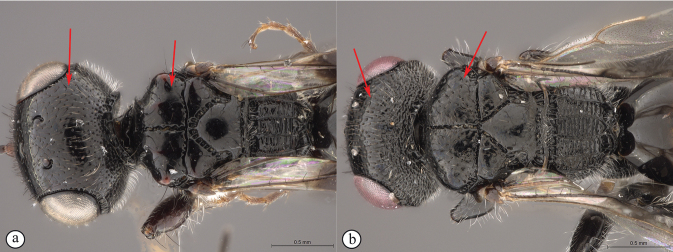		
11	Fovea on dorsal mesoscutal areas indistinct, small (A); scutoscutellar sulcus demarcated by narrow furrow with fovea indistinct (A); mesoscutum with orange patches (A, B)	***Dinapsistaita* van Noort & Shaw, sp. nov.**
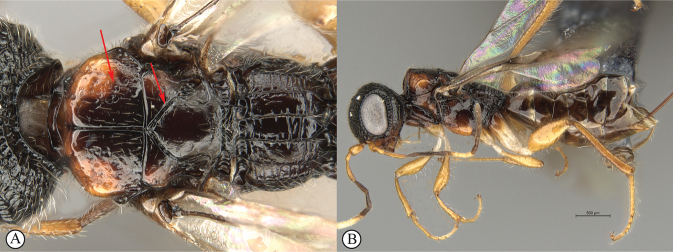		
–	Fovea on dorsal mesoscutal areas large and distinct (a); scutoscutellar sulcus demarcated by strongly foveate furrow (a); mesoscutum uniformly black (a)	***Dinapsistricolor* Shaw & van Noort, sp. nov.**
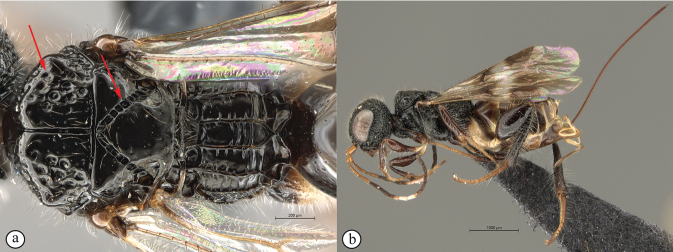		
12	Forewing with narrow dark bands, basal band absent from costal cell (A); apical infuscation diffuse (A); short, dark setae on dorsal surface of mesoscutum (A); scutoscutellar sulci meet the transscutal articulation independently (B)	***Dinapsisturneri* Waterston**
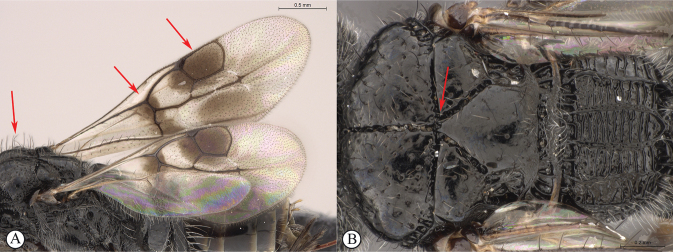		
–	Forewing with broad dark bands, basal band extending to anterior wing margin through costal cell (a); apical infuscation darker, obviously demarcated subparallel to apical wing margin (a); longer, dark setae on dorsal surface of mesoscutum; scutoscutellar sulci meet before reaching the transscutal articulation (b)	**13**
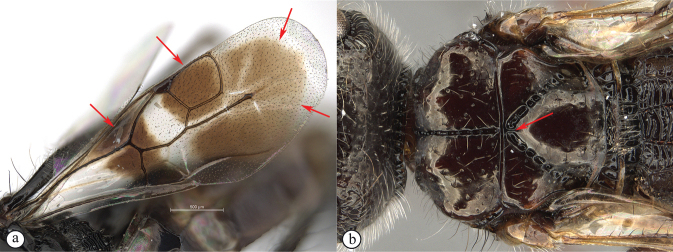		
13	Head small, height in lateral view equivalent to mesosomal height (A); postocular furrow broad, 1/2 genal width behind postocular carina, quarter of eye width (B)	***Dinapsiszulu* Shaw & van Noort, sp. nov.**
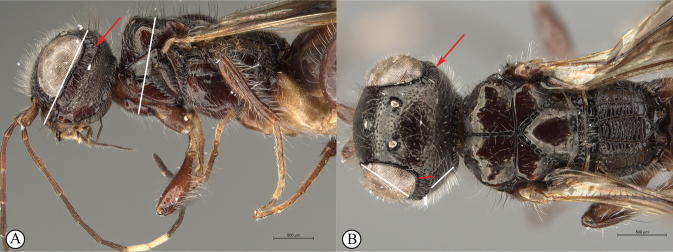		
–	Head large, height in lateral view 1.5× greater than mesosomal height (a); postocular furrow narrow, 1/5 of genal width, 1/7 of eye width (b)	***Dinapsisgamka* van Noort & Shaw, sp. nov.**
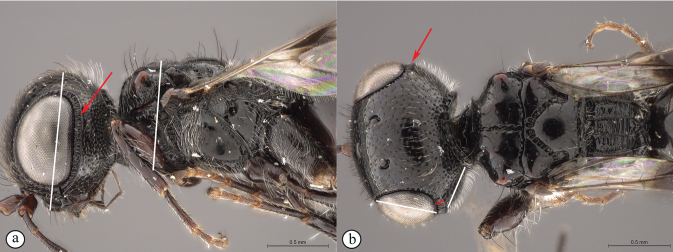		
14	Distal prong present on hind tibia (A, B)	**15**
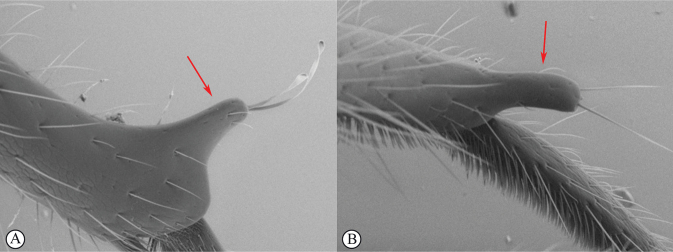		
–	Distal prong absent on hind tibia (a, b)	**16**
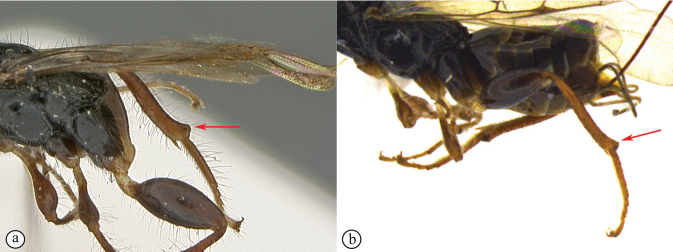		
15	Setae on distal prong spatulate (A); female body mostly brownish (B)	***Dinapsisplanifrons* Mita & Shaw**
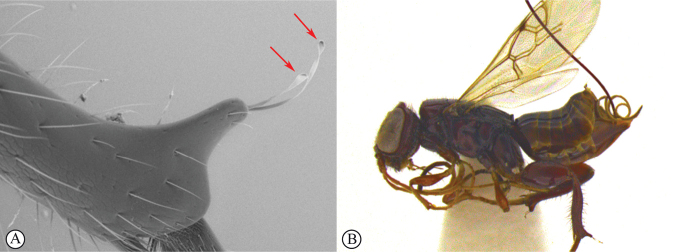		
–	Setae on distal prong simple (a, b); female body mostly blackish (b)	***Dinapsisscriptus* Mita & Shaw**
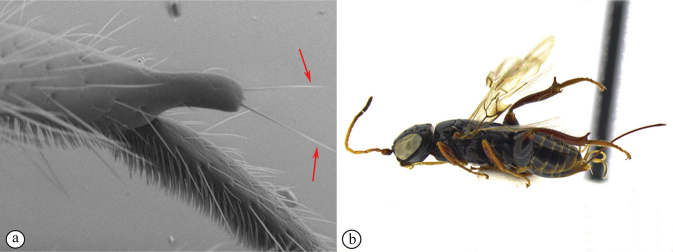		
16	Transverse carina on vertex strongly developed (A, B), width of carina wider than minimum distance between inner eye margins in anterior view (A)	**17**
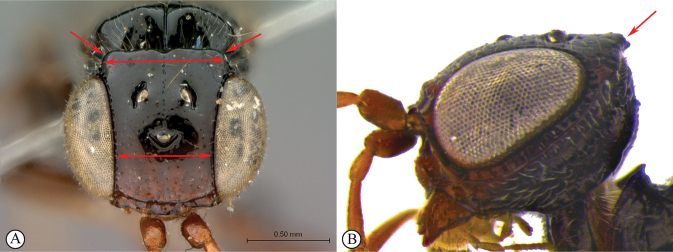		
–	Transverse raised carina on vertex less developed (a, b), width of carina narrower than minimum distance between inner eye margins in anterior view (b)	**18**
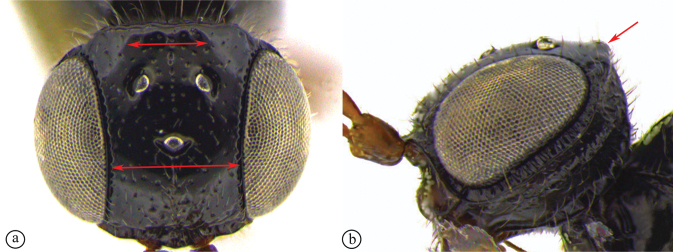		
17	Forewing with dark bands; posterior margin of raised vertex (A) and anterior margin of mesoscutum excavated medially (B)	***Dinapsishirtipes* Hedqvist**
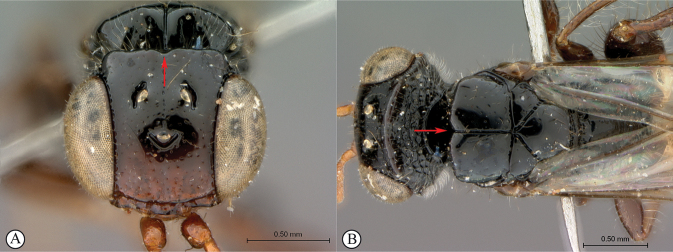		
–	Forewing without dark band; posterior margin of raised vertex (a) and anterior margin of mesoscutum flat (b), at most slightly excavated medially	***Dinapsisluteus* Mita & Shaw**
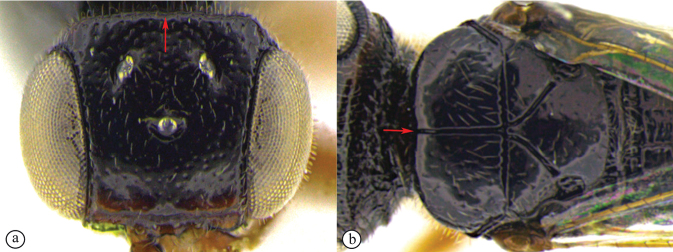		
18	Head round, as long as wide in lateral view (A); dorsal surface of head rounded in lateral view (A); ovipositor whitish excluding brown apex (B)	***Dinapsisalbicauda* Mita & Shaw**
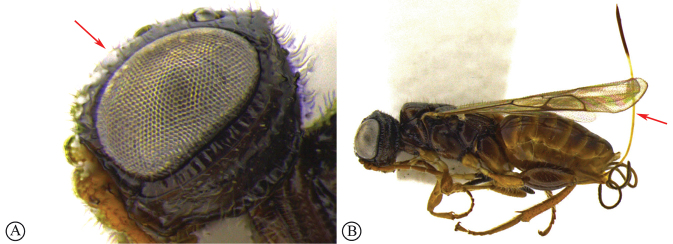		
–	Head oval, longer than wide in lateral view (a); dorsal surface of head less rounded in lateral view (a); ovipositor entirely brown (b)	***Dinapsiscresta* Mita & Shaw**
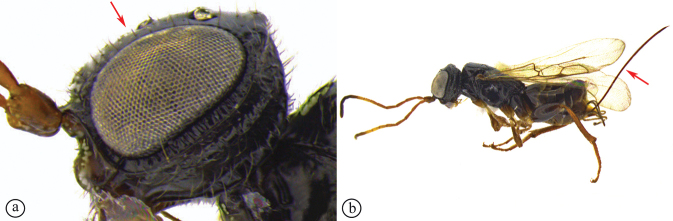		

#### ﻿ Dinapsis albicauda

Taxon classificationAnimaliaHymenopteraMegalyridae

Mita & Shaw, 2020

7BA1DCFB-A9FE-5C03-B1D8-A6306526582F

##### Material examined.

***Holotype***. Madagascar • ♀; Fianarantsoa Prov., Forêt d’Atsirakambiaty, 7.6 km 285° WNW Itremo; 20°35'36"S, 046°33'48"E; 1550 m a.s.l.; 22–26 Jan. 2003; Fisher, Griswold et al. leg.; California Acad. of Sciences; yellow pan trap; in montane rainforest; code: BLF7154 (CAS).

##### Distribution.

(Fig. 43) Madagascar.

##### Comments.

*Dinapsisalbicauda* is known only from the type-locality. *Dinapsisalbicauda* is similar to *D.cresta* Mita & Shaw but may be distinguished by its ovipositor being white-coloured basally, and by its head being rounded in lateral view. For a full species description and more information on its distribution and distinguishing characteristics, see Mita and Shaw (2020).

#### ﻿ Dinapsis albicoxa

Taxon classificationAnimaliaHymenopteraMegalyridae

Hedqvist 1967

DD368064-1952-5036-B8B6-8F08328BF7E6

Figs 1 , 3

##### Material examined.

**Figure 3.**
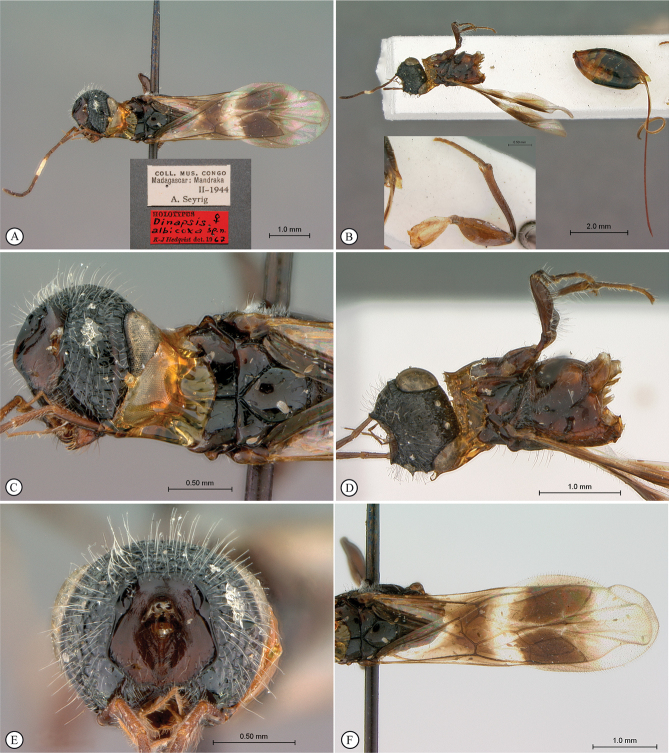
*Dinapsisalbicoxa* holotype female (MNHN). Note: at some point this specimen was broken and repaired incorrectly, with the face glued to the mesosoma **A** habitus, dorsal view **B** habitus, lateral view **C** head (lateral view), mesosoma, dorsal view **D** head, dorsal view, mesosoma lateral view **E** head, posterior view **F** wings. Scale bars: 1000 µm (**A, D, F**); 2000 µm (**B**); 500 µm (**C, E**).

***Holotype***. Madagascar • ♀; Mandraka; Feb. 1944; A. Seyrig; MNHN. Note: at some point this specimen was broken and incorrectly repaired with the head glued on backwards (the face is glued to the mesosoma) (see Fig. 3A).

##### Additional non-type specimens examined.

Madagascar • 1 ♀; Fianarantsoa Province, 7 km W Ranomafana,-21.263064°, 47.383935°; 1100 m a.s.ls; 22–31 October 1988; W.E. Steiner leg.; Malaise trap in small clearing; montane rain forest; USNM • 1 ♀, Ivondro; -18.238933, 49.366374; Dec. 1938; A. Seyrig leg.; MNHN • 1 ♀ Ranomafana National Park; 10 Oct. 2014; 21°14'18.48"S, 47°23'40.50"E; 1138 m a.s.l.; Map Datum: WGS 84; photo only; photographed by Steve Marshall (Fig. 1).

##### Distribution.

(Fig. 43) Madagascar.

##### Comments.

*Dinapsisalbicoxa* is currently only known from a few localities in southeast Madagascar. The holotype locality (Mandraka) is situated east of Manjakandriana (Antananarivo Province) There is a tiny settlement (-18.905361, 47.920253) 10 km east of Manjakandriana near the Mandraka Catholic church. The Mandraka River runs through this region and there is a Mandraka waterfall in the area. Seyrig may have been referring to the river/waterfall area rather than the precise settlement. Ivondro is located in the Atsimo-Atsinanana region.

*Dinapsisalbicoxa* is a distinctive species that is named for its white hind coxa (Fig. 1). The hind coxa is entirely white in the holotype, as well as the specimen examined from Fianarantsoa; however, the specimen from Ivondro has some brown colour basally and is not entirely white. To avoid confusion with any other *Dinapsis* species that may have white colour on the hind coxa apically, it is important to examine the characteristics of the mesoscutum laterally. Specimens of *D.albicoxa* have the mesoscutum with a strongly projecting lobe-like carina situated posterolaterally (Fig. 3C) and darkly banded wings (Fig. 3F). It should be noted that while the entirely white hind coxa is a useful diagnostic feature for *D.albicoxa*, there are several unrelated, undescribed species in the *oculohirta* species group that also have the hind coxa mostly white.

#### ﻿ Dinapsis bicolor

Taxon classificationAnimaliaHymenopteraMegalyridae

van Noort & Shaw sp. nov.

E5DFB122-571B-556D-A218-C244A83E96E4

https://zoobank.org/F43563FF-0017-45AC-9784-BBE3F750FDCB

Figs 4 , 5

##### Material examined.

***Holotype***. South Africa • ♀; Northern Cape, Tankwa National Park, Renoster River; 490 m a.s.l.; 32°14.704'S, 20°05.824'E; 17 Aug.–9 Sep. 2014; S. van Noort leg.; Malaise trap; *Acaciakaroo* thicket; Tanqua Wash Riviere; Succulent Karoo; TKW14-ACA1-M07; IMAGED WaspWeb LAS 4.9 SAMC 2019 (yellow label); HOLOTYPE *Dinapsisbicolor* van Noort & Shaw (red label); SAM-HYM-P088338; SAMC (DNA barcode sequence BOLD: FSA189521). ***Paratypes***. South Africa • 1 ♀; Northern Cape, Avontuur Farm, 16 km NW Nieuwoudtville; 31°16.249'S, 19°02.900'E; 764 m a.s.l.; 27 Oct. 2009–9 Feb. 2010; S. van Noort leg.; Malaise trap; Bokkeveld Sandstone Fynbos; GL07-FYN1-M144; SAM-HYM-P040214; SAMC • 1 ♀; Northern Cape, Tankwa National Park, Renoster River; 32°14.704'S, 20°05.824'E; 490 m a.sl.; 10 Jul.–17 Aug. 2014; S. van Noort leg.; Malaise trap; *Acaciakaroo* thicket; Tanqua Wash Riviere; Succulent Karoo; TKW14-ACA1-M04; SAM-HYM-P048086; SAMC • 1 ♀; same data as holotype except for: SAM-HYM-P048086; SAMC • 3 ♀♀; same data as holotype except for: SAM-HYM-P047813; SAMC • 7 ♀♀; same data as holotype except for: 17 Aug.–9 Sep. 2014; TKW14-ACA1-M07; SAM-HYM-P047814; SAMC • 1 ♀; same data as holotype except for: 9 Sep.–9 Oct. 2014; TKW14-ACA1-M09; SAM-HYM-P047815; SAMC • 1 ♀; same data as holotype except for: 9 Oct.–5 Nov. 2014; TKW14-ACA1-M11; SAM-HYM-P047816; (SAMC) • 14 ♀♀; same data as holotype except for: 22 Jun.–24 Sep. 2015; TKW14-ACA1-M19; SAM-HYM-P088324 to SAM-HYM-P08837; SAMC.

##### Diagnosis.

**Figure 4.**
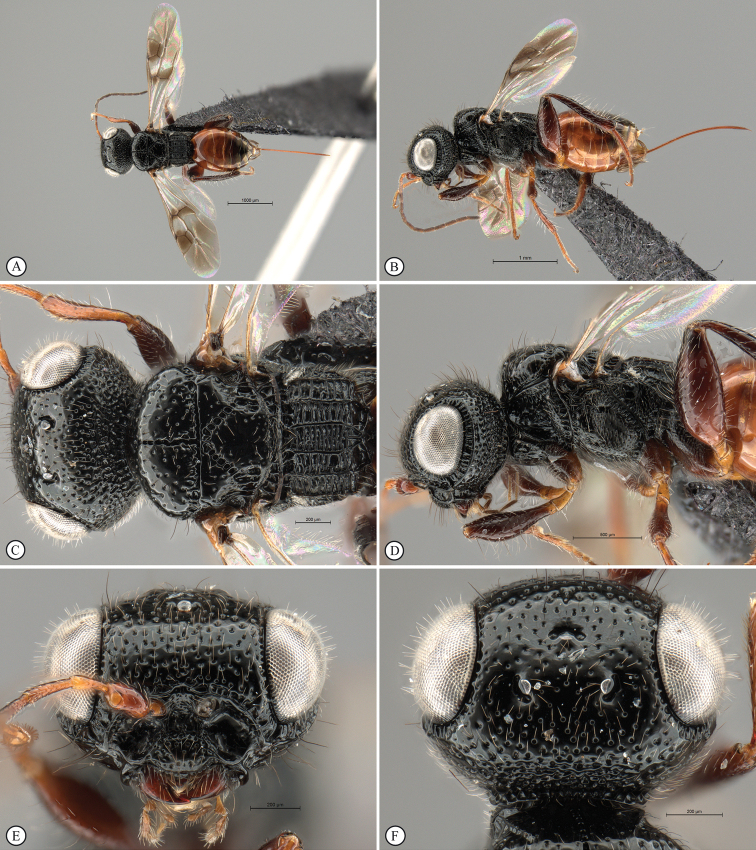
*Dinapsisbicolor* van Noort & Shaw, sp. nov. holotype female SAM-HYM-P088338 (SAMC) **A** habitus, dorsal view **B** habitus, lateral view **C** head, mesosoma, dorsal view **D** head, mesosoma, lateral view **E** head, anterior view **F** head, dorsal view. Scale bars: 1000 µm (**A, B**); 200 µm (**C, E, F**); 500 µm (**D**).

This species is easily recognised by the distinctly bi-coloured body (Fig. 4A, B): the metasoma is orange-brown contrasting with the black head and mesosoma, a unique colour pattern among the described species of *Dinapsis* (most species are overall black, but some undescribed Madagascan species have an orange head and mesosoma). The species falls within the *D.oculohirta* species group possessing the following diagnostic character states: eyes setose; face and mesosomal sculpture sparsely punctate; postocular orbital carina absent; antero-lateral corners of mesoscutum smoothly rounded without tubercles; scutoscutellar sulci comprising a line of adjacent large fovea, anteriorly meeting before reaching transscutal articulation; wing veins strong, wing banding light-brown, fainter than in other species with banding.

##### Distribution.

(Fig. 44) South Africa (Northern Cape Province).

##### Comments.

*Dinapsisbicolor* is probably also present in the Western Cape, as the Tankwa type locality is situated directly on the border between these two provinces. This is an arid-adapted species, collected from low rainfall regions in the Succulent Karoo and Bokkeveld Sandstone Fynbos, reflected in the orange colour of the metasoma a common colour present on the body of wasp species from arid regions.

**Figure 5.**
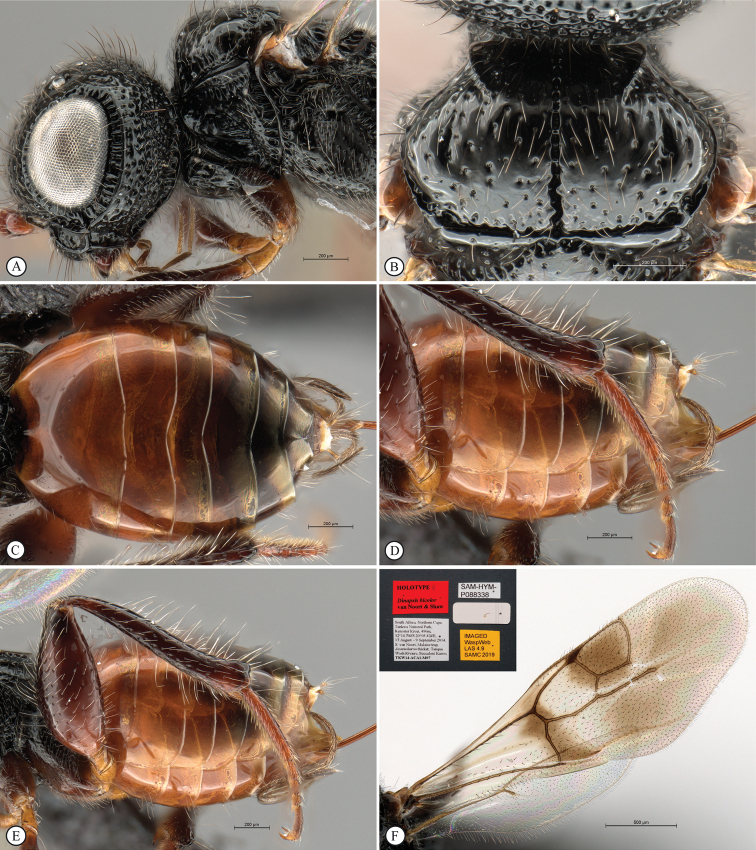
*Dinapsisbicolor* van Noort & Shaw, sp. nov. holotype female SAM-HYM-P088338 (SAMC) **A** head, mesosoma, lateral view **B** mesoscutum, anterodorsal view **C** metasoma, dorsal view **D** metasoma, lateral view **E** hind leg, lateral view **F** wings, dorsal view (inset: data labels). Scale bars: 200 µm (**A-C, D, E**); 500 µm (**F**).

##### Etymology.

This species is named for the contrasting colour between the metasoma and the rest of the body. Noun in apposition.

##### Barcode sequence for holotype specimen.

38754_A03_SAM-HYM-P088338 (sequence code in BOLD:FSA1895-21) BIN URI: BOLD:AEH7061.

##### Nucleotide sequence.

TTTATTGGGTCGTCTATAAGTATAATTATTCGGATAGAATTAAGGGTTCCTGGTTCATTTATTGGTAATGATCAAATTTATAATTCAATTGTTACAGCTCATGCTTTTATTATAATTTTTTTTATAGTTATACCTTTTATAATAGGAGGTTTTGGTAATTGATTGTTACCATTAATATTAGGAGCTCCTGATATATCTTATCCACGTTTAAATAATTTAAGATTTTGATTATTAATTCCTTCTTTATTATTTTTATTAATAAGATTTTATGTAGGTGGTGGTACAGGTACAGGGTGAACTGTGTATCCCCCATTGTCTTCAAATATATTTCATTCTGGAATAAGGGTAGATCTATCAATTTTTAGATTACATTTAGCTGGAATTTCATCAATTTTAGGCTCAGTTAATTTTATTTCAACAATTTTAAATTTGCGTAATATTAAATTATCAGTAAGTAATTTAAGGTTATTTATTTGATCAGTGTTTTTAACAGCTATTTTATTATTATTATCTTTACCTGTATTAGCAGGGGCTATTACTATATTGTTAACGGATCGTAATTTAAATACAACATTTTTTGACCCTTCAGGGGGCGGTGATCCAATTTTATATCAACATTTATTT.

##### Description.

**Holotype female. *Body*** length 3.5 mm excluding ovipositor.

***Colour*.** Head and mesosoma black with minute white setae, interspersed with darker longer setae; metasoma orange-brown, dorsally darker on last three tergites. Scape, pedicel, F1, F2, fore coxa, trochanters, tibiae, tarsi, ovipositor dark orange-brown; mandibles and hind leg reddish brown. Remaining antennal segments, fore and mid coxae and femora, wing venation, and ovipositor sheath dark brown. Eyes and ocelli silvery. Wing membrane clear except two dark brown pigmented bands across forewing with the apical band extending in a lighter infuscation almost to apical margin, and all the way to posterior margin in apical 1/2 of wing.

***Head*** oval, 1.44 × wider than height; vertex, frons, and face evenly sparsely punctate, interstices polished and 1–3 × greater than puncture width; ocelli small, OOL 3.0 × ocellar diameter; ocellar triangle equilateral; eye large and slightly protuberant, nearly parallel in anterior view, but diverging slightly ventrally; eye densely and evenly covered with minute white ocular setae; eye margined posteriorly by foveate groove; postocular orbital carina absent; antenna with 11 flagellomeres having flagellar length/width ratios as follows: F1 = 3.5, F2 = 3.0, F3-F11 = 2.5, F12 (apical flagellomere) = 3.0; apical flagellomere same width as basal flagellomeres; temple adjacent to ocular orbital carina punctate and polished, becoming areolate-reticulate towards occipital carina, temple width 0.88 × eye width in lateral view; malar space length 1.0 × mandible width basally; occiput areolate-reticulate, occipital carina wide and crenulate.

***Mesosoma*.** Pronotum polished, laterally excavated with a row of large oblong foveae situated dorsally and posteriorly; the mesoscutal anterior plate polished, with a medial row of punctures, and a lateral carina bounding a foveate groove; mesoscutum as long as wide, mesoscutal lobes sparsely punctate, polished, antero-lateral corners smoothly rounded without tubercles; medial mesoscutal furrow jagged with foveae; transscutal articulation a smooth furrow, anterior edge crenulated, posterior edge smooth; scutoscutellar sulci comprising a line of adjacent large foveae, anteriorly meeting before reaching transscutal articulation; scutellar disc medially polished, with scattered punctures laterally; scutellar disc medially devoid of setae, peripherally rimmed with erect setae; mesopleuron antero-laterally strongly foveate and sparsely setose, medially polished with sparse punctures and setae, and with large median mid-pit; propodeum medially with strongly developed transverse carinae between submedian longitudinal carinae, progressively less transverse carinae present from central to lateral longitudinal tracks.

***Legs*.** Apex of fore tibia with comb of 13 or 14 stout spines; hind coxa sparsely punctate, weakly covered with long, silky, white setae not obscuring surface; hind femur stout, polished, 2.6 × longer than wide, outer surface of hind femur sparsely, but evenly covered with long, erect, white setae, inner surface of hind femur polished sparsely punctate with very short setae; surface of hind tibia polished, sparsely punctate with long erect white setae dorsally and ventrally, shorter adpressed setae laterally; dorsal setae lacking spatulate tips; inner median margin of hind tibia with a dense longitudinal patch of shorter white setae; hind basitarsus long, subequal in length to remaining four tarsomeres combined; basitarsus ventrally with sparse preening brush consisting of numerous short, white setae, inclined posteriorly; basitarsus dorsally with normal hair-like setae, lacking spatulate tips; T2, T3, and T4 each short, T2 twice as long as wide, T3 and T4 ca. as long as wide; all tarsomeres with normal hair-like setae; tarsal claw simple, strongly curved.

***Wings*.** Forewing length 2.8 mm, 2.95 × longer than wide; wing basally with cells R and 1A largely devoid of setae; 1R1 and 1M with larger, more sparse setae compared to wing apical of these cells, which is evenly covered with smaller, more dense setae; wing clear, with two dark pigmented vertical bands. Basal wing band narrowest dorsally, starting at basal corner of cell 1M and anterior end of cell R, extending ventrally to cover entire cell 2CU and 3A; apical wing band wider, starting at base of pterostigma, and anterior end of 1R1, extending apically to almost cover entire marginal cell 2R1, ventrally to almost cover entire cell 1+2RS, ventrally wider and more diffuse, with infuscate pigmentation extending across cells 2+3M and 3CU, to lower wing margin and extending towards apical margin not quite reaching wing edge; forewing venation with vein Rs apically curving abruptly towards anterior wing margin to form short, truncate marginal cell 2R1; apical segment of vein M long, extending beyond apex of marginal cell, vein M with small white bulla situated almost 3/5 of vein length. Hind wing with apical stub of vein Rs 2/3 of shortest width between the propodeal submedian longitudinal carinae.

***Metasoma*** in dorsal view 1.47 × longer than wide, with seven dorsally visible terga, all smooth and shining except for terga 6 and 7, which are finely shagreened; exposed portion of ovipositor, in lateral view, sub-equal in length to metasomal length; ovipositor sheaths setose, strongly curled (an artefact of preservation).

##### Variation present in paratype females.

Hind leg may be lighter reddish brown in colour than holotype. Body length 2.25–3.8 mm. Forewing length 1.9–2.8 mm.

#### ﻿ Dinapsis centralis

Taxon classificationAnimaliaHymenopteraMegalyridae

Shaw & van Noort, 2009

02DC4DF0-0D6F-5B14-9174-1BA80B466DA8

Figs 6 , 7

##### Material examined.

***Holotype***. Central African Republic • 1 ♀; Prefecture Sangha-Mbaéré, Parc National de Dzanga-Ndoki, 38.6 km. 173˚ S. Lidjombo; 2°21.60'N, 16°03.20'E; 350 m a.s.l.; 22 May 2001; lowland rain forest; S. van Noort leg.; sweeping; CAR01-S230; SAM-HYM-P0024654; SAMC (lost in return postal shipment from USA to SAMC).

##### Other material examined.

Cameroon • 1 ♀; S.W. Province, Korup [5.2014°N, 8.8934°E], Big Rock Camp; 27 Dec. 1980–10 Jan. 1981; D. Jackson leg.; BMNH(E) 2007-19; NHMUK. Kenya • 1 ♀; Coast Province, Mrima Hill Forest; 4.48576°S, 39.25845°E; 212 m a.s.l.; 17–30 Oct. 2011; R. Copeland; Malaise trap; edge of indigenous forest; ICIPE 49120; ICIPE. UGANDA • 1 ♀; District Masindi, Budongo Forest n. Sonso; 1°45'N, 31°35'E; 11–20 Jul. 1995; Th. Wagner leg.; fogging *Tecleanobilis* (Rutaceae); CNC • 1♀, same data except fogging *Rinoreaardisiifolia* (Violaceae); CNC.

##### Diagnosis.

**Figure 6.**
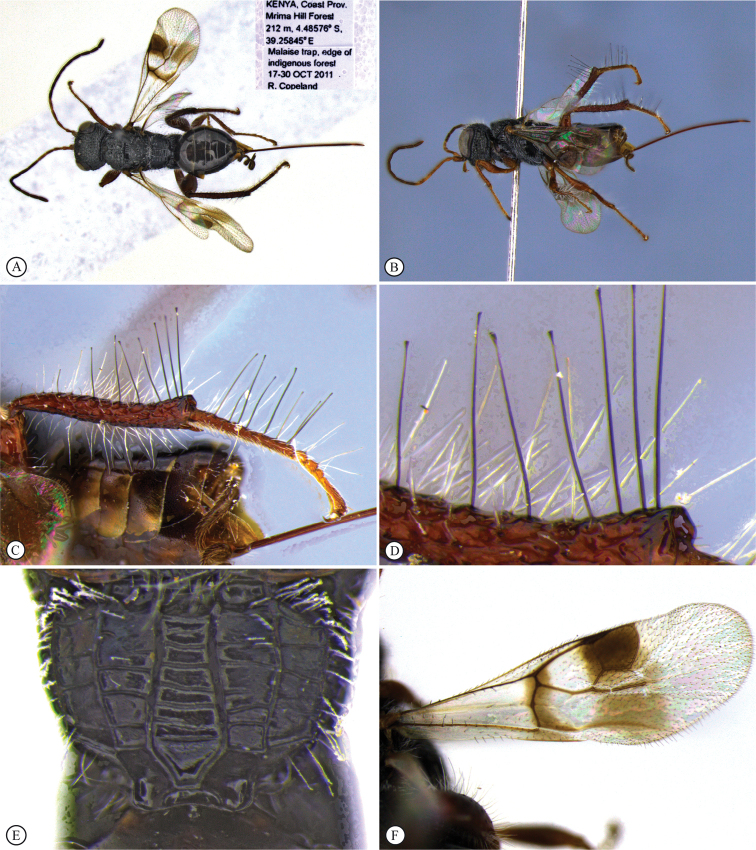
*Dinapsiscentralis* non-type female, ICIPE 49120 (ICIPE) **A** habitus, dorsal view **B** habitus, lateral view **C** hind leg, lateral view **D** spatulate tibial setae, lateral view **E** propodeum, dorsal view **F** forewing.

In the key to African *Dinapsis* species by Hedqvist (1967), *Dinapsiscentralis* keys to couplet 2 because of the presence of minute ocular setae, a characteristic shared with *Dinapsisoculohirta* Hedqvist. *Dinapsisoculohirta* is a smaller species (< 3 mm), with more densely setose eyes, flagellum having a medial pale band, smooth vertex with a medial row of punctures between ocelli, two postocular orbital carinae, smooth mesoscutal lobes, scutoscutellar sulcus demarcated by row of punctures, more pale forewing bands (not filling the marginal cell), and propodeum medially lacking transverse carinae between the submedian longitudinal carinae. In contrast, *D.centralis* is a larger species (> 4 mm) with completely dark flagellum that may be basally lighter but without a medial pale band, coarsely foveate-reticulate vertex, one postocular orbital carina, coarsely foveate-reticulate mesoscutal lobes, transscutal articulation demarcated by sulcus, dark forewing bands completely filling the marginal cell, and propodeum medially with well-developed transverse carinae between the submedian longitudinal carinae. Another curious and distinctive character seen in this species is the presence, on the hind tibia and basitarsus, of large erect dorsal setae, some of which have expanded spatulate tips (Figs 6c, 6D).

##### Distribution.

(Fig. 44) Cameroon, Central African Republic, Kenya, and Uganda.

##### Comments.

This is a rainforest and coastal forest associated species and is expected to be widespread across the central African region. The Cameroon record represents the northernmost confirmed distribution of *Dinapsis* in the Afrotropical region. The Uganda specimens are darker than other specimens, with the legs and metasoma being mostly black rather than dark brown. The Kenyan specimen was collected in Mrima Hill forest at an elevation of 212 m and co-occurs geographically with *D.tricolor*.

**Figure 7.**
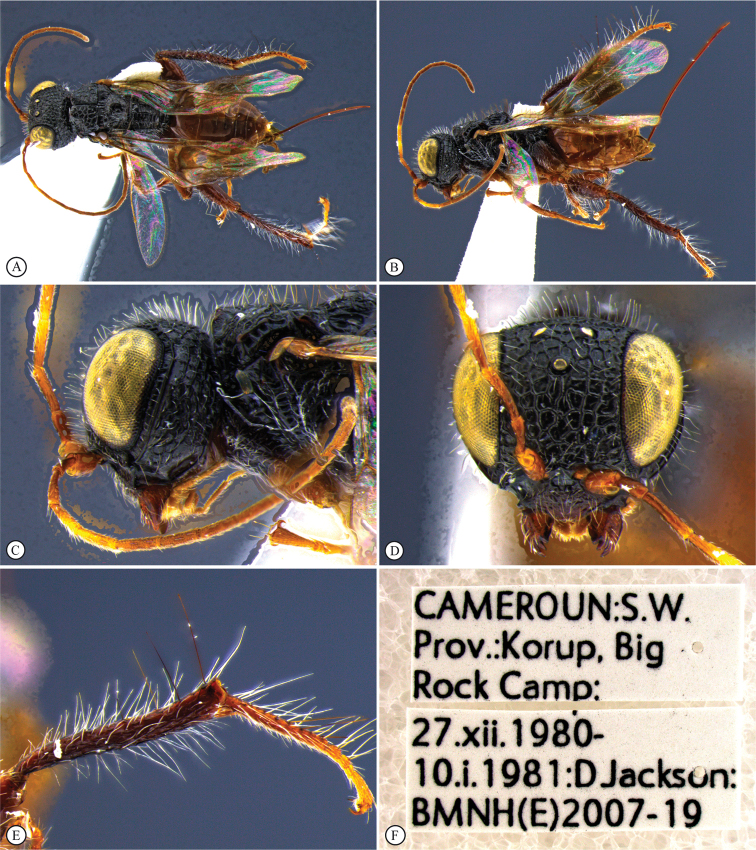
*Dinapsiscentralis* Shaw & van Noort, non-type female BMNH(E) 2007-19 (NHMUK) **A** habitus, dorsal view **B** habitus, lateral view **C** head, mesosoma, lateral view **D** head, anterior view **E** hind leg, anti-axial view **F** data labels.

#### ﻿ Dinapsis cresta

Taxon classificationAnimaliaHymenopteraMegalyridae

Mita & Shaw, 2020

ADBC2139-2FF6-5244-9900-669E99112929

##### Material examined.

***Holotype*.** Madagascar • ♀; Province Diego-Suarez, Parc National Montagne d’Ambre; 12°31'13"S, 49°10'45"E; 1125 m a.s.l.; 4–19 Mar. 2001; R. Harin’Hala leg.; Malaise trap MA-01-01D-05; CASENT2009739; CAS. ***Paratype*** data listed in Mita and Shaw, 2020.

##### Distribution.

(Fig. 43) Madagascar.

##### Comments.

*Dinapsiscresta* is currently known only from the type specimens from three localities in northern and eastern Madagascar. *Dinapsiscresta* is most similar to *D.albicauda*, from which it can be distinguished by its flatter face and entirely brown ovipositor. For a full species description and more information on its distribution see Mita and Shaw (2020).

#### ﻿ Dinapsis gamka

Taxon classificationAnimaliaHymenopteraMegalyridae

van Noort & Shaw sp. nov.

18B21C60-A8EF-504A-AF99-021BAC1F19B3

https://zoobank.org/73C9F9B7-0D7A-4D2E-8345-E192D0ADA178

**Figure 8.**
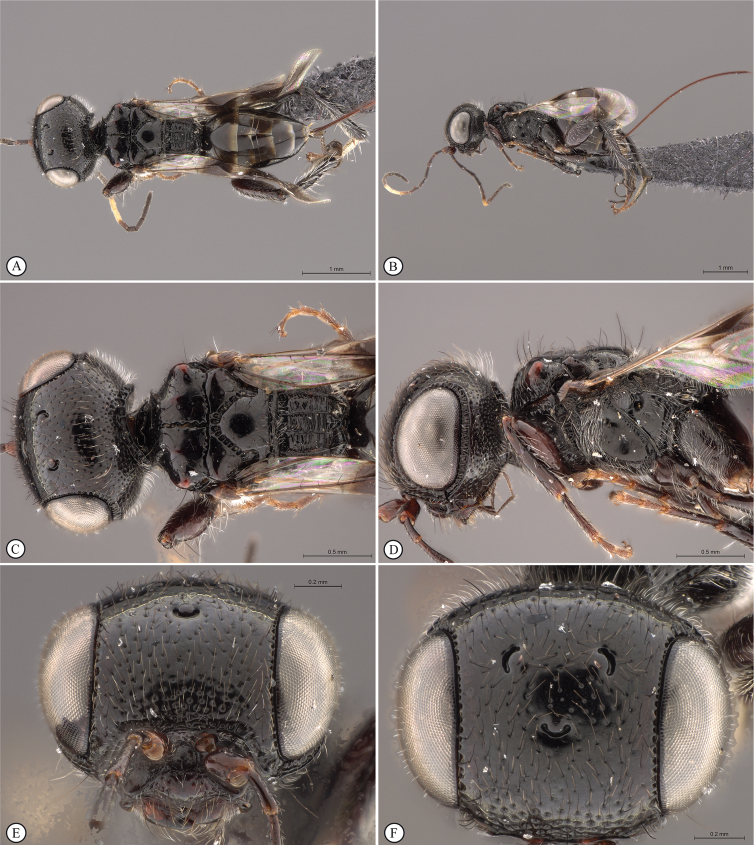
*Dinapsisgamka* van Noort & Shaw, sp. nov. holotype female, SAM-HYM-P048061 (SAMC) **A** habitus, dorsal view **B** habitus, lateral view **C** head, mesosoma, dorsal view **D** head, mesosoma, lateral view **E** head, anterior view **F** head, dorsal view. Scale bars: 1000 µm (**A, B**); 500 µm (**C, D**); 200 µm (**E, F**).

**Figure 9.**
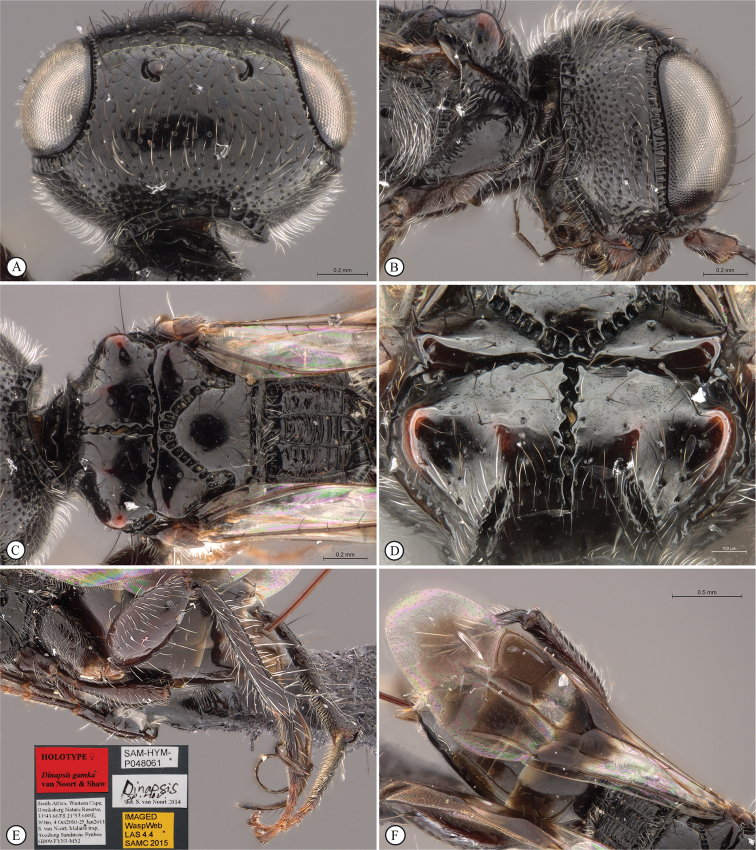
*Dinapsisgamka* van Noort & Shaw, sp. nov. holotype female, SAM-HYM-P048061 (SAMC) **A** head, dorsoposterior view **B** head, lateroposterior view **C** mesosoma, dorsal view **D** mesoscutum, anterodorsal view **E** hindleg, antiaxial view (inset: data labels) **F** forewing, dorsal view. Scale bars: 200 µm (**A-C**); 100 µm (**D**); 500 µm (**F**).

**Figure 10.**
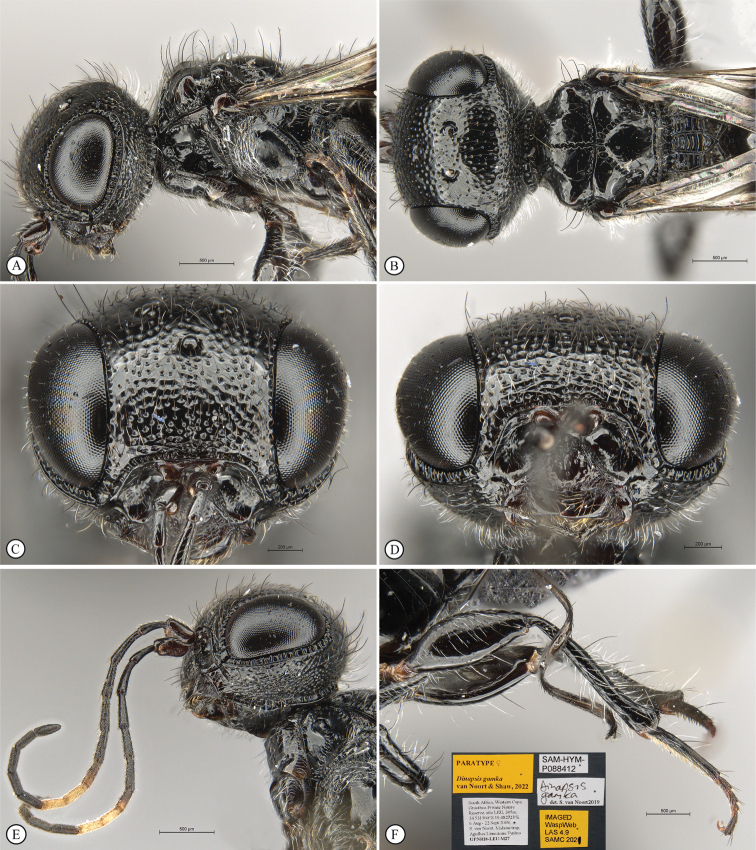
*Dinapsisgamka* van Noort & Shaw, sp. nov. paratype female, Grootbos PNR, SAM-HYM-P088412 (SAMC) **A** head, mesosoma, lateral view **B** head, mesosoma, dorsal view **C** head, anterior view **D** head, ventral view **E** head, antennae, lateral view **F** hind leg antiaxial view (inset: data labels). Scale bars: 500 µm (**A, B, E, F**); 200 µm (**C, D**).

**Figure 11.**
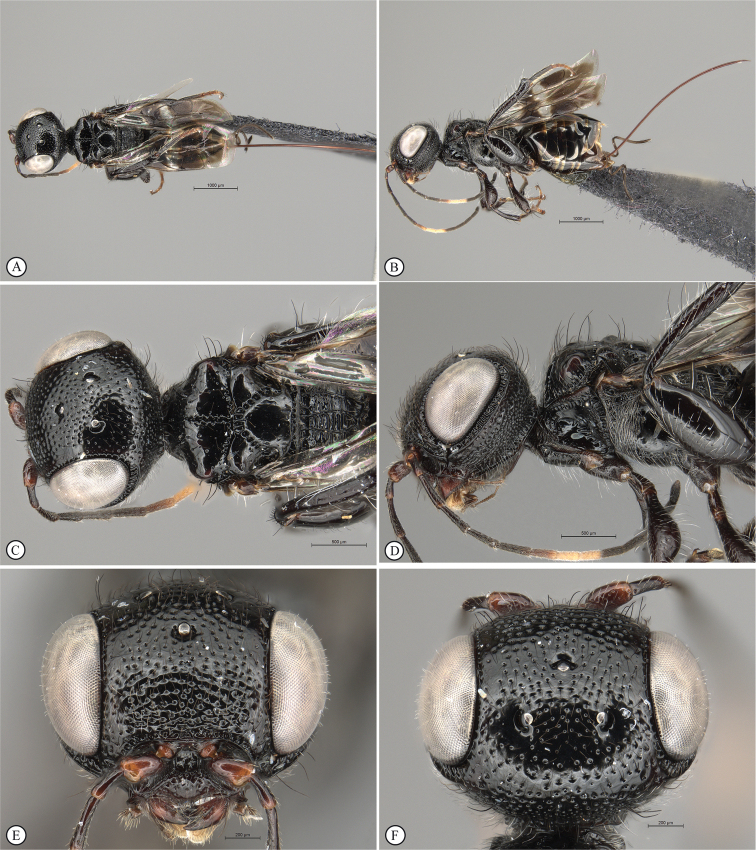
*Dinapsisgamka* van Noort & Shaw, sp. nov. paratype female, Grootvadersbosch NR, SAM-HYM-P043550 (SAMC) **A** habitus, dorsal view **B** habitus, lateral view **C** head, mesosoma, dorsal view **D** head, mesosoma, lateral view **E** head, anterior view **F** head, dorsal view. Scale bars: 1000 µm (**A, B**); 500 µm (**C, D**); 200 µm (**E, F**).

**Figure 12.**
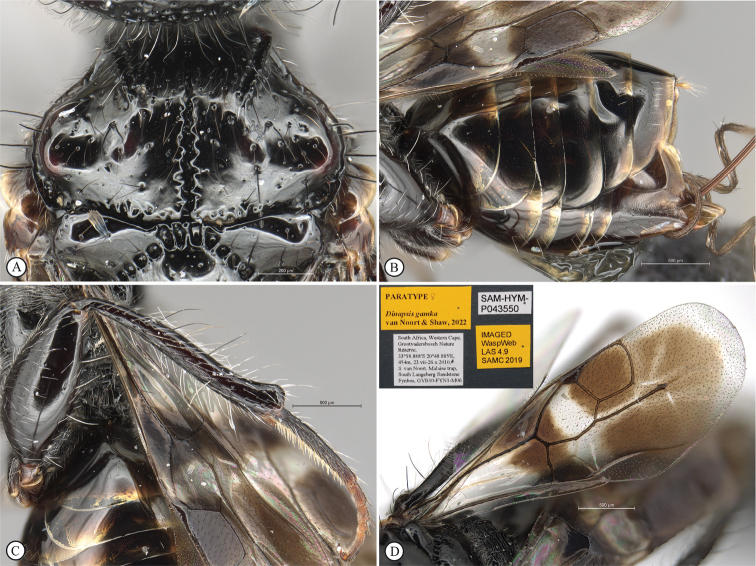
*Dinapsisgamka* van Noort & Shaw, sp. nov. paratype female, Grootvadersbosch NR, SAM-HYM-P043550 (SAMC) **A** mesoscutum, anterodorsal view **B** metasoma, lateral view **C** hindleg, antiaxial view **D** forewing, dorsal view (inset: data labels). Scale bars: 200 µm (**A**); 500 µm (**B, C, D**).

Figs 8 , 9 , 10 , 11 , 12

##### Material examined.

***Holotype*.** South Africa • ♀; Western Cape, Gamkaberg Nature Reserve; 33°43.663'S, 21°57.600'E; 940 m a.s.l.; 4 Oct. 2010–25 Jan. 2011; S. van Noort leg.; Malaise trap; Rooiberg Sandstone Fynbos; GB09-FYN1-M52; SAM-HYM-P048061; SAMC. ***Paratypes*.** South Africa • 1 ♀; Western Cape, Banghoek Valley, Dwarsriviershoek Farm; 33°56.824'S, 18°58.123'E; 400 m a.sl.; 12 Sep. –7 Oct. 2014; S. van Noort leg.; Malaise trap; Mesic Mountain Fynbos; BH12-FYN6-M21; SAM-HYM-P048072; SAMC • 1 ♀; same data except for: 21 Sep. –3 Nov. 2015; BH12-FYN6-M31; SAM-HYM-P086442; SAMC • 1 ♀; Table Mountain National Park, Orangekloof, Disa River; 34°0.035'S, 18°23.492'E; 136 m a.s.l.; 30 Sep. –11 Nov. 2014; S. van Noort leg.; Malaise trap; Afromontane forest; OGK13-FOR1-M26; SAM-HYM-P048018; SAMC • 1 ♀; Grootvadersbosch Nature Reserve; 33°58.888'S, 20°48.885'E; 454 m a.s.l.; 23 Jul.–26 Oct. 2010; S. van Noort leg.; Malaise trap; South Langeberg Sandstone Fynbos; GVB10-FYN1-M06; SAM-HYM-P043550; SAMC • 1 ♀; Grootbos Private Nature Reserve; site LEU; 34.531500°S, 19.482723°E; 305 m a.s.l.; 6 Aug. –22 Sep. 2019; S. van Noort; Malaise trap; Agulhas Limestone Fynbos; GPNR18-LEU-M27; SAM-HYM-P088412; SAMC.

##### Diagnosis.

Morphologically similar to *D.zulu*, both species possessing long mesoscutal setae and the same wing pattern. However, the height of the head in *D.gamka* is distinctly taller than in *D.zulu*, where the head and mesosoma are of nearly equal height (the head of *D.gamka* is 1.5× taller than the mesosoma height). Additionally, the postocular furrow of *D.gamka* is noticeably more narrow than in *D.zulu*. Similar also to *D.taita*, both species having a polished mesoscutum with distinctly raised anterior knobs, but only the very peaks of the knobs may be faintly orange-brown (or may be black as in specimen from Grootbos, which also has faint, very small fovea on part of the mesoscutum) in *D.gamka*, whereas the mesoscutal plate and larger areas of the dorsal surface encompassing the raised knobs are distinctly reddish orange in *D.taita*. Head finely punctate, with moderately long setae, whereas it is distinctly rugulose in *D.taita*. Scutoscutellar sulcus demarcated by broad, foveate furrow, which is very narrow in *D.taita*. Legs and hind coxae black. Hind tibia with long setae. Forewing shiny, sparsely setose, with a characteristic broad, black distal band, centrally extending as infuscation towards the distal wing margins, but not reaching the margin on any side. Hind tibia with long black setae dispersed between smaller white setae.

##### Distribution.

(Fig. 44) South Africa (Western Cape Province).

##### Etymology.

This species is named after the Khoisan word (gamka) for lion. Noun in apposition.

##### Description.

**Holotype female. *Body*** length 4.1 mm excluding ovipositor.

***Colour*.** Head and mesosoma black. Head with a dense covering of white setae on occiput, brown, more widely spaced setae on face and frons; mesoscutal plate black, peaks of anterio-lateral mesoscutal knobs may be faintly orange-brown; metasoma dark brown. Scape, pedicel, F1 to F4 and F8 to F12, fore coxae, trochanters, tibiae dark brown. F5, tarsi, ovipositor orange-brown; mandibles and hind leg reddish brown. F6 and F7 white forming a median anellus on antennae. Eyes and ocelli silvery. Wing membrane clear except for two broad dark brown pigmented bands across forewing with the apical band extending as an infuscation towards, but not reaching apical margin.

***Head*** oval, 1.25 × wider than high; vertex, frons, and face evenly sparsely punctate, interstices polished and 1–3 × greater than puncture width; ocelli small, OOL 3.0 × ocellar diameter; all ocelli bounded by a semi-circular depression on the side facing outer edge of the triangle; ocellar triangle equilateral; eye large and slightly protuberant, nearly parallel in anterior view, but diverging slightly ventrally; eye densely and evenly covered with minute white ocular setae; eye margined posteriorly by foveate groove; postocular orbital carina absent; antenna with 12 flagellomeres having flagellar length/width ratios as follows: F1 = 3.5, F2-F4 = 3.0, F5-F8 and F12 = 2.5, F9-F11 = 2.0; apical flagellomere same width as basal flagellomeres; temple adjacent to ocular orbital carina sparsely punctate and polished, becoming more densely punctate towards occipital carina, temple width 0.5 × eye width in lateral view; malar length 1.3 × mandible width basally; occiput punctate; occipital carina wide and crenulate.

***Mesosoma*.** Pronotum polished, laterally excavated with a row of large oblong foveae situated dorsally and posteriorly on the margin with the mesopleuron; medially with angled, central tri-radiating rows of foveae. Mesoscutal anterior plate polished, with a medial row of punctures, and a lateral carina bounded by a foveate groove; mesoscutum 0.88 × wider than long, mesoscutal lobes polished, with scattered long setae, antero-lateral corners tinged orange, smoothly rounded without tubercles; medial mesoscutal furrow deep and sinusoidally jagged with fovea; transscutal articulation a smooth furrow, anterior edge crenulated, posterior edge smooth; scutoscutellar sulci comprising a line of adjacent large fovea, anteriorly meeting before reaching transscutal articulation; scutellar disc medially polished, with scattered erect, long, brown setae laterally; mesopleuron antero-laterally deeply foveate with short white setae, medially polished with dense patch of long, erect white setae, with large median mid-pit. Metanotum with raised, setose medial area flanked anteriorly by narrow foveate depression laterally grading into wider, larger foveae. Propodeum medially with strongly developed transverse carinae between submedian longitudinal carinae, progressively less transverse carinae present from central to lateral longitudinal tracks. Medial track anteriorly with two deep fovea, lateral tracks each anteriorly with three or four deep foveae.

***Legs*.** Apex of fore tibia with comb of stout spines; hind coxa sparsely punctate, densely covered with long, silky, white setae obscuring surface on ventral 1/2; hind femur stout, polished, 2.14× longer than wide, outer surface of hind femur sparsely, but evenly covered with long, erect, white setae, inner surface of hind femur polished sparsely punctate with very short setae; surface of hind tibia polished, with long erect white setae dorsally, shorter setae laterally and ventrally; dorsal setae lacking spatulate tips; inner ventral margin of hind tibia with a dense longitudinal patch of shorter whitish yellow setae; hind basitarsus long, subequal in length to remaining four tarsomeres combined; basitarsus ventrally with dense preening brush consisting of numerous short, whitish yellow setae, inclined anteriorly; basitarsus dorsally with normal long, white setae, lacking spatulate tips; T2 twice as long as wide, T3 1.8× as long as wide, T4 ca. as long as wide, T5 3 × as long as wide; all tarsomeres with normal hair-like setae; tarsal claw simple, strongly curved.

***Wings*.** Forewing length 2.85 mm, 3.16× longer than wide; wing basally with cells R and 1A largely devoid of setae; 1R1 and 1M with very small, sparse setae compared to wing apical of these cells, which is evenly covered with small, scattered setae; wing clear and overall appearing polished, with two dark pigmented vertical bands. Basal wing band narrowest posteriorly, covering basal 1/3 of cell 1M, anterior 1/3 of cell R, and anterior 2/5 of cell 1Cu, extending posteriorly to wing margin, covering entire cell 2CU and 3A; apical wing band wider, starting at base of pterostigma, and anterior end of 1R1, extending apically to cover entire marginal cell 2R1, posteriorly to cover entire cell 1+2RS, posteriorly wider and more diffuse, with infuscate pigmentation extending across cells 2+3M and 3CU, but not reaching lower wing margin, and extending towards apical margin but not reaching wing edge resulting in a clear semi-circular band parallel to the apical wing margin; forewing venation with vein Rs apically curving abruptly towards anterior wing margin to form short, truncate marginal cell 2R1; apical segment of vein M long, extending beyond apex of marginal cell, vein M with small white bulla situated at 2/3 of vein length. Hind wing with apical stub of vein Rs 2/3 of shortest width between the propodeal submedian longitudinal carinae.

***Metasoma*** in dorsal view 1.67 × longer than wide, with seven dorsally visible terga, all polished; exposed portion of ovipositor, in lateral view 2.0 × longer than metasomal length; ovipositor sheaths setose, strongly curled (an artefact of preservation).

##### Variation.

There is variation in the colouring of the mesoscutum across localities, with the paratype female from Grootbos Nature Reserve having the distinctly raised anterior knobs on the mesoscutum black, and additionally this specimen has faint, very small foveae on part of the mesoscutum, which is usually polished. The Grootvadersbosch specimen has only very faint indications of brownish orange colouration on the knobs. For now, we regard this variation as being intra-specific.

#### ﻿ Dinapsis hirtipes

Taxon classificationAnimaliaHymenopteraMegalyridae

Hedqvist, 1967

5DC60AF5-90D6-5C83-AF7A-48E112A2798A

Fig. 13

##### Material examined.

***Holotype*.** Madagascar • ♀; Ivondro; [18.238933°S, 49.366374°E]; Museum Paris; Apr. 1941; A. Seyrig [blue labels]; Coll. Mus. Tervuren [white label]; HOLOTYPUS *Dinapsishirtipes* sp. n. ♀ K-J Hedqvist det. 1967 [red label]; MNHN.

##### Distribution.

(Fig. 43) Madagascar.

##### Comments.

**Figure 13.**
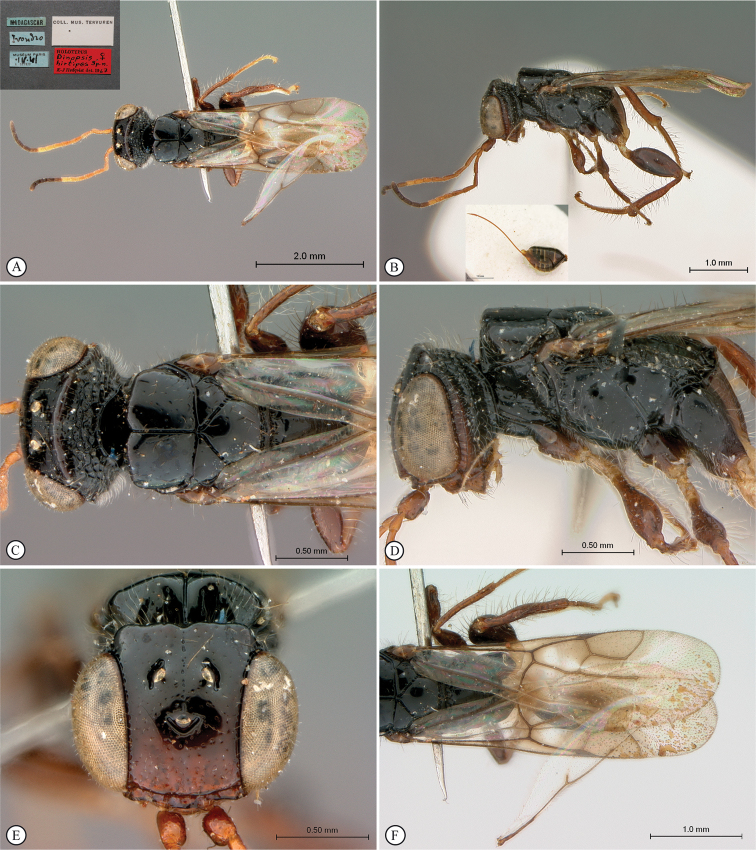
*Dinapsishirtipes* holotype female (MNHN) **A** habitus, dorsal view (inset: data labels) **B** habitus, lateral view (inset: metasoma lateral view) **C** head, mesosoma, dorsal view **D** head, mesosoma, lateral view **E** head, anterior view **F** wings, dorsal view. Scale bars: 2000 µm (**A**); 1000 µm (**B, F**); 500 µm (**C, D, E**).

*Dinapsishirtipes* is known only from the type- locality at Ivondro, Madagascar. Ivondro is located in the Atsimo-Atsinanana region. *Dinapsishirtipes* is a distinctive species with a raised (crested) vertex (Fig. 13C, E) and banded wings (Fig. 13F). When it was described by Hedqvist (1967) it was the only known megalyrid with this distinctive crested head shape. Mita and Shaw (2020) assigned it to the *hirtipes* species group with five other new species described in their revision, all of which can be distinguished using the key in this paper, or the key in Mita and Shaw (2020). For a full species description and more information on its distribution and distinguishing characteristics, see Mita and Shaw (2020).

#### ﻿ Dinapsis igneus

Taxon classificationAnimaliaHymenopteraMegalyridae

van Noort & Shaw sp. nov.

3176FD4D-B936-5597-82E1-36BBBD5D8A1D

https://zoobank.org/742EB1A2-DF10-478F-8DA6-5F25949EF6E0

**Figure 14.**
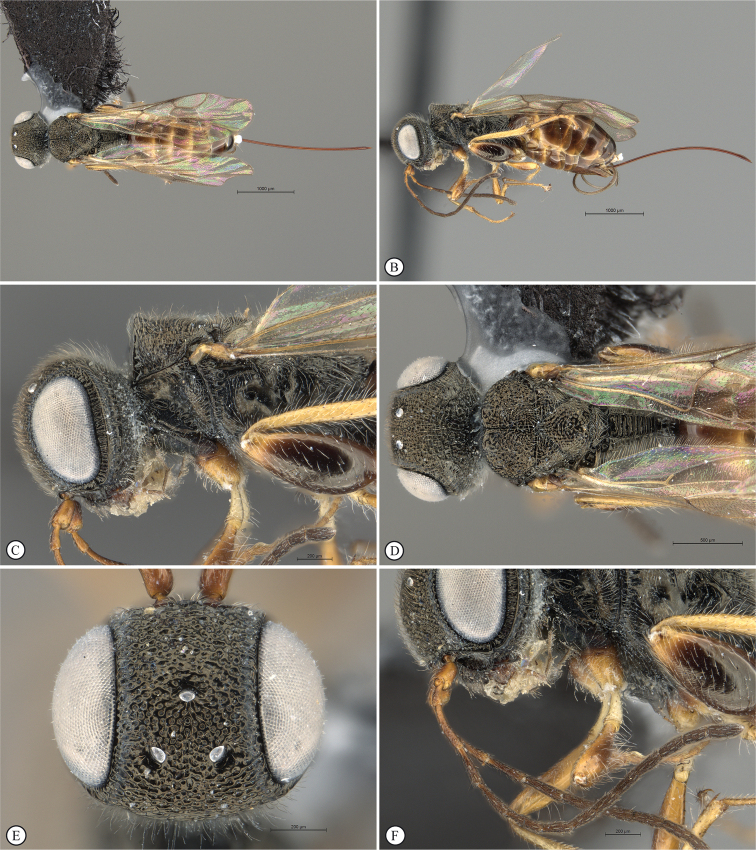
*Dinapsisigneus* van Noort & Shaw, sp. nov. holotype female (DEBU) **A** habitus, dorsal view **B** habitus, lateral view **C** head, mesosoma, lateral view **D** head, mesosoma, dorsal view **E** head, dorsal view **F** antennae, lateral view. Scale bars: 1000 µm (**A, B**); 200 µm (**C, E, F**); 500 µm (**D**).

**Figure 15.**
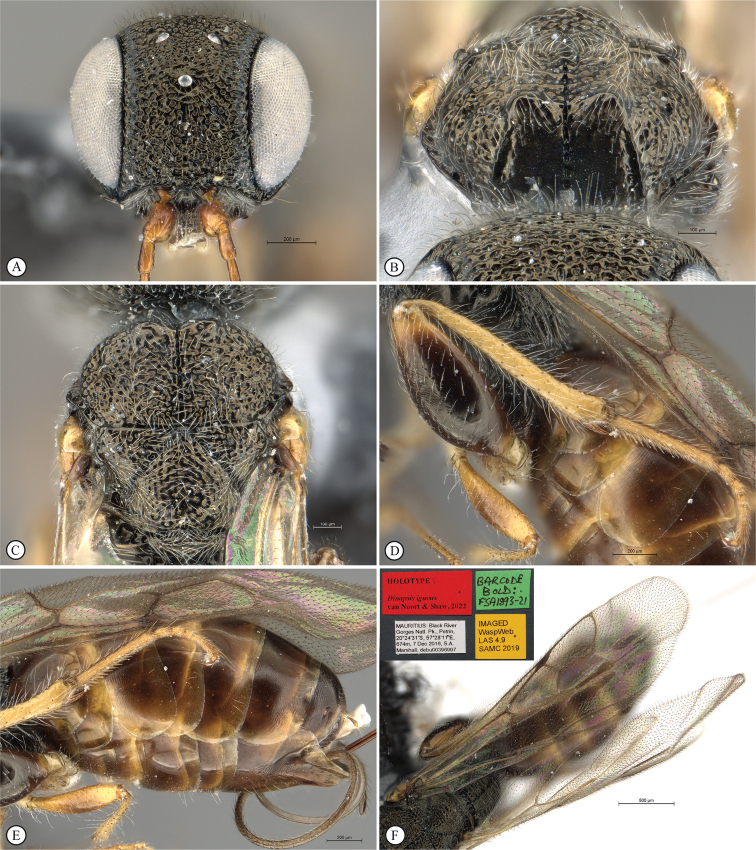
*Dinapsisigneus* van Noort & Shaw, sp. nov. holotype female (DEBU) **A** head, anterior view **B** mesoscutum, anterior view **C** mesosoma, dorsal view **D** hind leg, anti-axial view **E** metasoma, lateral view **F** wings, dorsal view (inset: data labels). Scale bars: 200 µm (**A, D, E**); 100 µm (**B, C**); 500 µm (**F**).

**Figure 16.**
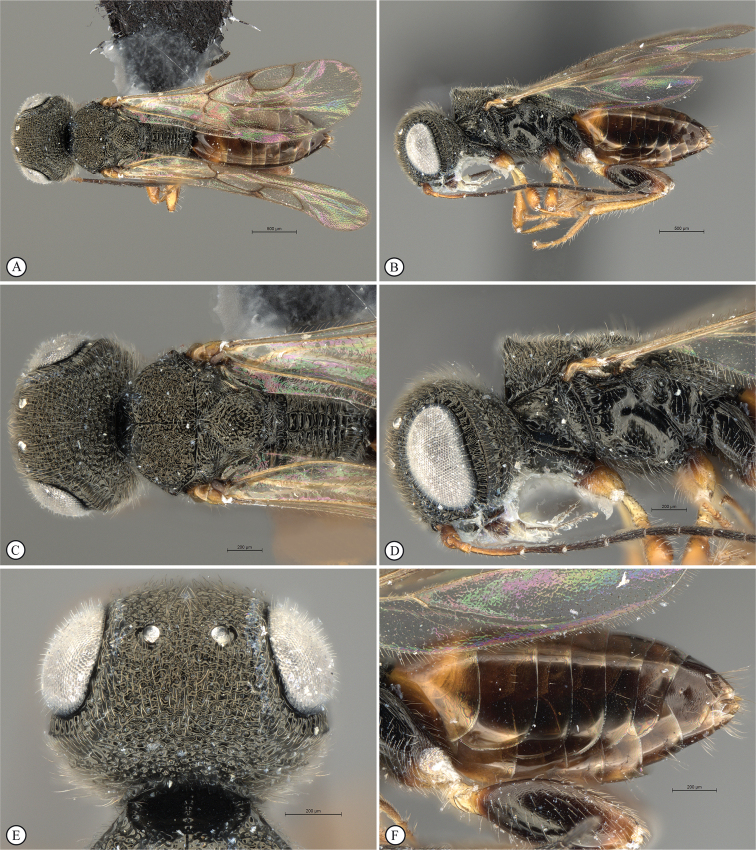
*Dinapsisigneus* van Noort & Shaw, sp. nov. paratype male (DEBU) **A** habitus, dorsal view **B** habitus, lateral view **C** head, mesosoma, dorsal view **D** head, mesosoma, lateral view **E** head, dorsoposterior view **F** metasoma, lateral view. Scale bars: 500 µm (**A, B**); 200 µm (**C, D, E, F**).

**Figure 17.**
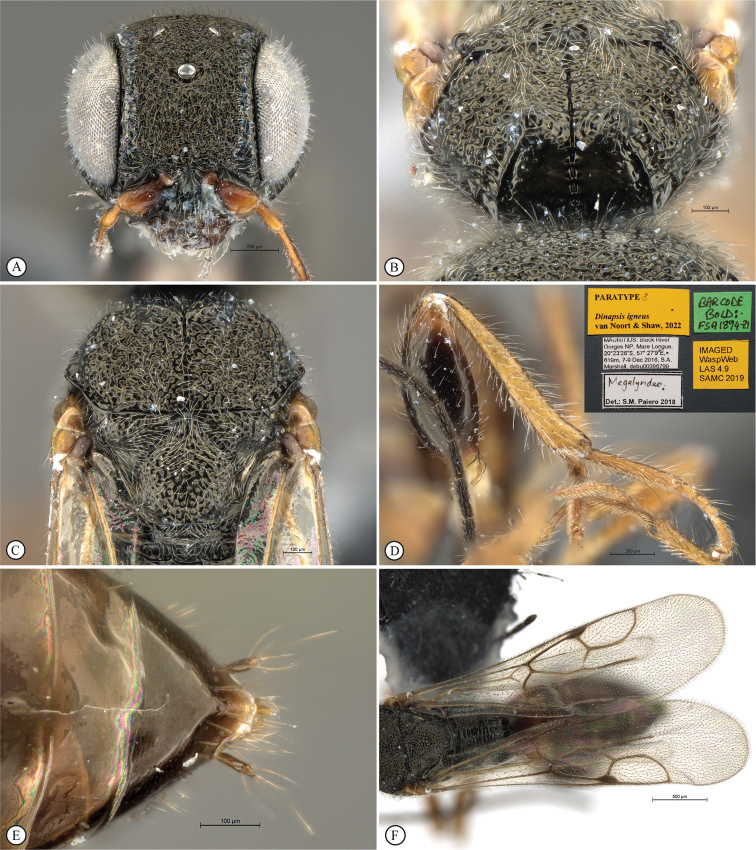
*Dinapsisigneus* van Noort & Shaw, sp. nov. paratype male (DEBU) **A** head, anterior view **B** mesoscutum, anterodorsal view **C** mesosoma, dorsal view **D** hind leg, antiaxial view (inset: data labels) **E** metasoma terminal tergites and pygostyle, dorsal view **F** wings, dorsal view. Scale bars: 200 µm (**A, D**); 100 µm (**B, C, E**); 500 µm (**F**).

Figs 14 , 15 , 16 , 17

##### Material examined.

***Holotype*.** Mauritius • ♀; Black River Gorges Natl. Pk., Pétrin; 20°24'31"S, 57°28'11"E; 674 m a.s.l.; 7 Dec. 2016; S.A. Marshall leg.; debu00396997; BARCODE BOLD:FSA1893-21; IMAGED WaspWeb LAS 4.9 SAMC 2019; DEBU. ***Paratypes*.** Mauritius • 1 ♂; Black River Gorges N.P.; Mare Longue, 20°23'26"S, 57°27'9"E; 619 m a.s.l.; 7–9 Dec. 2016; S.A. Marshall leg.;, debu00396799; Megalyridae Det.: S.M. Paiero 2018; BARCODE BOLD:BOLD:FSA1894-21; IMAGED WaspWeb LAS 4.9 SAMC 2019; DEBU • 1 ♀; Black River, Black River Gorges N.P., Pétrin; 20°24'31"S, 57°28'11"E; 24 Jan.–2 Feb. 2018; 620 m a.s.l.; Kirk-Spriggs & Muller leg.; Malaise trap; upland heath forest; ICIPE • 1 ♂; Black River, Black River Gorges N.P., Mare Longue; 20°23'26"S, 57°27'09"E; 24 Jan. –2 Feb. 2018; 619 m a.sl.; Kirk-Spriggs & Muller leg.; Malaise trap; Montane rainforest; ICIPE.

##### Diagnosis.

*Dinapsisigneus* can be assigned to the *oculohirta* species group by the presence of dense ocular setae on the eyes. It can be distinguished from *D.oculohirta*, and others in that group, by being the only *Dinapsis* species with a slight metallic sheen to the integument (as seen under a microscope with illumination). The mesoscutum has a characteristic strongly raised bilobed crest situated anteriorly (Fig. 15B); the head and mesosoma has a characteristic weakly metallic greenish bronze sheen (only observable under sufficient illumination) and is densely rugulose-punctate and covered with dense white setae (Fig. 16E). The latter character states are also not present in any other species, which are non-metallic, black, or brown, or rarely with orange patches, have a mesosoma which is often smooth, sometimes with scattered foveae, if sculptured then the integument is broadly foveate, and the mesosoma is often semi-glabrous, and only ever sparsely covered with setae.

##### Distribution.

(Fig. 43) Mauritius.

##### Comments.

Known only from Black River Gorges National Park in both the montane rainforest and upland heath forest habitat. This is the first megalyrid species to be described from an island that is volcanic in origin, and not a more ancient fragment of continental rock. Previous authors have commented on the association of Megalyridae with ancient continental landmasses (Shaw 1990b; Vilhelmsen et al. 2010). This raises interesting questions about whether this species is derived from megalyrid ancestors that dispersed naturally to the island in ancient times (perhaps by drifting in wood with infected host insects), or whether the species might have been accidentally transported to the island by human commerce, either from Madagascar or the African mainland, in the last 500 years. We expect it will be found from Africa or Madagascar eventually, unless it is extirpated in those places.

##### Etymology.

This species is named after the Latin for fiery (*igneus*) in reference to the provenance of the species from Mauritius, which is a volcanic island formed ca. 8 million years ago. The species epithet is to be treated as an adjective.

##### Barcode sequence for holotype female.

38754_A01_Din_ign_fem (sequence code in BOLD: FSA1893-21) BIN URI: None (sequence too short).

##### Nucleotide sequence for holotype female.

CTATAAGAATAATTATTCGTATGGAACTTAGAGTTCCGGGTTCATTTATTGGAAATGATCAGATTTATAATTCTATTGTGACTGCACATGCTTTTATTATAATTTTTTTTATAGTAATACCTTTTATAATGGGAGGTTTTGGTAATTGGTTATTGCCCTTAATGTTAGGGGCTCCTGATATGTCTTACCCTCGTCTTAATAATTTAAGGTTTTGATTGTTGGTTCCTTCTTTGTTATTTTTATTAATA.

##### Barcode sequence for paratype male.

38754_A02_Din_ign_mal (sequence code in BOLD: FSA1894-21) BIN URI: None (sequence too short).

##### Nucleotide sequence for paratype male.

TTGAGCAGGGCTTATTGGATCATCTATAAGAATAATTATTCGTATGGAACTTAGAGTTCCGGGGTCATTTATTGGGAATGATCAGATTTATAATTCTATTGTAACTGCACATGCTTTTATTATAATTTTTTTTATAGTAATACCTTTTATAATGGGAGGGTTTGGTAATTGATTATTGCCTTTGATGTTAGGGGCCCCTGATATGTCTTATCCTCGTCTTAATAATTTAAGGTTTTGATTATTGGTTCCTTCTTTGTTATTTTTACTAATAAGGTTTTATGTGGGCAGAGGTACAGGAA.

##### Description.

**Holotype female. *Body*** length 4.0 mm excluding ovipositor.

***Colour*.** Head and mesosoma with greenish bronze sheen. Head with a dense covering of white setae on occiput, darker, more widely spaced setae on face and frons; mesoscutal plate with a greenish bronze sheen. Mesoscutal plate with anteriodorsal bilateral peaks. Anteriolateral mesoscutal knobs absent; metasoma brown, lighter yellowish brown along posterior tergal margins. Scape, pedicel, F1, fore coxae and mid coxae, tibiae and tarsi yellowish brown. F2 and F3 light brown; F4 to F12 dark brown with light offset rows of multiporous plate sensilla; trochanters whitish yellow. Hind femur dark brown, except for light yellowish brown apex. Ovipositor orange-brown. Eyes and ocelli silvery. Wing membrane clear without dark bands.

***Head*** round, only slightly (1.13 ×) wider than high; vertex, frons, and face evenly strongly punctate, interstices polished, 1–2 × greater than puncture width; ocelli small, OOL 2.0 × ocellar diameter; all ocelli bounded by a semi-circular depression on the side facing outer edge of the triangle; ocellar triangle isosceles (POL:LOL - 3:4); eye large and hardly protuberant, not parallel in anterior view, strongly diverging dorsally and ventrally; eye densely and evenly covered with minute white ocular setae; eye margined posteriorly by foveate groove; postocular orbital carina weakly present; antenna with 12 flagellomeres having flagellar length/width ratios as follows: F1 = 5, F2-F4 = 4.0, F5-F9 = 2.5, F10-F11 = 2.2 F12 = 2.75; apical flagellomeres distinctly wider than basal flagellomeres; temple adjacent to ocular orbital carina coarsely rugulose, temple width 0.67× eye width in lateral view; malar length equivalent to mandible width basally; occiput coarsely rugulose; occipital carina wide laterally, narrower dorsally and crenulate.

***Mesosoma*.** Pronotum polished, laterally excavated with a row of large quadrate to oblong foveae situated posteriorly on the margin with the mesopleuron; foveae along dorsolateral margin faint and shallow; medially with angled central row of six foveae, dorsal fovea largest, 3 × length of others. Mesoscutal anterior plate polished, with a medial suture grading ventrally into a row of approximately five foveae, and lateral carinae bounded by weak foveae; glabrous except for dorsal fifth which is setose as in rest of mesoscutum; mesoscutum 1.1 × wider than long, shoulders rounded, mesoscutal lobes absent; medially mesoscutum punctate; medial mesoscutal furrow deep, narrow with weakly jagged edge; transscutal articulation a smooth, narrow furrow, anterior edge weakly jagged, posterior edge straight; scutoscutellar sulci medially comprising a continuous shallow groove with defined foveae; anteriorly meeting before reaching transscutellar articulation; scutellar disc punctate; mesonotum dorsally covered with dense white setae; mesopleuron antero-laterally shallowly foveate, dorsal fovea elongate, extending entire dorsal length of mesopleuron, with short white setae except for medially polished area surrounding large median mid-pit. Metanotum with raised, setose (long white setae as on mesoscutum) medial area flanked laterally by depression with 3–5 foveae. Propodeum medially polished with strong transverse carinae between the submedian longitudinal carinae defining the three central tracks; lateral longitudinal tracks with defined transverse carinae. All five tracks anteriorly with two or three deep foveae.

***Legs*.** All legs with long, white setae, each seated in a darker basal socket, contrasting with surrounding pale integument creating weakly spotted appearance. Apex of fore tibia with comb of stout spines; hind coxa polished, with sparse, shorter, white setae; hind femur stout, polished, 2.3 × longer than wide, outer surface of hind femur sparsely covered with short, white setae; inner surface of hind femur polished with very short setae; surface of hind tibia polished, with long, erect white setae dorsally and ventrally, shorter setae laterally; dorsal setae lacking spatulate tips; inner ventral margin of hind tibia with a dense longitudinal patch of shorter white setae; hind basitarsus long, 1.5 × length of remaining four tarsomeres combined; basitarsus ventrally with dense preening brush consisting of numerous short, brown setae, inclined posteriorly; basitarsus dorsally with normal long, white setae, lacking spatulate tips; T2 twice as long as wide, T3 1.3 × longer than wide, T4 ca. as long as wide, T5 ca. 4 × as long as wide; all tarsomeres with normal hair-like setae, but also with scattered elongate, stronger setae projecting from dorsal surface; tarsal claw simple, strongly curved.

***Wings*.** Forewing length 3.1 mm, 3 × longer than wide; wing surface evenly covered with small, scattered setae, including basal cells R and 1A; wing clear, without dark vertical bands. Forewing venation with vein Rs apically curving abruptly towards anterior wing margin to form short, truncate marginal cell 2R1; apical segment of vein M abbreviated, not extending beyond apex of marginal cell, vein M with small white bulla situated at a quarter of vein length. Hind wing with apical stub of vein Rs 2/3 of shortest width between the propodeal submedian longitudinal carinae.

***Metasoma*** in lateral view 1.75 × as long as wide, with seven dorsally visible terga, all polished; exposed portion of ovipositor relatively short, in lateral view 0.76 × metasomal length; dorsal valve with 14 serrations, ventral valve smooth; ovipositor sheaths setose, strongly curled (an artefact of preservation).

##### Variation.

Paratype male has body length 3.75 mm, and forewing length 3.0 mm.

#### ﻿ Dinapsis luteus

Taxon classificationAnimaliaHymenopteraMegalyridae

Mita & Shaw, 2020

25F053AF-6D85-5080-906B-0227D1659A15

##### Material examined.

***Holotype***. Madagascar • ♀; Toliara Prov., Parc National de Zombitse; 19.8 km 84°E Sakaraha; 770 m a.sl.; 5–9 Feb. 2003; 22°50'36"S, 044°42'36"E; Fisher, Griswold et al.; California Acad. of Sciences; yellow pan trap, tropical dry forest; coll. code: BLF7505; CASENT2053467; CAS. ***Paratype*** data listed in Mita and Shaw, 2020.

##### Distribution.

(Fig. 43) Madagascar.

##### Comments.

*Dinapsisluteus* is known from localities in southern and eastern Madagascar (Antananarivo, Fianarantsoa, Toamasina, and Toliara provinces). *Dinapsisluteus* is similar to *D.hirtipes* but can be distinguished by the absence of dark wing bands on the forewings, and instead having the wings entirely light yellow-coloured. For a full species description and more information on its distribution and distinguishing characteristics, see Mita and Shaw (2020).

#### ﻿ Dinapsis nubilis

Taxon classificationAnimaliaHymenopteraMegalyridae

Hedqvist, 1967

BDE78B65-057B-5128-A076-FB6EA96AACCE

Fig. 18

##### Material examined.

***Holotype***. Madagascar • ♀; Ranomafana [21.259457°S, 47.454986°E]; Museum Paris; Aug. 1940; A. Seyrig [blue labels]; Coll. Mus. Tervuren [white label]; HOLOTYPUS *Dinapsisnubilis* sp. n. ♀ K-J Hedqvist det. 1967 [red label]; MNHN.

**Figure 18.**
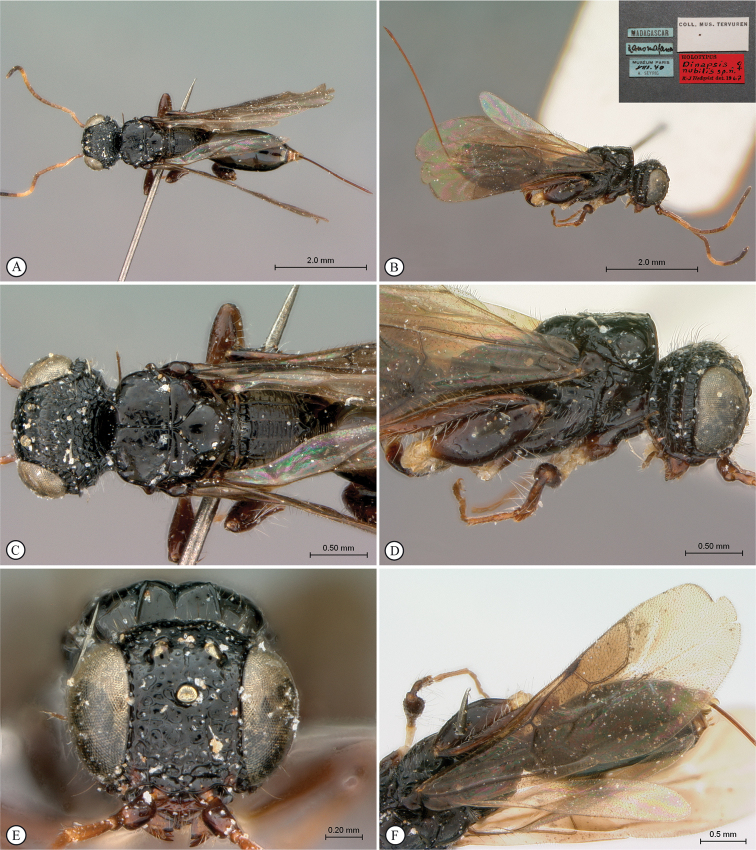
*Dinapsisnubilis* holotype female (MNHN) **A** habitus, dorsal view **B** habitus, lateral view **C** head, mesosoma, dorsal view **D** head, mesosoma, lateral view **E** head, anterior view **F** wings, dorsal view. Scale bars: 2000 µm (**A, B**); 500 µm (**C, D, F**); 200 µm (**E**).

##### Distribution.

(Fig. 43) Madagascar.

##### Comments.

*Dinapsisnubilis* is only known from the type locality of Ranomafana, Madagascar. This small town lies adjacent to the Ranomafana National Park, which is in the south-eastern region of Madagascar in Haute Matsiatra and Vatovavy. *Dinapsisnubilis* is most closely related to *D.albicoxa*, which both share the unusual characteristic of having lobe-like extensions off the sides of the mesoscutum (see discussion in species-group section). *Dinapsisnubilis* can be distinguished from *D.albicoxa* (which has banded wings) by the wings being entirely dark-coloured (Fig. 18F).

#### ﻿ Dinapsis oculohirta

Taxon classificationAnimaliaHymenopteraMegalyridae

Hedqvist, 1967

745A3513-B6F2-5CF2-86C6-14B6F95750A5

Fig. 19

##### Material examined.

***Holotype*.** Madagascar • ♀; Esira [24.325870°S, 46.708602°E]; Museum Paris; Sep. 1940; A. Seyrig leg. [blue labels]; Coll. Mus. Tervuren [white label]; HOLOTYPUS *Dinapsisoculohirta* sp. n. ♀ K-J Hedqvist det. 1967 [red label]; MNHN.

##### Additional specimens examined.

Madagascar • 1 ♀; Toliara Province, Forêt de Kirindy, 15.5 km 64˚ENE Marofandilia, 26 November–3 December 2008; 20°04'07"S, 044°39'34"E, California Acad. of Sciences, B.L. Fisher leg., Malaise trap; tropical dry forest, 30 m a.s.l.; BLF-18284-56; CASENT2237108; CAS • 1 ♀; Tulear Province, Beroboka village, 45 km NE Morandava; 430 ft a.s.l.; 22–30 May 2008; 19°58.65'S, 44°39.92'E; California Acad. of Sciences; M. Irwin, R. Harin’Hala leg.;, Antsarongaza dry forest; Malaise; MG-45A-24; CASENT2187500CAS • 1 ♂; same data except Antsarongaza gallery forest; MG-45B-26; CASENT2187503CAS • 1 ♀; Tulear Province, Androimpano Forest, 5 km E of Itampolo; 11–18 Dec. 2009; 24°39.02'S, 43°57.79'E; M. Irwin, R. Harin’Hala leg.; Malaise; path in dry forest; 130 m a.s.l.; MG-54C-53; CASENT2187501CAS • 1♂; Tulear Province, Beza Mahafaly Reserve, Parcelle 1 near research station; 10–29 Apr. 2002; 23°41.19'S, 44°35.46'E; M.E. Irwin, F.D. Parker, R. Harin’Hala leg.; Malaise trap in dry deciduous forest; 165 m a.s.l.; MA-02-14A-23; CASENT2187502CAS • 1 ♀; same data except 9–20 Sep. 2002; MA-02-14A-35; CASENT2118387CAS • 1 ♂; same data except 1–8 Feb. 2002; MA-02-14B-14; CASENT2118380CAS • 1 ♀; Tulear Province, Berenty Special Reserve, 85 m a.s.l.; 8 km NW Amboasary; 25°00.40'S, 46°18.20'E; 26 Jan.–5 Feb. 2003; M.E. Irwin, F.D. Parker, R. Harin’Hala leg.; Malaise trap; gallery forest; MA-02-22-14; CASLOT 016980; SEM CAS • 1 ♀; same data except 24 Mar.–3 Apr. 2003; MA-02-22-20; CASENT2118383CAS • 1 ♂; same data except 27 Dec. 2002–7 Jan. 2003; MA-02-22-11; CASENT2118382CAS • 1 ♀; same data except 22–30 Nov. 2003; spiny forest; MA-02-22A-04; CASENT2118384CAS.

##### Distribution.

(Fig. 43) Madagascar.

##### Comments.

**Figure 19.**
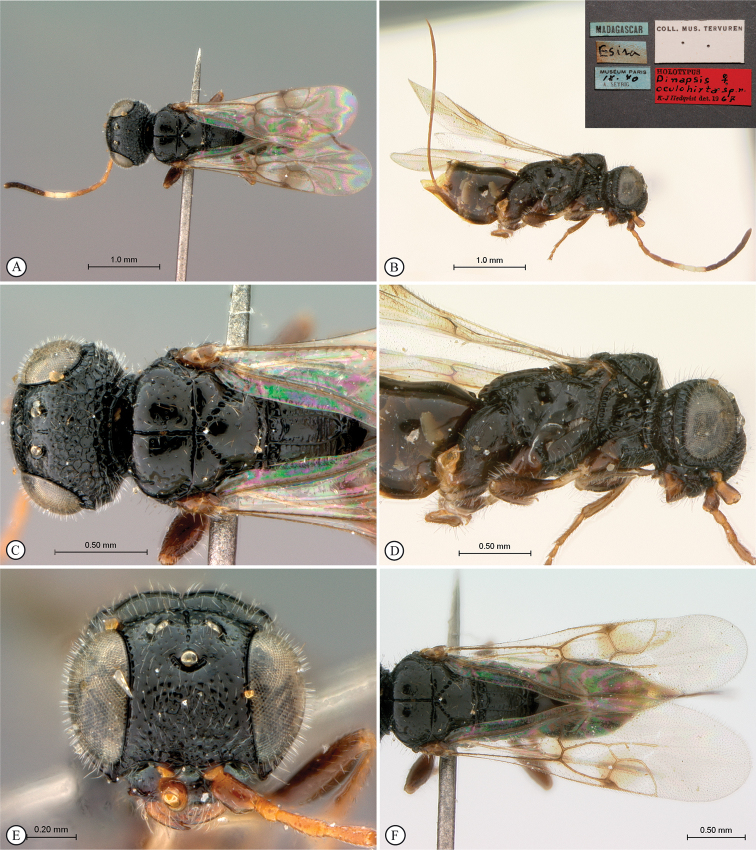
*Dinapsisoculohirta* holotype female (MNHN) **A** habitus, dorsal view **B** habitus, lateral view **C** head, mesosoma, dorsal view **D** head, mesosoma, lateral view **E** head, anterior view **F** wings, dorsal view. Scale bars: 1000 µm (**A, B**); 500 µm (**C, D, F**); 200 µm (**E**).

*Dinapsisoculohirta* is known only from southern Madagascar (Anosy and Toliara [= Tulear] provinces). *Dinapsisoculohirta* was named for the conspicuous dense setae that cover the compound eyes (Fig. 19C, E). It can be distinguished from other members of the *oculohirta* species group by the presence of a small median groove between the ocelli (Fig. 19E). See also couplet 6 of the key to species, and the *oculohirta* species-group discussion in the species-group section of this paper.

#### ﻿ Dinapsis planifrons

Taxon classificationAnimaliaHymenopteraMegalyridae

Mita & Shaw, 2020

DDF77DCA-DDD4-51A1-84D8-785B529EAFE1

##### Material examined.

***Holotype*.** Madagascar • ♀; Antsiranana; Ambato forest, 26.6 km 33°, NE Ambanja; 150 m; 8 December 2004; 13°27'52"S, 048°33'06"E; California Acad. of Sciences; B.L. Fisher; rainforest; yellow pan trap; BLF11517; CASLOT 014881; SEM CAS. ***Paratype*** data listed in Mita and Shaw, 2020.

##### Additional specimens examined.

Madagascar • 3 ♀♀, 1 ♂, Antisirana Province, Makirovana Forest; 225 m a.s.l.; 28 Apr.–8 May 2011; 14°10'14"S, 049°57'15"E; California Acad. of Sciences; B.L. Fisher; rainforest; yellow pan trap; BLF26522; BLF27041; CASENT2237138, CASENT2237139, CASENT2237145, CASENT2237146; CAS • 1 ♂, Antisirana Province, SAVA Region, Vohemar District, Antsahabelela rain forest, 9 km SW Daraina Binara; 16–23 Feb. 2011; 13°15.03'S, 049°37.00'E; California Acad. of Sciences; M. Irwin, R. Harin’Hala leg.; Malaise; in humid forest; MG-58-20; CASENT2237021; CAS.

##### Distribution.

(Fig. 43) Madagascar.

##### Comments.

*Dinapsisplanifrons* is known only from forests in western and northern Madagascar (Antisirana, Fianarantsoa, Mahajanga, and Toliara provinces). *Dinapsisplanifrons* is a very distinctive species, not likely to be confused with any other. It is the most common species of the *Dinapsishirtipes* species group, known as “crested wasps” because of the smooth flat-faced head with the vertex conspicuously produced dorsally as a crested ridge. *D.planifrons* can be distinguished from all other species by having the hind tibia apex produced into an extended prong with long apical seta with spatulate tips. For a full species description and more information on its distribution and distinguishing characteristics, see Mita and Shaw (2020).

#### ﻿ Dinapsis scriptus

Taxon classificationAnimaliaHymenopteraMegalyridae

Mita & Shaw, 2020

3BA575E6-52DD-5F24-B572-434C073A1F3A

##### Material examined.

***Holotype*.** Madagascar • ♀; Province Fianarantsoa, near Isalo National Park, at stream, east of Interpretive Center; 17–24 Mar. 2004; 22°37.60'S, 45°21.49'E; 750 m a.s.l.; R. Harin’Hala leg.; Malaise trap in open area; MA-02-11A-16; CASLOT 016972; CAS. ***Paratype*** data listed in Mita and Shaw, 2020.

##### Distribution.

(Fig. 43) Madagascar.

##### Comments.

*Dinapsisscriptus* is known from localities in northern, central, and southwestern Madagascar (Antsiranana, Fianarantsoa, and Toliara provinces). *Dinapsisscriptus* is distinguished from the other *Dinapsis* species by the hind tibia having a well-developed distal prong with only simple setae (see couplet 14 of the key to species). A similar prong is also present on the hind tibia of *D.planifrons* but that species has spatulate setae on the distal prong. For a full species description and more information on its distribution and distinguishing characteristics, see Mita and Shaw (2020).

#### ﻿ Dinapsis seyrigi

Taxon classificationAnimaliaHymenopteraMegalyridae

Hedqvist, 1967

D7FB7116-4EDC-54A7-BD0A-51020217CD94

Fig. 20

##### Material examined.

***Holotype*.** Madagascar • ♀; Trafonomby [24.549938°S, 46.725393°E]; Museum Paris; Aug. 1940; A. Seyrig leg. [blue labels]; Coll. Mus. Tervuren [white label]; HOLOTYPUS *Dinapsisseyrigi* sp. n. ♀ K-J Hedqvist det. 1967 [red label]; MNHN.

##### Additional specimens examined.

Madagascar • 1 ♀; Fianarantsoa Province, 7 km W Ranomafana; [21.263064°S, 47.383935°E]; 900 m a.s.l.; 20–31 Jan. 1990; W.E. Steiner leg.; Malaise trap in small clearing; montane rain forest; USNM.

##### Distribution.

(Fig. 43) Madagascar.

##### Comments.

**Figure 20.**
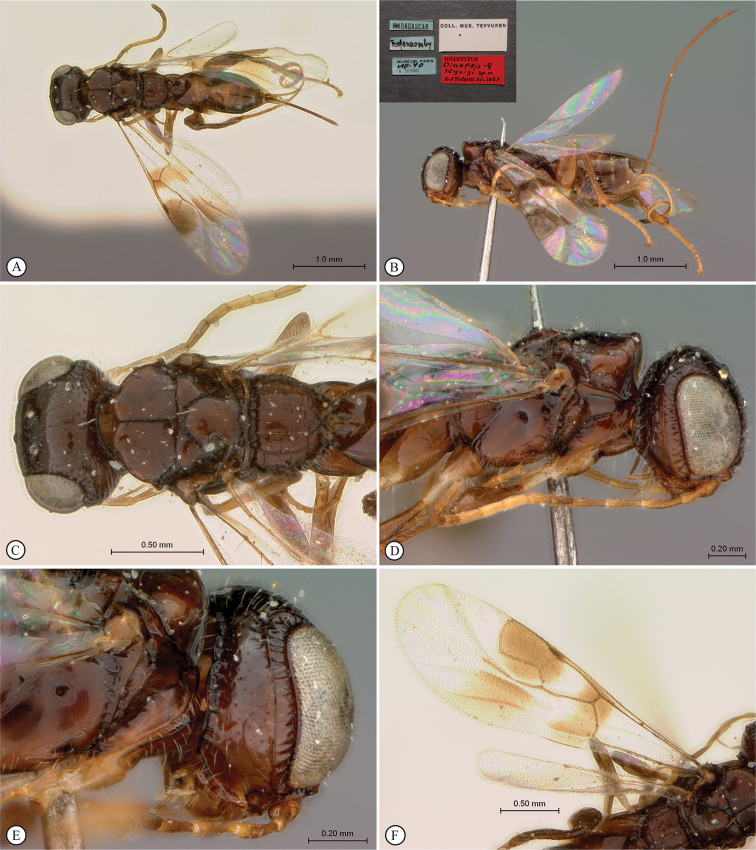
*Dinapsisseyrigi* holotype female (MNHN) **A** habitus, dorsal view **B** habitus, lateral view **C** head, mesosoma, dorsal view **D** head, mesosoma, lateral view **E** head, posterolateral view **F** wings, dorsal view. Scale bars: 1000 µm (**A, B**); 500 µm (**C, F**); 200 µm (**D, E**).

The type-locality of Trafanomby is now located inside the Androhahela National Park. *Dinapsisseyrigi* can be easily distinguished from all other species treated in this paper by the smooth (unsculptured) condition of the gena (Fig. 20E).

#### ﻿ Dinapsis spinitibia

Taxon classificationAnimaliaHymenopteraMegalyridae

van Noort & Shaw sp. nov.

114B3B8B-A5E0-5F38-99B0-A8519414E3E3

https://zoobank.org/4C2CB71C-5303-4261-AD62-53B9734DDA5F

Figs 21 , 22 , 23

##### Material examined.

***Holotype*.** Tanzania • ♂; Tanga, Muheza Dist. Kwamgumi For. Res.; 4°57S 38°44E; 170–220 m a.s.l.; 9 Nov. 1995; Fog 18-CD, NHMD, Denmark; U. Copenhagen UDSM Canopy Fogging Project; S.H. McKamey et al. leg.; *Dinapsis* sp. ♂, Megalyridae; IMAGED WaspWeb LAS 4.9 SAMC 2021 (yellow label); HOLOTYPE *Dinapsisspinitibia* van Noort & Shaw, 2022 (red label); NHMD.

##### Diagnosis.

**Figure 21.**
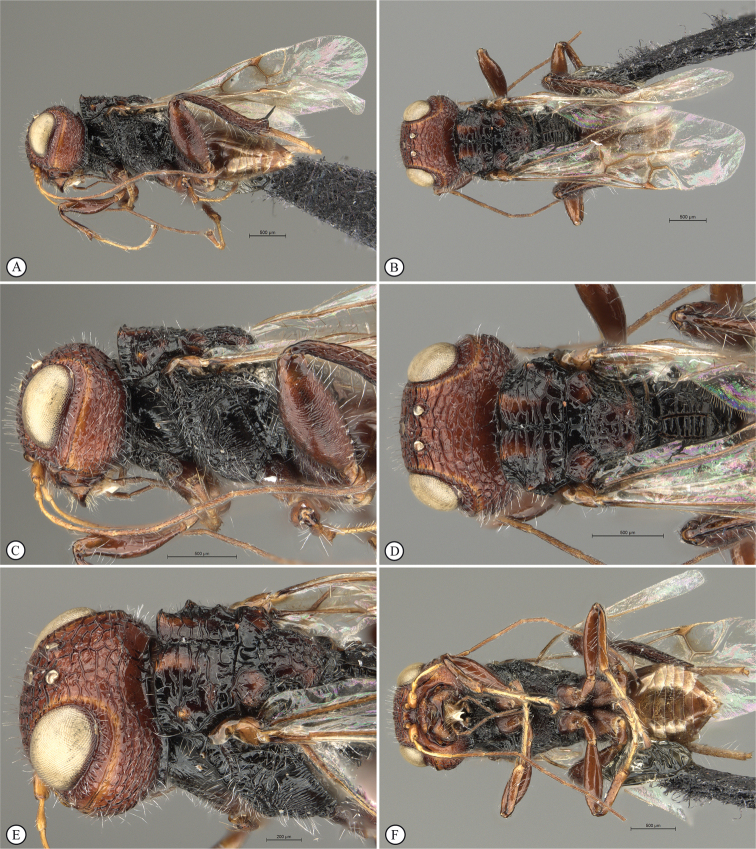
*Dinapsisspinitibia* van Noort & Shaw, sp. nov. holotype female (NHMD) **A** habitus, lateral view **B** habitus, dorsal view **C** head, mesosoma, lateral view **D** head, mesosoma, dorsal view **E** head, mesosoma, dorsolateral view **F** habitus, ventral view. Scale bars: 500 µm (**A–D, F**); 200 µm (**E**).

Extremely stout bodied, uniquely with two sharply elongate, parallel, stout spines positioned in close apposition on hind tibial apex (Fig. 23A–F). Head with strong coarsely foveate sculpture and massive genae. Mesosoma strongly sculptured with raised transverse mesoscutal carinae, projecting into dorsolateral teeth (Fig. 21E).

##### Distribution.

(Fig. 44) Tanzania.

**Figure 22.**
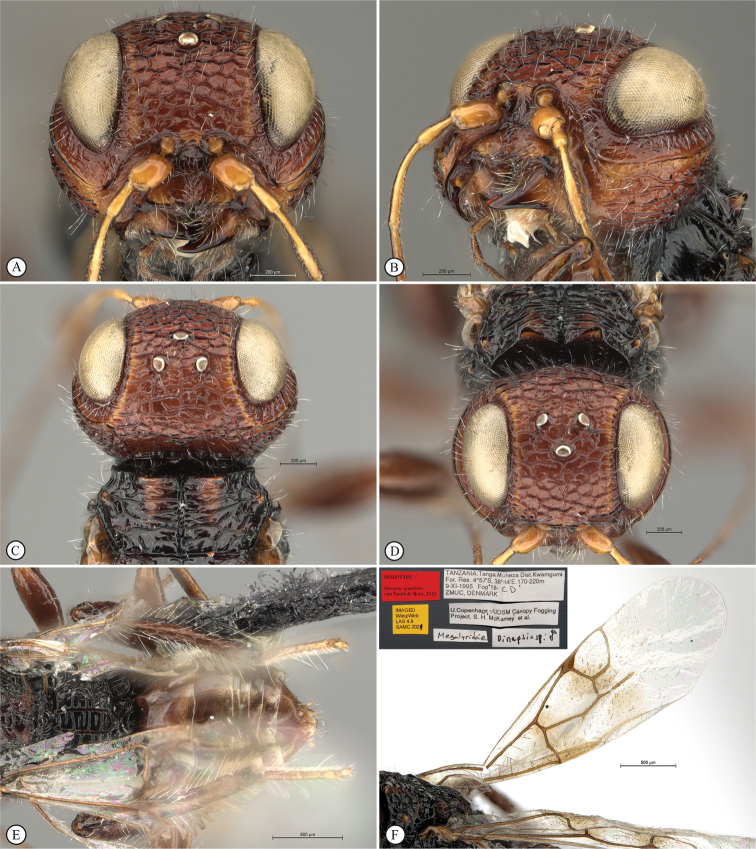
*Dinapsisspinitibia* van Noort & Shaw, sp. nov. holotype female (NHMD) **A** head, anterior view **B** head, ventrolateral view **C** head, mesoscutum, dorsal view **D** head, mesoscutum, dorso-anterior view **E** propodeum, metasoma, dorsal view **F** wings, dorsal view. Scale bars: 200 µm (**A–D**); 500 µm (**E, F**).

##### Comments.

*Dinapsisspinitibia* is known only from the Kwamgumi Forest Reserve.

**Figure 23.**
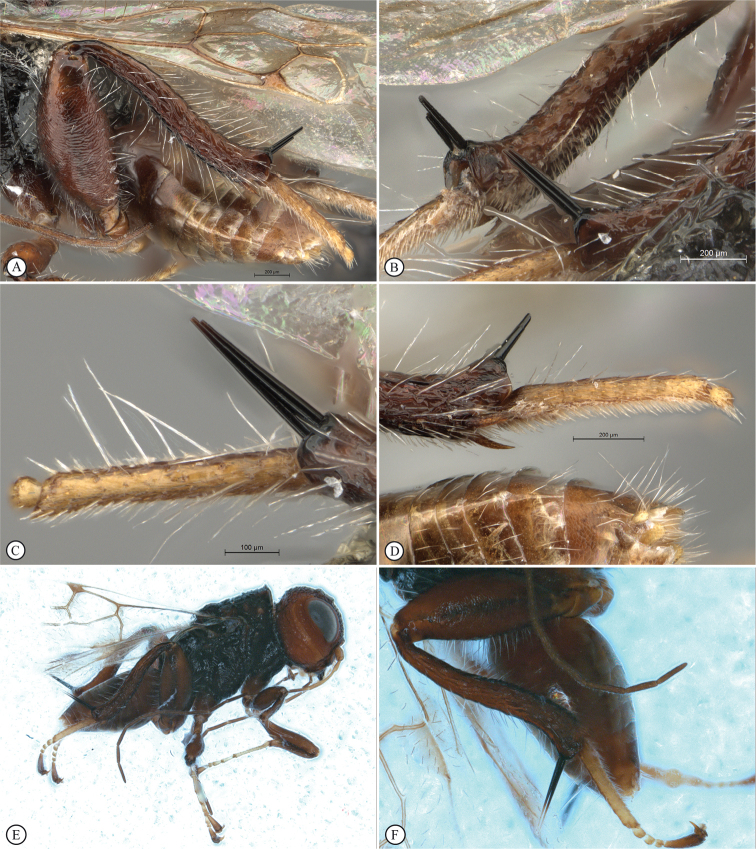
*Dinapsisspinitibia* van Noort & Shaw, sp. nov. holotype female (NHMD) **A** hind leg, antiaxial view **B** tibial spines, lateral view **C** tibial spines, basitarsus, antiaxial view **D** tibial spines, basitarsus, axial view **E** habitus, lateral view (specimen in ethanol prior to being mounted) **F** hind leg antiaxial view (specimen in ethanol prior to being mounted). Scale bars: 200 µm (**A, B, D**); 100 µm (**C**).

##### Etymology.

This species is named after the sharply pointed, stout spines present on the apex of the hind tibia (Fig. 23A–F). Noun in apposition.

##### Description.

**Holotype female. *Body*** length 3.6 mm.

***Colour*.** Head orange-brown; mandibles grading to dark brown in apical 1/2. Mesosoma with black ground plan, dorsally diffused with small orange-brown areas on mesoscutum. Pronotum, mesopleuron and propodeum black. Metasoma light brown. Head with a covering of sparse white setae which are longer on face and frons. Scape, pedicel, F1 and basal F2 yellowish brown, F3–F12 orange-brown. Coxae and trochanters orange-brown, femora and tibiae dark orange-brown, tarsi yellowish brown. Eyes and ocelli silvery. Wing membrane clear except for two light brown pigmented patches extending partly across forewing as semi-bands.

***Head*** oval, 1.25 × wider than high; vertex, frons, and face coarsely foveate; ocelli small, OOL 1.6 × ocellar diameter; all ocelli bounded by a partial semi-circular depression on the side facing outer edge of the triangle; ocellar triangle equilateral; eye large and slightly protuberant, medially parallel in anterior view, but diverging dorsally and ventrally; eye devoid of ocular setae; eye margined posteriorly by wide foveate groove bounded by strong carina; these carinae extend through genae to meet anteriorly above toruli; foveae absent in section of groove present on gena; genae massive; postocular orbital carina weakly present; antenna with 12 thin, elongate flagellomeres having flagellar length/width ratios as follows: F1 = 8.5 (very narrow basally), F2-F4 = 10.0, F5 = 6.7, F6-F7 = 6.4, F8 = 6.0, F9 = 7.0, F10 = 5.7, F11 = 5.2, F12 = 7.3; temple adjacent to ocular orbital carina coarsely foveate, temple large, width 1.4 × eye width in lateral view; malar length equivalent to mandible width basally; occiput largely polished between postocular carina and occipital carina; occipital carina evenly wide, crenulate.

***Mesosoma*.** Pronotum polished, laterally excavated with a row of large oblong foveae situated posteriorly on the margin with the mesopleuron, no foveae along dorsolateral margin. Mesoscutal anterior plate polished, with a medial suture grading into a row of punctures, anterior plate with strong lateral carinae extending dorsally into two teeth-like projections; mesoscutum 0.9 × wider than long, with strong transverse carina extending into two lateral, dorsally projecting teeth; sparse, short setae; anterodorsally with two diffuse parallel orange-brown elongate patches; axillae and posterolaterally on the scutellum with diffuse orange-brown patches; medial mesoscutal furrow with jagged edges and foveae; transscutal articulation a smooth, narrow furrow, anterior edge largely straight except medially, posterior edge straight; scutoscutellar sulcus poorly defined, absent medially, laterally comprising three elongate foveae; scutellar disc strongly rugulose, with scattered erect, short white setae; mesopleuron coarsely foveate with short white setae, in posterior medial 1/2 polished area grading into striations anteriorly and abutting vertical row of foveae posteriorly, with large median mid-pit. Metanotum with raised, setose medial area flanked laterally by depression with 3–5 foveae. Propodeum medially polished with strong transverse carinae between the submedian longitudinal carinae defining the three central tracks; lateral longitudinal tracks with defined transverse carinae. All five tracks anteriorly with deep foveae.

***Legs*.** All legs with white setae. Apex of fore and mid tibiae with comb of four or five stout spines; hind coxa shagreened, with sparse, small, white setae; hind femur stout, shagreened, 2.4 × longer than wide, with strong, long, erect white setae projecting from dorsal and ventral margin, outer surface of hind femur sparsely, covered with short, erect, white setae; inner surface of hind femur polished with very short setae; surface of hind tibia rugulose, with two sharp stout, but elongate black (grading to white apically) spines, in close apposition, projecting from dorsoapical margin (almost as long as basitarsus 0.83 ×); two hind tibial spurs strong, curved, longitudinally striate with minute setae; hind tibia with long, erect, white setae dorsally and ventrally, shorter setae laterally; dorsal setae lacking spatulate tips; inner ventral margin of hind tibia with a dense longitudinal patch of shorter white setae; hind basitarsus long, 1.4 × length of remaining four tarsomeres combined; basitarsus ventrally with dense preening brush consisting of numerous short, white setae, inclined posteriorly; basitarsus dorsally with two or three strong, long, erect white setae, lacking spatulate tips; T2 and T3 slightly longer than wide, T4 ca. as long as wide, T5 3.4 × as long as wide; all tarsomeres with normal small hair-like setae; tarsal claw simple, weakly curved.

***Wings*.** Forewing length 3.15 mm, 3.3 × longer than wide; wing basally with cells R and 1A largely devoid of setae, but with widely spaced strong erect setae projecting from surrounding veins; remaining wing surface evenly, but sparsely covered with small, scattered setae; basally and medially with scattered stronger, short setae; wing clear, with two light brown pigmented patches, positioned in two vertical semi-bands. Basal wing band narrowest dorsally, covering basal 1/10 of cell 1M, posterior 1/5 of cell R, and anterior 1/8 of cell 1Cu, extending ventrally to wing margin, diffusely covering basal 1/2 of cell 2CU and all of 3A, both of which are hardly demarcated by ephemeral veins; apical wing band reduced to two patches; dorsal patch starting at base of pterostigma, and anterior end of 1R1, extending apically to cover basal posterior quarter of marginal cell 2R1, ventrally to cover almost entire cell 1+2RS, ventrally wider and more diffuse; ventral patch starting below vein M and extending to but not reaching posterior margin of wing; forewing venation with vein Rs apically curving abruptly towards anterior wing margin to form short, truncate marginal cell 2R1; apical segment of vein M long, extending beyond apex of marginal cell, vein M with small white bulla 1/3 of vein length. Hind wing with apical stub of vein Rs 2/3 of shortest width between the propodeal submedian longitudinal carinae.

***Metasoma*** in dorsal view 1.3 × as long as wide, with seven dorsally visible terga, all polished, with row of long erect, white setae, terminally with white setae in dense patch; pygostyles long (as long as hind tarsus 2), elongate, projecting.

#### ﻿ Dinapsis taita

Taxon classificationAnimaliaHymenopteraMegalyridae

van Noort & Shaw sp. nov.

4E33C982-1318-5B8F-8EDE-976D001E9C86

https://zoobank.org/D1DC2FDD-3CD5-4887-98B1-7F13EFB01AB5

Figs 24 , 25 , 26

##### Material examined.

***Holotype*.** Kenya • ♀; Taita Hills, Ngangao Forest; 3.36100°S, 38.34186°E; 1848 m a.s.l.; Malaise trap; indigenous forest; 3–17 Oct. 2012; R. Copeland leg.; ICIPE 49131; NMKE. ***Paratype*.** Burundi • 1 ♀; Ruvubu N. P.; Malaise trap; 7–25 May 2010; R. S. Copeland leg.; ICIPE 49132; IBOL ID 08672-MEGSPBURH9; ICIPE.

##### Diagnosis.

**Figure 24.**
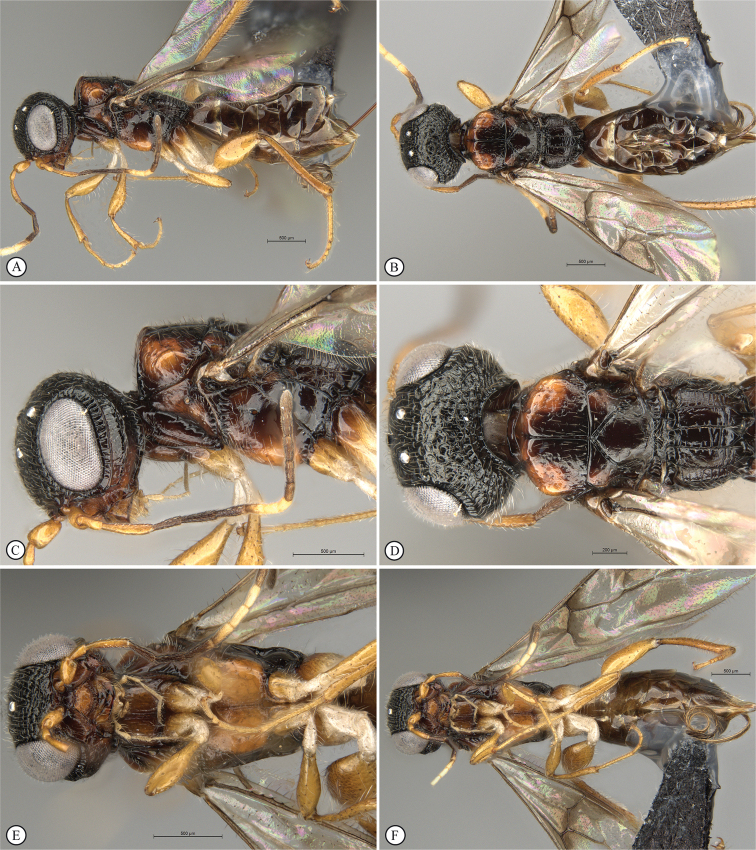
*Dinapsistaita* van Noort & Shaw, sp. nov. holotype female ICIPE 49131 (NMKE) **A** habitus, lateral view **B** habitus, dorsal view **C** head, mesosoma, lateral view **D** head, mesosoma, dorsal view **E** head, mesosoma, ventral view **F** habitus, ventral view. Scale bars: 500 µm (**A–C, E, F**); 200 µm (**D**).

Morphologically similar to *D.tricolor*, however, *D.taita* may be distinguished by its mesoscutum being more weakly foveate and having light orange-brown colour patches on the mesoscutal lobes (Fig. 24D). Head coarsely punctate, with sparse setae; mesoscutal plate and antero-lateral mesoscutal knobs orange-brown; scutoscutellar sulcus demarcated by narrow furrow; wing with brown bands, apical light infuscation reaching anterior wing margin; legs yellowish brown, coxae white ventro-laterally; hind tibia with long, pale setae. This species is also morphologically similar to *D.gamka*, both species having a polished mesoscutum with distinctly raised anterior knobs, but only the very peaks of the knobs may be faintly orange-brown in *D.gamka*, whereas the mesoscutal plate and larger areas of the dorsal surface encompassing the raised knobs are distinctly and widely orange-brown in *D.taita*.

##### Distribution.

(Fig. 44) Burundi, Kenya.

##### Comments.

*Dinapsistaita* is known from single sites in Kenya and Burundi, the latter representing a new country record for the genus. In Kenya, the species was collected in the large, wet Ngangao forest in the Taita Hills at an elevation of 1848 m. To our knowledge this is the highest elevation of any *Dinapsis* species.

**Figure 25.**
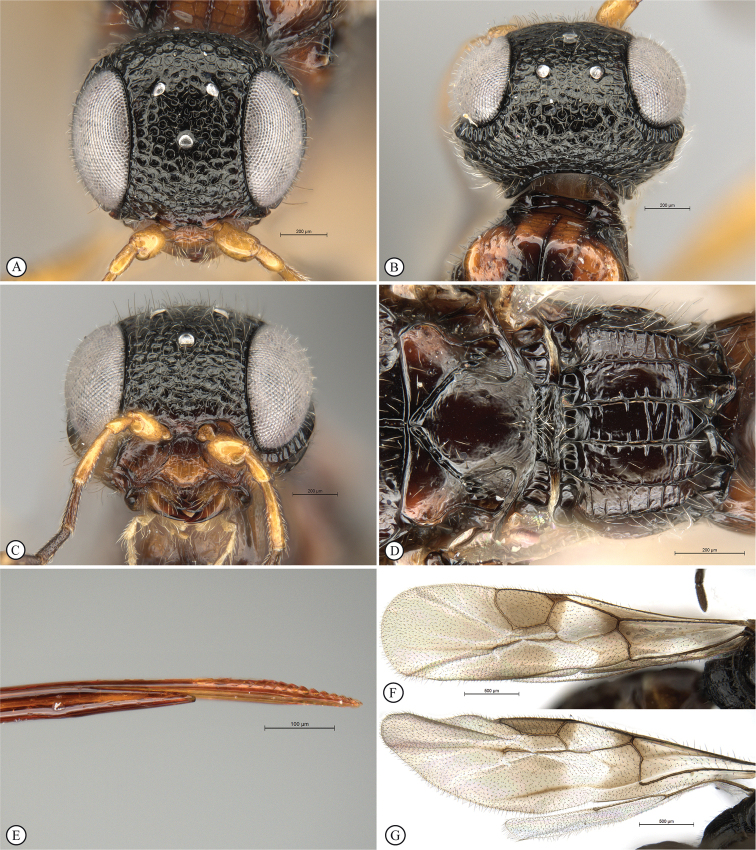
*Dinapsistaita* van Noort & Shaw, sp. nov. holotype female ICIPE 49131 (NMKE) **A** head, dorsal view **B** head, dorsoposterior view **C** head, anterior view **D** mesoscutellum, propodeum, dorsal view **E** terminal ovipositor valves, lateral view **F** forewing, dorsal view **G** forewing, dorsal view (wing is strongly curved, necessitating two images to depict cell shape. Scale bars: 200 µm (**A–D**); 100 µm (**E**); 500 µm (**F, G**).

##### Etymology.

This species is named after the Taita Hills encompassing the holotype locality. Noun in apposition.

**Figure 26.**
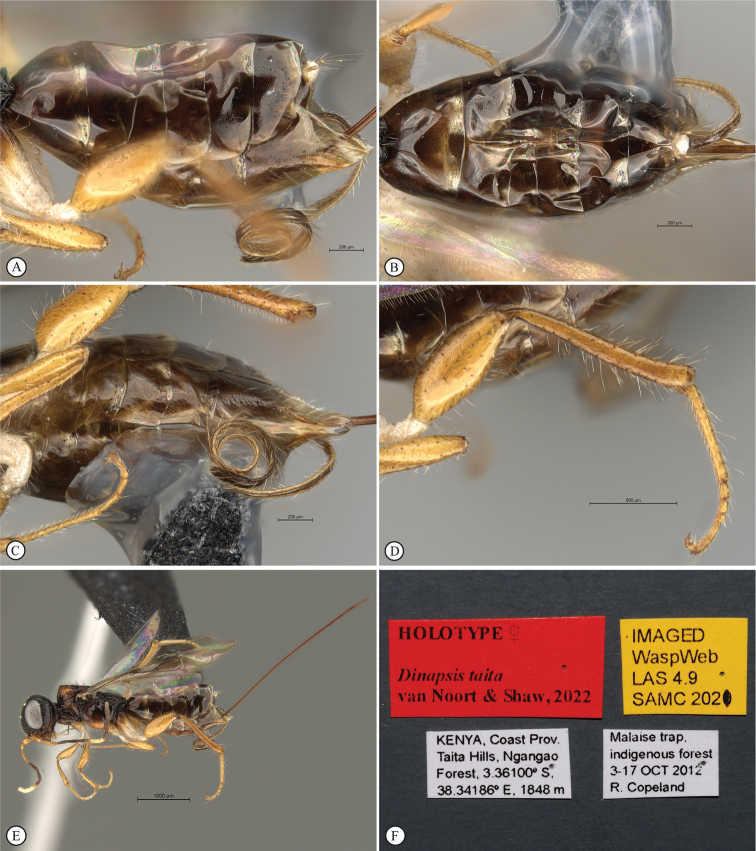
*Dinapsistaita* van Noort & Shaw, sp. nov. holotype female ICIPE 49131 (NMKE) **A** metasoma, lateral view **B** metasoma, dorsal view **C** metasoma, ventral view **D** hind leg antiaxial view **E** habitus, lateral view **F** data labels. Scale bars: 200 µm (**A–C**); 500 µm (**D**); 1000 µm (**E**).

##### Barcode sequence for paratype specimen from Burundi.

(sequence code in BOLD:KINS1609-11) BIN URI: BOLD:AAZ9109.

##### Nucleotide sequence.

TTCTTTGTATTTTATTTTTGCTATTTGATCTGGTTTAATTGGATCTTCATTAAGTATAATTATTCGAATAGAATTAAGAGTTCCGGGTTCTTTTATTGGTAATGATCAAATTTATAATTCTATTGTTACAGCTCATGCATTTATTATAATTTTTTTTATAGTTATACCTTTTATAATAGGTGGATTTGGAAATTGACTTCTTCCTTTAATATTAGGGGCTCCTGATATATCTTATCCTCGTTTGAATAATTTAAGATTTTGATTATTAATCCCTTCTTTAATATTTTTGTTAATAAGATTTTATGTTGGCAGAGGAACAGGAACTGGATGAACAGTTTATCCTCCTTTATCGTCTAATACATTTCATTCTAGAATAAGAGTAGATTTATCTATTTTTAGTCTTCATATTGCTGGTATTTCATCAATTTTAGGATCTGTAAATTTTATTTCTACAATATTAAATTTACAGCATGTTTATTTAAAATTAAATAGACTTAGTTTATTTATTTGATCTGTTTTTTTGACAGCTATTTTACTTTTATTATCTTTACCTGTATTAGCAGGTGCAATTACAATATTATTGACTGATCGTAATTTAAATACAACTTTTTTTGATCCCTCGGGGGGGGGGGANCCAATTTTATATCAACNTCTTTTT.

##### Description.

**Holotype female. *Body*** length 4.6 mm excluding ovipositor.

***Colour*.** Head mostly black except for genae, face, clypeus and mandibles which are orange-brown. Mesosoma with black ground plan diffused with orange areas on mesoscutal knobs and mesoscutal plate, and ventral mesopleuron. Mesopleuron laterally, and pronotum diffuse orange-brown. Propodeum black. Metasoma dark brown. Head with a covering of white setae on occiput, smaller brown setae on face and frons. Scape, pedicel and F1 yellowish brown, F2–F4 dark brown, F5 basally light brown grading to whitish yellow, F6 and F7 whitish yellow and F8–F12 light brown. Coxae ventrally and trochanters white; coxae laterally and dorsally grading to orange-brown, Rest of legs yellowish brown. Ovipositor orange-brown. Eyes and ocelli silvery. Wing membrane clear except for two broad light brown pigmented bands across forewing with the apical band not extending as infuscation towards apical margin.

***Head*** oval, 1.38 × wider than high; vertex, frons, and face coarsely punctate, interstices absent; ocelli small, OOL twice ocellar diameter; only lateral ocelli bounded by a partial semi-circular depression on the side facing outer edge of the triangle; ocellar triangle an isosceles (POL:LOL - 6:7); eye large and hardly protuberant, not parallel in anterior view, strongly diverging dorsally and ventrally; eye densely and evenly covered with minute white ocular setae; eye margined posteriorly by foveate groove; postocular orbital carina weakly present; antenna with 12 flagellomeres having flagellar length/width ratios as follows: F1 = 4.6, F2-F3 = 4.0, F4 = 3.0 F5-F11 = 2.5 F12 = 1.5; apical flagellomeres slightly wider than basal flagellomeres; temple adjacent to ocular orbital carina coarsely punctate, temple width 0.7 × eye width in lateral view; malar length equivalent to mandible width basally; occiput coarsely punctate; occipital carina wide laterally, narrower dorsally and crenulate.

***Mesosoma*.** Pronotum polished, laterally excavated with a row of large oblong foveae situated posteriorly on the margin with the mesopleuron, no foveae along dorsolateral margin, dorsally with transverse excavation. Mesoscutal anterior plate polished, with a medial suture grading medially into a row of approximately five punctures, and a lateral carina bounded laterally by weak foveae; mesoscutum as wide as long, mesoscutal lobes polished, with scattered shallow punctures and sparse, short setae, antero-lateral areas orange, smoothly rounded except for shallow punctures; sparse shallow foveae scattered posterolaterally; medial mesoscutal furrow deep, narrow and straight edged; transscutal articulation a smooth, narrow furrow, anterior edge straight, posterior edge straight; scutoscutellar sulci medially comprising a continuous shallow groove with very weakly defined septa, laterally grading into three large separate foveae; anteriorly meeting before reaching transscutal articulation; scutellar disc medially polished, with scattered erect, white setae laterally and posteriorly; mesopleuron anteriorly shallowly foveate with short white setae, in anterior 1/2 medially polished, with large median mid-pit. Metanotum with raised, setose medial area flanked laterally by depression with 3–5 foveae. Propodeum medially polished with strongly reduced transverse carinae between the submedian longitudinal carinae defining the three central tracks; lateral longitudinal tracks with defined transverse carinae. All five tracks anteriorly with two or three deep foveae.

***Legs*.** All legs with white setae, each seated in a dark basal socket, contrasting with surrounding pale integument creating spotted appearance. Apex of fore tibia with comb of stout spines; hind coxa polished, with sparse, small, white setae; hind femur stout, polished, 2.4 × longer than wide, outer surface of hind femur sparsely covered with short, erect, white setae; inner surface of hind femur polished with very short setae; surface of hind tibia polished, with long erect white setae dorsally and ventrally, shorter setae laterally; dorsal setae lacking spatulate tips; inner ventral margin of hind tibia with a dense longitudinal patch of shorter white setae; hind basitarsus long, 1.3 × length of remaining four tarsomeres combined; basitarsus ventrally with dense preening brush consisting of numerous short, white setae, inclined posteriorly; basitarsus dorsally with normal long, white setae, lacking spatulate tips; T2 and T3 twice as long as wide, T4 ca. as long as wide, T5 4.5 × as long as wide; all tarsomeres with normal hair-like setae, but also with scattered elongate, stronger setae projecting from dorsal surface; tarsal claw simple, strongly curved.

***Wings*.** Forewing length 3.3 mm, 3 × longer than wide; wing basally with cells C, R and 1A with longer, more sparsely spaced setae; remaining wing surface evenly covered with small, scattered setae; wing clear, with two light brown pigmented vertical bands. Basal wing band narrowest medially, covering ventral medial portion of cell C, basal 1/2 of cell 1M, anterior ends of cells R and 1Cu, extending ventrally to wing margin, covering all of cell 2CU and 3A, both of which are only weakly demarcated by ephemeral veins; apical wing band wider, starting at base of pterostigma, and anterior end of 1R1, extending apically to cover entire marginal cell 2R1, posteriorly to cover entire cell 1+2RS, ventrally wider and more diffuse, with infuscate pigmentation extending across cells 2+3M and 3CU, and reaching lower wing margin; light infuscation covering medial section of apical wing area; forewing venation with vein Rs apically curving abruptly towards anterior wing margin to form short, truncate marginal cell 2R1; apical segment of vein M long, extending beyond apex of marginal cell, vein M with small white bulla situated at mid length of vein. Hind wing with apical stub of vein Rs 2/3 of shortest width between the propodeal submedian longitudinal carinae.

***Metasoma*** in dorsal view 2 × as long as wide, with seven dorsally visible terga, all polished; exposed portion of ovipositor, in lateral view 2.1 × longer than metasomal length; dorsal valve with approximately 14 serrations, ventral valve smooth; ovipositor sheaths setose, strongly curled (an artefact of preservation).

#### ﻿ Dinapsis tricolor

Taxon classificationAnimaliaHymenopteraMegalyridae

Shaw & van Noort sp. nov.

243BED58-C692-5876-9ED7-C11E706CDF5F

https://zoobank.org/54945EDA-135B-4730-B227-703D4ACDBB92

**Figure 27.**
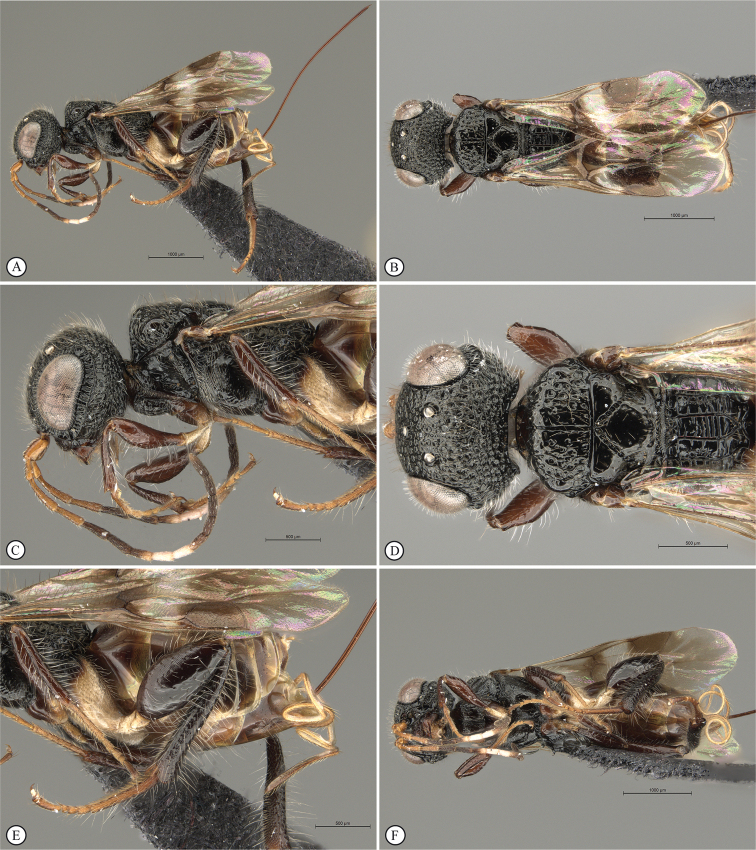
*Dinapsistricolor* Shaw & van Noort, sp. nov. holotype female (NMSA) **A** habitus, lateral view **B** habitus, dorsal view **C** head, mesosoma, lateral view **D** head, mesosoma, dorsal view **E** metasoma, legs, lateral view **F** habitus, ventral view. Scale bars: 1000 µm (**A, B, F**); 500 µm (**C–E**).

**Figure 28.**
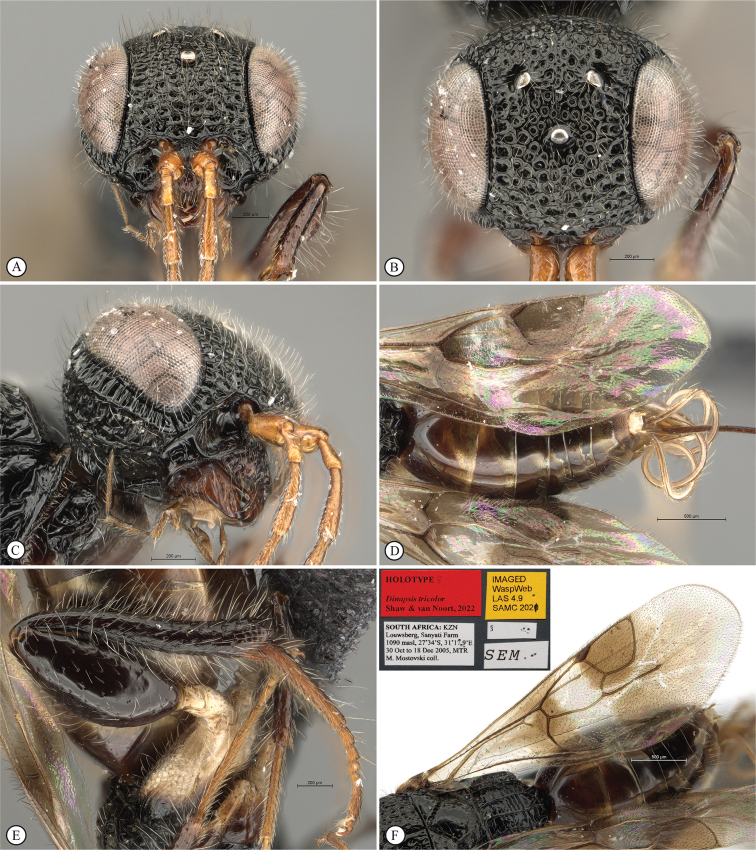
*Dinapsistricolor* Shaw & van Noort, sp. nov. holotype female (NMSA) **A** head, anterior view **B** head, dorsal view **C** head, anteroventral view **D** metasoma, dorsal view **E** hind leg, antiaxial view **F** wings, dorsal view (inset: data labels). Scale bars: 200 µm (**A–C, E**); 500 µm (**D, F**).

**Figure 29.**
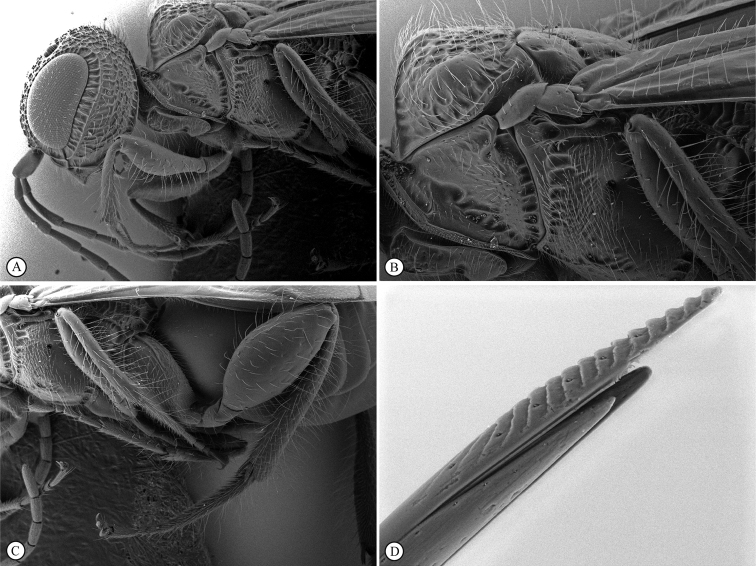
*Dinapsistricolor* Shaw & van Noort, sp. nov. holotype female, SEM (NMSA) **A** head, mesosoma, lateral view **B** mesosoma, lateral view **C** mid and hind legs, lateral view **D** ovipositor terminal valves, lateral view.

**Figure 30.**
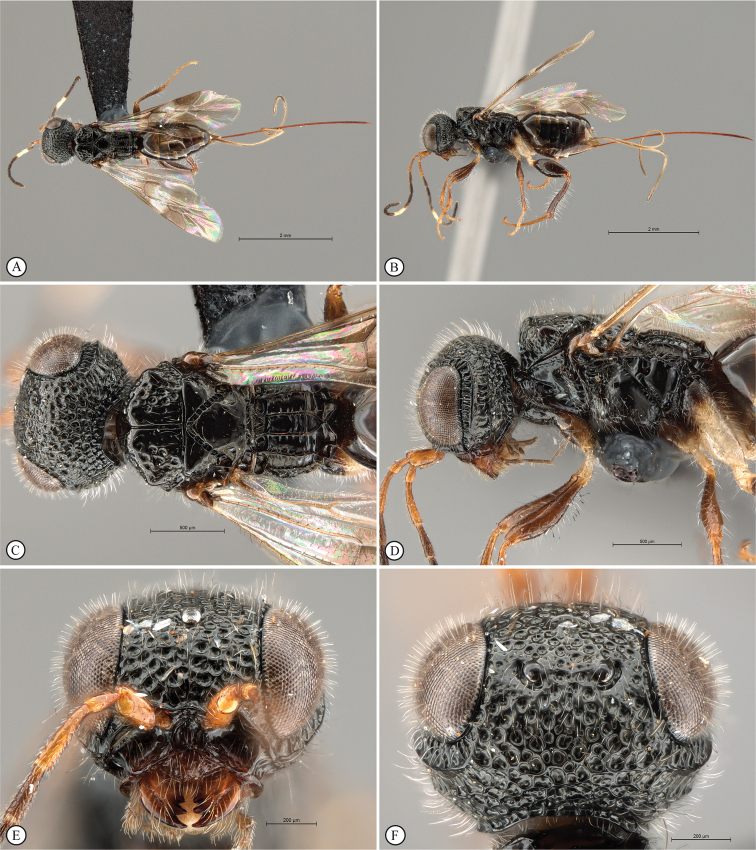
*Dinapsistricolor* Shaw & van Noort, sp. nov. paratype female, Kenya ICIPE 49121 (ICIPE) **A** habitus, dorsal view **B** habitus, lateral view **C** head, mesosoma, dorsal view **D** head, mesosoma, lateral view. **E** head, anterior view **F** head, dorsal view. Scale bars: 2000 µm (**A, B**); 500 µm (**C, D**); 200 µm (**E, F**).

**Figure 31.**
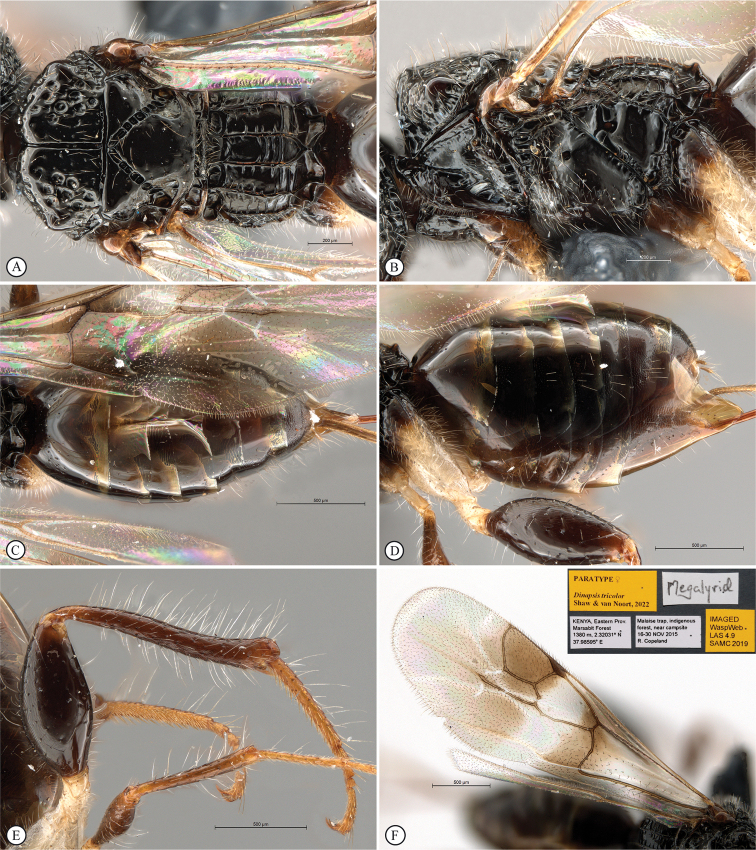
*Dinapsistricolor* Shaw & van Noort, sp. nov. paratype female, Kenya ICIPE 49121 (ICIPE) **A** mesosoma, dorsal view **B** mesosoma, lateral view **C** metasoma, dorsal view **D** metasoma, lateral view **E** hind and mid legs, antiaxial view **F** wings, dorsal view (inset: data labels). Scale bars: 200 µm (**A, B**); 500 µm (**C–F**).

**Figure 32.**
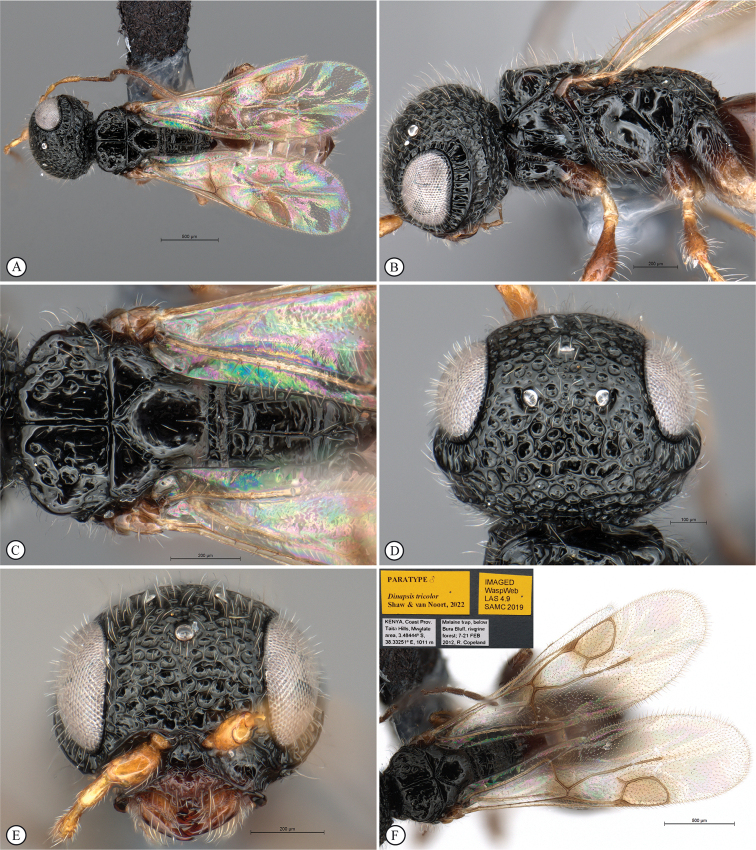
*Dinapsistricolor* Shaw & van Noort, sp. nov. paratype male, Kenya ICIPE 49129 (NMKE) **A** habitus, dorsal view **B** head, mesosoma, lateral view **C** mesosoma, dorsal view **D** head, dorsal view **E** head, anterior view **F** wings, dorsal view (inset: data labels). Scale bars: 500 µm (**A, F**); 200 µm (**B, C, E**); 100 µm (**D**).

Figs 27 , 28 , 29 , 30 , 31 , 32

##### Material examined.

***Holotype*.** South Africa • ♀; KwaZulu-Natal, Louwsberg, Sanyati Farm; 1090 m a.s.l.; 27°34'S, 31°17.9'E; 30 Oct.–18 Dec. 2005; Malaise trap (MTR); M. Mostovski leg.; NMSA-HYM 002030; NMSA. ***Paratypes*.** South Africa • 2 ♀♀; same data as holotype NMSA-HYM 000547; NMSA-HYM 000548; NMSA • 2 ♀♀; La Mercy, site 1;85 m a.s.l.; 29°37'41.0"S, 31°06'45.4"E; yellow pan trap; M. Mostovski leg.; NMSA-HYM 000545; NMSA-HYM 000546; NMSA • 1 ♂; Eshowe, nr. Ntumeni N.R.; 680 m a.s.l.; 28°52'08"S, 31°22'41"E; 20 Apr.–26 Oct. 2007; Malaise trap; Kolyada and Mostovski leg.; NMSA-HYM 000544; NMSA • 2 ♀♀; KwaZulu-Natal, Ndumo Game Reserve; [26.881013°S, 32.252109°E]; 7–8 Nov. 2002; C. Desjardins leg.; yellow pan trap; USNM. KENYA • 1 ♀; Eastern prov. Marsabit Forest; 1380 m a.s.l.; 2.32031°N, 37.98595°E; Malaise trap; indigenous forest; near campsite; 16–30 Nov. 2015; R. Copeland; ICIPE 49121; ICIPE • 1 ♀; Coast Province, Mrima Hill Forest; 212 m a.s.l.; 4.48576°S, 39.25845°E; Malaise trap; edge of indigenous forest; 25 Dec. 2011–8 Jan. 2012; ICIPE 49122; R. Copeland leg.; ICIPE; • 1 ♀; Eastern Province, Njuki-ini Forest; Malaise trap; 17–31 Jul. 2006; ICIPE 49123; R. Copeland leg.; ICIPE • 1 ♀; same data except 17–30 Jan. 2007; ICIPE 49124; UWIM • 1 ♂; Eastern Province, Njuki-ini Forest near Forest Station; 1455 m a.s.l.; 0.51660°S, 37.41843°E; Malaise trap; just inside indigenous forest; 14–28 Aug. 2007; R. Copeland leg.; ICIPE 49125; ICIPE • 1♂; Eastern Province, Njuki-ini Forest near Forest Station; 1471 m a.s.l.; 0.51663°S, 37.41852°E; Malaise trap; just inside indigenous forest; 21 Jul.–4 Aug. 2008; R. Copeland leg.; ICIPE 2140; ICIPE • 1 ♀; Coast Province, Shimba Hills, Longomwagandi Forest; 389 m a.s.l.; 4.23456°S, 39.41687°E; 27 Aug.–10 Sep. 2008; Malaise trap; R. Copeland leg.; ICIPE 49126; ICIPE • 1 ♂; same data except 23 Oct. –5 Nov. 2008; ICIPE 2828; ICIPE • 1 ♀; Nyanza Province, Gwasi Hill, near top; 29 Jul.–12 Aug. 2005; R.S. Copeland leg.; ICIPE 49127; ICIPE • 1 ♀; Coast Prov., Buda Forest; 98m a.s.l.; 4.46109°S, 39.40446°E; Malaise trap in indigenous forest; 22 Apr. –6 May 2016; ICIPE 49128; ICIPE • 1 ♂; same data except 6–20 May 2016; 4056-66; ICIPE 10305; SAMC • 1 ♀; Coast Prov., Muhaka Forest; 54m; 4.32575°S, 39.52323°E; Yellow pan trap in indigenous forest; Apr.–July 2018; R. Copeland leg.; 0815-20; ICIPE 10306; ICIPE • 1 ♂; Coast Prov., Taita Hills, Mwatate area; 1011 m a.s.l.; 3.48444°S, 38.33251°E; Malaise trap below Bura Bluff; riverine forest; 7–21 Feb. 2012; R. Copeland leg.; ICIPE 49129; NMKE • 1 ♂; Coast Prov., Kasigau Mtn.; indigenous forest; 1065 m a.s.l.; 3.82700°S, 38.64875°E; Malaise trap next to campsite in forest; 14–28 Dec. 2011; R. Copeland leg.; ICIPE 49130; ICIPE • 1 ♀; Eastern Prov., Gai Hill, near base of; 959 m a.s.l.; 0.60992°S, 38.18894°E; Malaise trap; in indigenous forest; 7–21 Dec. 2021 R. Copeland leg.; ICIPE 49459; ICIPE.

##### Diagnosis.

Morphologically similar to *D.taita*, however, *D.tricolor* may be distinguished by the mesoscutum being coarsely foveate and usually entirely black (Figs 29C, 31A). In the key to African *Dinapsis* species by Hedqvist (1967), *D.tricolor* keys to couplet 2 because of the presence of minute ocular setae, a characteristic shared with *Dinapsiszulu*, which also occurs in KwaZulu-Natal. *Dinapsistricolor* is easily distinguished from *Dinapsiszulu* by the latter’s smaller body size, shorter erect setae dorsally on the head vertex and metanotum; temple and gena rugose, with two postocular carinae, and more compact metasoma. Females of *D.tricolor* have a distinctive tri-coloured antenna, with the scape, pedicel, and F1-F2 orange, F3-F4, F5 basally, and F8-F12 dark brown, and a pale brownish white flagellar band on F5 (apically), F6, and F7. *Dinapsisoculohirta* is a smaller species (females < 3 mm), with more densely setose eyes, smooth vertex with a medial row of punctures between ocelli, two distinct postocular orbital carinae, smooth mesoscutal lobes, scutoscutellar sulcus demarcated by row of punctures, more pale forewing bands (not filling the marginal cell), and propodeum medially lacking transverse carinae between the submedian longitudinal carinae. In contrast, *D.tricolor* is a larger species (females 3.5–5.5 mm) with a coarsely foveate-reticulate vertex, the second postocular orbital carina either absent or irregular and indistinct, coarsely foveate-reticulate mesoscutal lobes, scutoscutellar sulcus demarcated by sulcus, dark forewing band completely filling the marginal cell, and propodeum medially with weakly developed transverse carinae between the submedian longitudinal carinae. *Dinapsisoculohirta* is only known to occur in Madagascar, while *D.tricolor* is only known from continental Africa. In colour and size, *D.tricolor* is somewhat similar to *D.centralis* Shaw & van Noort, which also occurs in Kenya. However, the mostly dark brown flagellum (lacking a strongly contrasting pale-coloured median band) of *D.centralis* easily distinguishes females of this species from *D.tricolor*, females of which have a pale yellowish white band on flagellomeres 6 and 7. Another curious and distinctive character of *D.centralis* is the presence, on the hind tibia and basistarsus, of large erect dorsal setae, some of which have expanded spatulate tips. The setae on *D.tricolor* are smaller and hair-like, lacking spatulate tips.

##### Distribution.

(Fig. 44) Kenya, South Africa (KwaZulu-Natal Province).

##### Comments.

*Dinapsistricolor* is only known from forested localities in Kenya and South Africa but is likely to be present all along the East African coastal regions from Kenya to South Africa, a sub-biogeographical unit comprising similar faunal and floral assemblages throughout the region. All 14 Kenyan specimens of *D.tricolor* were collected in wet, canopy-forest habitats. Four of the sites are small relict coastal forests (Mrima Hill, Muhaka, Buda, and Longomwagandi forest in the Shimba Hills National Park). The latter is under the jurisdiction of the Kenya Wildlife Service while the others are the responsibility of the Kenya Forest Service. Mrima, Muhaka and Buda are also kayas, sacred forests where cultural and religious activities are still held. As such they also receive a measure of protection from encroachment through the overview of local elders. Kasigau mountain forms, along with the nearby Taita Hills, the northernmost extension of the eastern arc mountains, a chain of ancient non-volcanic crystalline mountains centred mostly in Tanzania. *Dinapsistricolor* was also collected in riverine forest at the base of the Taita Hills. This species was also found in the Kenyan highlands in Njuki-ini forest, once continuous with the great forests of Mount Kenya but now cut off because of population pressures. The Gwasi hills site is on the edge of Lake Victoria in western Kenya. The forests on the upper side and the top of the hill have been largely destroyed as a result of agricultural expansion. Finally, Marsabit mountain in northern Kenya is essentially a desert mountain, but as the only geological feature of any great elevation in the xeric north, any moisture that accompanies easterly winds tends to precipitate out over the mountain. Additional water comes from the frequent mists that cover the entire mountain and descend to the forest floor at certain times of the year. Within Kenya, then, *D.tricolor* has by far the widest geographic distribution of *Dinapsis* species, found from Taita Hills in the south to Marsabit Mountain in the north, and from the Kenyan coast in the east to the shores of Lake Victoria in the west. Its altitudinal distribution is also great, with specimens collected from near sea level to 1471 m (Njuki-ini Forest) and ca. 1500 m in the western Gwasi hills.

##### Etymology.

*Dinapsistricolor* is named for its distinctive tri-coloured antennae (Fig. 27C). Noun in apposition.

##### Barcode sequence for paratype specimens ICIPE 49121.

specimen code: 38754_A07_NMKE_Din_tric (sequence code in BOLD:FSA1899-21) BIN URI: None (sequence too short).

##### Nucleotide sequence for ICIPE 49121.

AATAGAATTAAGAGTCCCAGGTTCTTTTATTGGTAATGATCAAATTTATAATTCTATTGTGACTGCTCNTGCTTTTATTATAATTTTTTTTATAGTTATACCATTTATAATGGGGGGATTTGGAAATTGACTTCTTCCCTTAATATTAGGAGCTCCGGATATATCTTATCCTCGTCTAAATAATCTGAGATTTTGATTATTAATTCCTTCTTTAATATTTTTATTAATAAGATTTTATATTGGTAGAGGAACAGGAACAGGATGAACTAT.

##### Barcode sequence for paratype specimen NMSA-HYM-000546.

specimen code: 38754_A08_NMSA-HYM-000546 (sequence code in BOLD: FSA1900-21) BIN URI: None (sequence too short).

##### Nucleotide sequence for NMSA-HYM-000546.

TCTTTAAGATTATTAATTCGAGCTGAATTAGGAAGTCCAGGATCTTTAATTGGGGATGATCAAATTTATAATACTATTGTAACAGCTCATGCTTTTATTATAATTTTTTTTATAGTTATACCTATTATAATTGGGGGGTTTGGAAATTGATTAGTACCCCTAATATTAGGGGCCCCTGATATAGCATTCCCTCGAATAAATAATATAAGATTTTGACTTTTACCACCCTCTATTACTCTCTTAATCTCCAGAAGAATCGTAGAAAATGGGGCTGGAAC.

##### Description.

**Holotype female. *Body*** length 4.5 mm excluding ovipositor.

***Colour*.** Body mostly black to dark brown with sparse minute white setae. Antenna distinctively tri-coloured, with scape, pedicel, and F1-F2 orange; F3-F4, F5 basally, and F8–F12 dark brown; and pale brownish white flagellar band on F5 apically, all of F6, and F7. Mandible, wing venation, metasomal sternites, hypopygium apically, ovipositor and sheath dark red-brown. Fore and mid femora and tibiae red-brown to bright orange. Trochanters, trochantellus and coxae (except hind coxae which are dark brown to black basally), white, orange or yellow. Hind femur and tibia black to dark red-brown, apices may be orange-brown. Eyes and ocelli silvery. Wing membrane clear except two dark brown pigmented bands across forewing.

***Head*** round, 1.11 × wider than height; vertex, frons, and face evenly foveate-reticulate; ocelli small, OOL 1.80 × ocellar diameter; ocellar triangle equilateral; eye large and slightly protuberant, nearly parallel in anterior view; eye evenly covered with distinct ocular setae; eye margined posteriorly by coarsely foveate groove and two distinct postocular orbital carinae; antenna with 12 flagellomeres having flagellar length/width ratios as follows: F1 = 5.0, F2 = 4.0, F3 = 3.5, F10 = 2.5, F11 = 2.5, F12 (apical flagellomere) = 3.75; apical flagellomere not wider than basal flagellomeres; temple areolate-reticulate, temple width 1.25 × eye width in lateral view; gena areolate-reticulate medially and ventrally; malar space length 1.0 × mandible width basally; border of occipital carina coarsely foveate.

***Mesosoma*.** Pronotum punctate and finely setose medio-laterally, except laterally with coarsely foveate median depression and posteriorly with evenly foveate margin; mesoscutum as wide as long, mesoscutal lobes coarsely foveate, with sparse punctures and scattered erect setae, anterolateral corners lacking tubercles; median mesoscutal sulcus and scutoscutellar sulci finely and evenly foveate; transscutal articulation narrow, finely foveate; scutellar disc medially smooth and shining, with scattered punctures laterally; scutellar disc medially devoid of setae, laterally rimmed with small erect setae, and posteriorly with shorter, denser, depressed setae; axillae smooth with scattered punctures and sparse small erect setae; mesopleuron mostly rugo-punctate and evenly setose, anterior border foveate, disc with large median mid-pit, and distinctly depressed ventro-medially to conform to meso-femur shape; propodeum medially with weakly-developed transverse carinae between longitudinal carinae, submedian longitudinal carinae posteriorly diverging slightly towards middle of propodeum.

***Legs*.** Apex of fore tibia with comb of eight stout spines; hind coxa smooth to finely shagreened, weakly covered with long, silky, white setae not obscuring surface; hind femur stout, 2.2 × longer than wide, outer surface of hind femur sparsely but evenly covered with long, erect, silky white setae, inner surface of femur smooth, shining, and mostly devoid of setae; surface of hind tibia smooth, tibia dorsally, laterally, and ventrally with long, silky white setae, dorsal setae longer and black but hair-like, lacking spatulate tips; inner median margin of hind tibia with a dense longitudinal patch of shorter white setae; hind basitarsus long, distinctly longer than remaining four tarsomeres combined; basitarsus ventrally with dense preening brush consisting of numerous short, white setae, inclined posteriorly; basitarsus dorsally with normal hair-like setae, lacking spatulate tips; T2, T3, and T4 each shorter than preceding tarsomere, with T4 being the shortest; T2 ca. 1.5 × longer than T3, and ca. 2 × longer than T4; T2–T5 with short normal hair-like setae; T4 short and compact, length equal to width; tarsal claw simple, strongly curved.

***Wings*.** Forewing length 3.0 mm; wing covered with scattered setae, less densely setose basally, more densely and evenly setose apically; wing clear with two vertical darkly pigmented bands. Basal wing band narrowest, starting at basal corner of cell 1M, extending ventrally to cover most of cell 2CU and 3A; apical wing band wider, starting at base of pterostigma, densely covering entire marginal cell 2R1, extending apically well beyond marginal cell and diffusely approaching wing apex, ventrally covering entire cell 1+2RS, with pigmentation extending across cells 2+3M and 3CU, to lower wing margin; forewing venation with vein Rs apically curving abruptly towards anterior wing margin to form very short, truncate marginal cell 2R1; apical segment of vein M long, extending slightly beyond apex of marginal cell, vein M with small white bulla situated at mid length of vein. Hind wing with apical stub of vein Rs very short, equal to ½ shortest width between the propodeal submedian longitudinal carinae basally.

***Metasoma*** in dorsal view 2.9 × longer than wide, with seven dorsally visible terga, all smooth and shining dorsally, finely shagreened laterally; exposed portion of ovipositor, in lateral view, 1.62 × longer than metasoma length; ovipositor sheaths minutely setose, strongly curled (an artefact of preservation), appearing much shorter than ovipositor due to post-mortem curling.

##### Variation in paratype females.

Body length 3.4–5.5 mm. Forewing length 2.5–4.6.0 mm. Colour of F2 varies from dark brown to orange. Apical ½ to ¾ of F5 white. Ovipositor length varying from 1.6–2.0 × metasoma length. The ovipositor sheaths are normally as long as the ovipositor but may appear more or less much shorter due to post-mortem curling; however, one specimen has short sheaths that are < ¼ the ovipositor length (possibly a developmental abnormality, or as a result of damage). The Gai Hill specimen has more brightly coloured legs than the rest of the type series: fore leg coxa and trochanter very bright yellow, rest of leg bright orange; mid leg coxa orange/brown, trochanter bright yellow, rest of leg bright orange; hind leg coxa bright yellow except basally black, trochanter bright yellow, femur and tibia dark brown except the very apex of each orange/brown, tarsi light orange.

##### Variation in paratype males.

Body length 3.0–3.6 mm. Forewing length 2.4–2.6 mm. Antenna either entirely black, or with scape, pedicel, and F1-F2 dark brown, remainder of flagellomeres black without white band on F6-F7. Hind coxa mostly white, but black basally, or completely dark brown.

#### ﻿ Dinapsis turneri

Taxon classificationAnimaliaHymenopteraMegalyridae

Waterston, 1922

0040ED97-810B-5CBE-A840-B773E2D2632F

**Figure 33.**
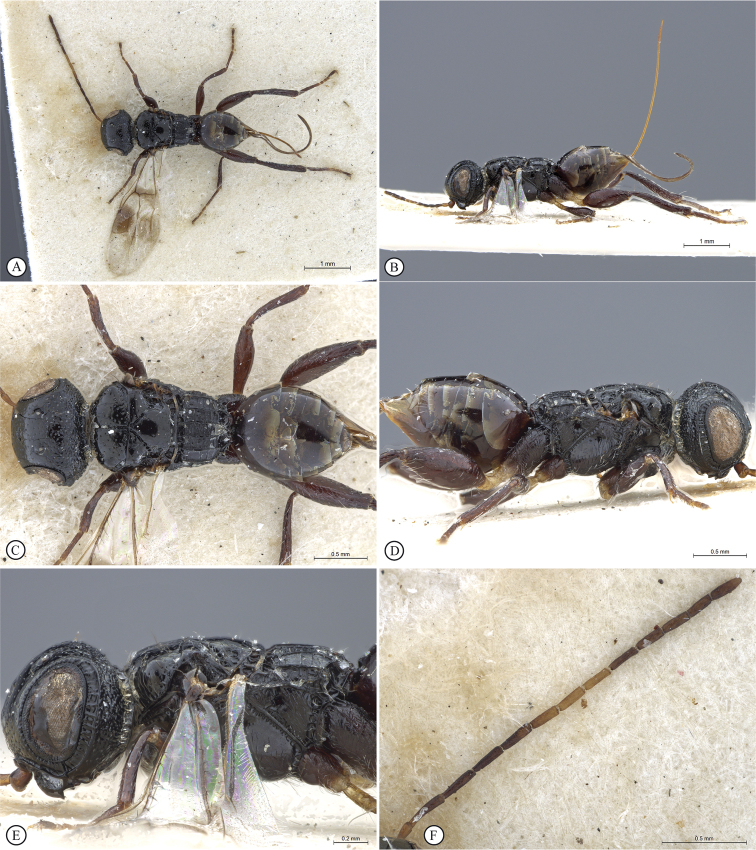
*Dinapsisturneri* holotype female NHMUK010198789 (NHMUK) **A** habitus, dorsal view **B** habitus, lateral view **C** head, mesosoma, metasoma, dorsal view **D** head, mesosoma, metasoma, lateral right view **E** head and mesosoma, lateral left view **F** right antenna, dorsal view. Scale bars: 1000 µm (**A, B**); 500 µm (**C, D, F**); 200 µm (**E**).

**Figure 34.**
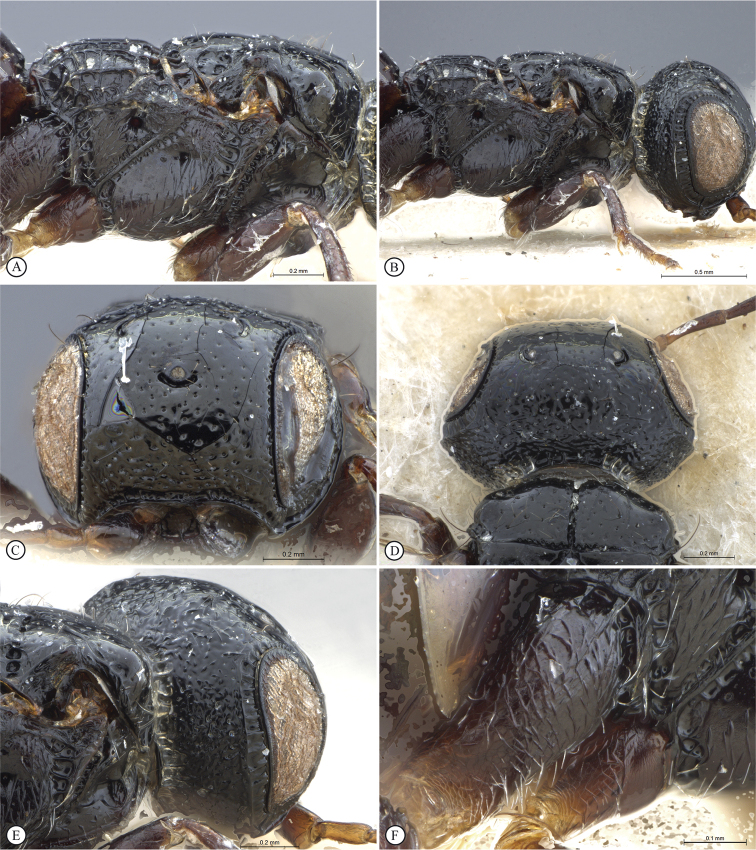
*Dinapsisturneri* holotype female NHMUK010198789 (NHMUK) **A** mesosoma, lateral right view **B** head, mesosoma, lateral right view **C** head, anterior view **D** head, mesoscutum, dorsal view **E** head, posterolateral view **F** hind coxa, antiaxial view. Scale bars: 200 µm (**A, C, D, E**); 500 µm (**B**); 100 µm (**F**).

**Figure 35.**
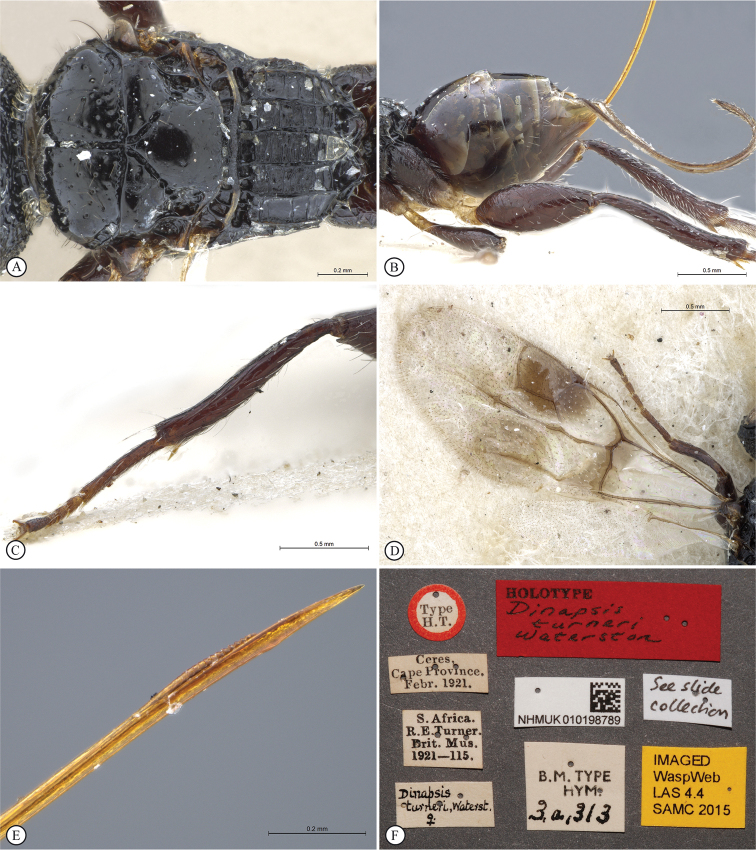
*Dinapsisturneri* holotype female NHMUK010198789 (NHMUK) **A** mesosoma, dorsal view **B** metasoma, legs, lateral view **C** hind leg, antiaxial view **D** wings, dorsal view **E** ovipositor tip, lateral view **F** data labels. Scale bars: 200 µm (**A, E**); 500 µm (**B–D**).

**Figure 36.**
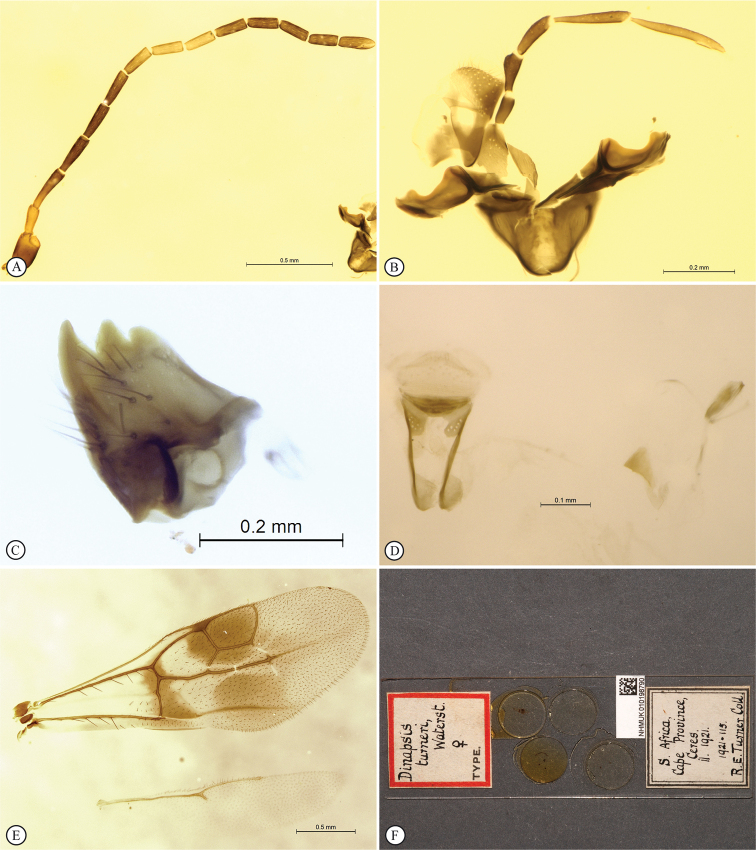
*Dinapsisturneri* holotype female slide-mount NHMUK010198789 (NHMUK) **A** antenna, lateral view **B** maxillolabial complex, dorsal view **C** mandible, posterior view **D** sitophore and pharyngeal plate on left, structure on the right not identifiable (possibly part of the maxillolabial complex) **E** wings, dorsal view **F** slide. Scale bars: 500 µm (**A, E**); 200 µm (**B, C**); 100 µm (**D**).

**Figure 37.**
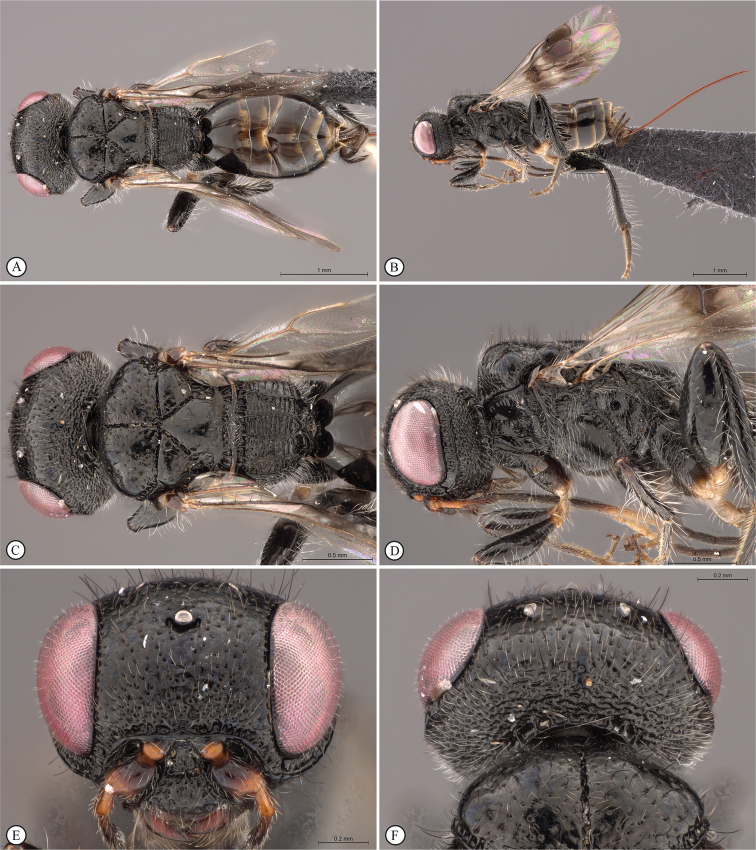
*Dinapsisturneri* non-type female, Asante Sana, SAM-HYM-P043549 (SAMC) **A** habitus, dorsal view **B** habitus, lateral view **C** head, mesosoma, dorsal view **D** head, mesosoma, lateral view **E** head, anterior view **F** head, dorsal view. Scale bars: 1000 µm (**A, B**); 500 µm (**C, D**); 200 µm (**E, F**).

**Figure 38.**
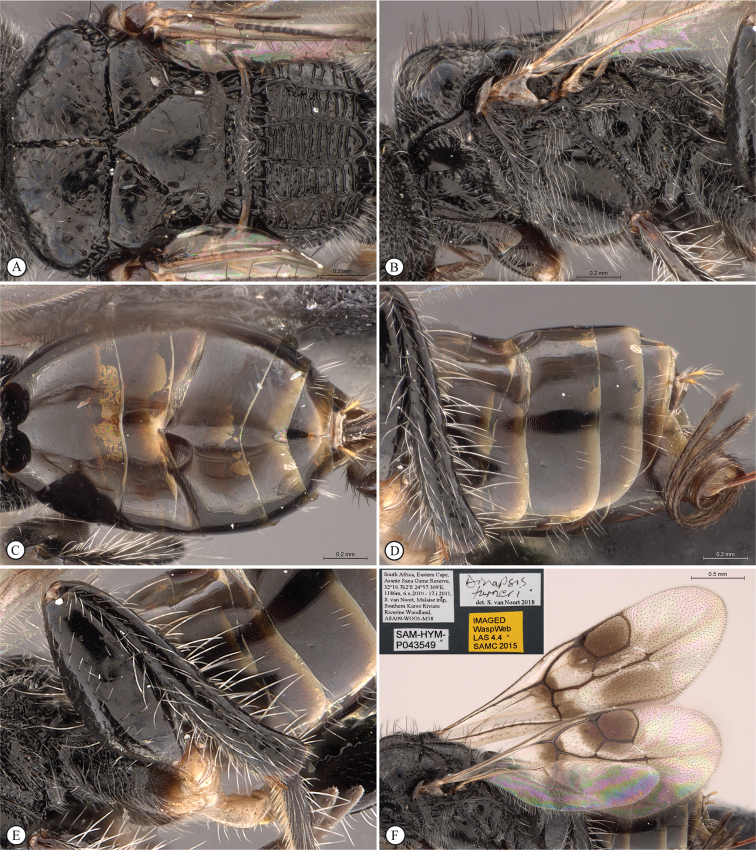
*Dinapsisturneri* non-type female, Asante Sana SAM-HYM-P043549 (SAMC) **A** mesosoma, dorsal view **B** mesosoma, lateral view **C** metasoma, dorsal view **D** metasoma, lateral view **E** hind leg, antiaxial view **F** wings, dorsal and ventral view (inset: data labels). Scale bars: 200 µm (**A–E**); 500 µm (**F**).

Figs 33 , 34 , 35 , 36 , 37 , 38

##### Material examined.

***Holotype***. South Africa • ♀; [Western Cape], Cape Province, Ceres; [33.358903°S, 19.298398°E]; Feb. 1921; R.E. Turner leg.; Brit. Mus.; 1921-115; *Dinapsisturneri*, Waterst. ♀; B.M. TYPE HYM 3,a,313, NHMUK 010198789; see slide collection; IMAGED WaspWeb LAS 4.4 SAMC 2015 [yellow label]; Type H.T. [white circular label with red rim]; HOLOTYPE *Dinapsisturneri* Waterston [red label]; NHMUK.

##### Additional material.

South Africa • 1 ♀; Eastern Cape, Asante Sana Game Reserve; 32°16.762'S, 24°57.309'E; 1186 m a.s.l.; 7 Apr. –28 Jul. 2010; S. van Noort leg.; Malaise trap; Southern Karoo Riviere Riverine Woodland; ASA09-WOO1-M10; SAM-HYM-P043547; SAMC • 1 ♀; same data except for 28 Jul. –6 Oct. 2010; ASA09-WOO1-M14; SAM-HYM-P043548; SAMC • 2 ♀♀; same data except for 6 Oct. 2010–17 Jan. 2011, ASA09-WOO1-M18; SAM-HYM-P043549; SAMC • 1 ♀; Eastern Cape, Huntly Glen Farm; 1065 m a.s.l.; 32°24.561'S, 25°05.946'E; 6 Oct. 2010–18 Jan. 2011; S. van Noort leg.; Yellow pan trap; Great Fish Thicket; HUN10-ACA1-Y05; SAM-HYM-P048022; SAMC • 1 ♀; Northern Cape, Avontuur Farm, 16 km NW Nieuwoudtville; 31°16.249'S, 19°02.900'E; 764 m a.s.l.; 28 Sep. –29 Dec. 2010; S. van Noort leg.; Malaise trap; Bokkeveld Sandstone Fynbos; GL07-FYN1-M152; SAM-HYM-P088316; SAMC • 1 ♀; Western Cape, Banghoek Valley, Dwarsriviershoek Farm; 33°56.824'S, 18°58.123'E; 400 m a.s.l.; 7 Oct. –Nov. 2014; S. van Noort leg.; Malaise trap; Mesic Mountain Fynbos; BH12-FYN6-M22; SAM-HYM-P048073; SAMC • 3 ♀♀; same data except for 24 Jun. –21 Sep. 2015; BH12-FYN6-M30; SAM-HYM-P088348; SAM-HYM-P088349; SAM-HYM-P088350; SAMC • 2 ♀♀; same data except for 21 Sep.–3 Nov. 2015; BH12-FYN6-M31; SAM-HYM-P086443; SAM-HYM-P088320; SAMC • 1 ♀; 1 ♂; same data except for 26 Jun. –29 Mar. 2016; BH12-FYN6-M34; SAM-HYM-P088351; SAM-HYM-P088352; SAMC.

##### Diagnosis.

Scutoscutellar sulci meet transscutal articulation independently. The propodeum is short (twice as wide as long; Fig. 33C). Forewing with narrow black bands, absent from costal cell; short, dark setae on dorsal surface of mesoscutum. Hind tibial dorsal setae large and white with fewer large black setae scattered between the white setae. Trochanter and trochantellus whitish yellow on all legs.

##### Distribution.

(Fig. 44) South Africa (Eastern Cape, Northern Cape, and Western Cape provinces).

##### Comments.

*Dinapsisturneri* is associated with the Fynbos biome in the Western Cape and the Savanna biome in the Eastern Cape and appears to not be a forest-associated species.

##### Revised description based on holotype and freshly collected specimens.

**Female. *Body*** length 3–4 mm excluding ovipositor.

***Colour*.** Head and mesosoma black. Head with a dense covering of white setae on occiput, brown, more widely spaced setae on face and frons (rubbed off on holotype); mesoscutal plate black, mesosoma black; metasoma dark brown. Scape and pedicel orange-brown, F1–F4 and F8–F12 dark brown, F5–F7 yellowish brown forming a median anellus on antennae. Trochanter and trochantellus whitish yellow, rest of legs black, except tarsi which are lighter. Ovipositor orange-brown; mandibles reddish brown. Eyes and ocelli silvery. Wing membrane clear except for two dark brown pigmented bands across forewing, apical band wide, basal band narrow ca. 1/3 width of apical band with the apical band extending slightly as a medial infuscation towards apical wing margin.

***Head*** oval, 1.25 × wider than high; vertex, frons, and face evenly sparsely punctate, interstices polished and 1–3 × greater than puncture width; ocelli small, OOL twice ocellar diameter; all ocelli bounded by a semi-circular depression on the side facing outer edge of the triangle; ocellar triangle an isosceles (LOL 1.25 × LOL); eye large and slightly protuberant, inner margins nearly parallel in anterior view, but diverging slightly ventrally; eye evenly covered with minute white ocular setae; eye margined posteriorly by foveate groove; postocular orbital carina absent; antenna with 12 flagellomeres having flagellar length/width ratios as follows: F1 = 6, F2 = 5.5, F3 = 5.0, F4 = 5.0, F5-F7 = 4.0, F8 = 4.5, F9 to F11 = 4.0, F12 = 4.5; apical flagellomere wider than basal flagellomeres; temple adjacent to ocular orbital carina punctate-rugulose, temple width 0.75 × eye width in lateral view; malar length 1.5 × mandible width basally; occiput punctate-rugulose in contrast to the polished, sparsely punctate vertex; occipital carina wide and crenulate with large fovea.

***Mesosoma*.** Pronotum medially polished, laterally excavated with a row of large oblong foveae situated dorsally, and posteriorly on the margin with the mesopleuron; medially with angled, central tri-radiating rows of foveae, dorsal two arms of fovea joining with dorsal row of fovea circumscribing a medial polished excavation. Mesoscutal anterior plate polished, with a medial row of punctures, and a lateral carina bounded by a shallow foveate groove; mesoscutum 1.18 × wider than long, mesoscutal shoulders evenly rounded, polished, with scattered long setae; mesoscutal lobes hardly evident; medial mesoscutal furrow deep and sinusoidally jagged with fovea; transscutal articulation a smooth furrow, anterior edge crenulated, posterior edge straight; scutoscutellar sulci comprising a line of adjacent large fovea, meeting transscutal articulation independently; scutellar disc medially polished, with scattered erect setae; mesopleuron in lateral view shallowly foveate on edges, except for posterior ventral margin, medially polished with dense long, white setae covering most of mesopleuron, except for glabrous posterior patch; with large median pit. Metanotum with raised, setose medial area flanked anteriorly by narrow foveate depression laterally grading into wider, larger foveae. Propodeum medially with strongly developed transverse carinae between submedian longitudinal carinae, fewer transverse carinae present in lateral longitudinal tracks. Medial track anteriorly with two deep fovea, lateral tracks each anteriorly with two or three deep foveae.

***Legs*.** Apex of fore tibia with comb of stout spines; hind coxa sparsely punctate, densely covered with long, silky, white setae obscuring surface on ventral 1/2; hind femur stout, polished, 2.8 × longer than wide, outer surface of hind femur sparsely, but evenly covered with short, erect, white setae, inner surface of hind femur polished, sparsely punctate with very short setae; surface of elongate (7.5 × longer than wide) hind tibia polished, with long erect white setae dorsally and fewer large black setae scattered between the white setae, shorter setae laterally and ventrally; dorsal setae lacking spatulate tips; inner ventral margin of hind tibia with a dense longitudinal patch of shorter whitish yellow setae; hind basitarsus long, subequal in length to remaining four tarsomeres combined; basitarsus ventrally with dense preening brush consisting of numerous short, whitish yellow setae, inclined anteriorly; basitarsus dorsally with normal scattered long, white setae, lacking spatulate tips; T2 and T3 1.3 × as long as wide, T4 as long as wide, T5 4 × as long as wide; all tarsomeres with normal hair-like setae; tarsal claw simple, strongly curved.

***Wings*.** Forewing length 2.9 mm, 2.9 × longer than wide; wing basally with cells R and 1A largely devoid of setae; 1R1, 1M and 2CU with very small, sparse setae compared to wing apical of these cells, which is evenly covered with small, scattered setae; wing clear with two dark pigmented vertical bands. Basal wing band much narrower than apical band, narrowest dorsally widening progressively towards ventral wing margin, covering basal 1/2 of cell 1M, anterior eighth of cells R and 1Cu, extending ventrally to wing margin, covering entire cell 2CU and 3A; apical wing band wider, starting at base of pterostigma, and anterior 1/3 of 1R1, extending apically to cover entire marginal cell 2R1, ventrally to cover entire cell 1+2RS, medially more diffuse, slightly extending towards apical wing margin; extending ventrally with infuscate pigmentation across cells 2+3M and 3CU, to lower wing margin; forewing venation with vein Rs apically curving abruptly towards anterior wing margin to form short, truncate marginal cell 2R1; apical segment of vein M long, extending beyond apex of marginal cell, vein M with small white bulla situated at 0.425× vein length. Hind wing with apical stub of vein Rs 2/3 of shortest width between the propodeal submedian longitudinal carinae.

***Metasoma*** in dorsal view 1.57 × longer than wide, with seven dorsally visible terga, all polished; pygostyles long, setose, pale yellow; exposed portion of ovipositor, in lateral view 2.0 × longer than metasomal length; ovipositor sheaths setose, strongly curled (an artefact of preservation).

##### Variation.

Male much smaller than female, 2.0 mm body length; flagellum uniformly yellowish brown without medial lighter coloured band section.

#### ﻿ Dinapsis zulu

Taxon classificationAnimaliaHymenopteraMegalyridae

Shaw & van Noort sp. nov.

8B81E60F-3343-5854-976D-85DCBA6CEB3B

https://zoobank.org/9AAEE139-2209-4082-911A-7B79943C0F29

**Figure 39.**
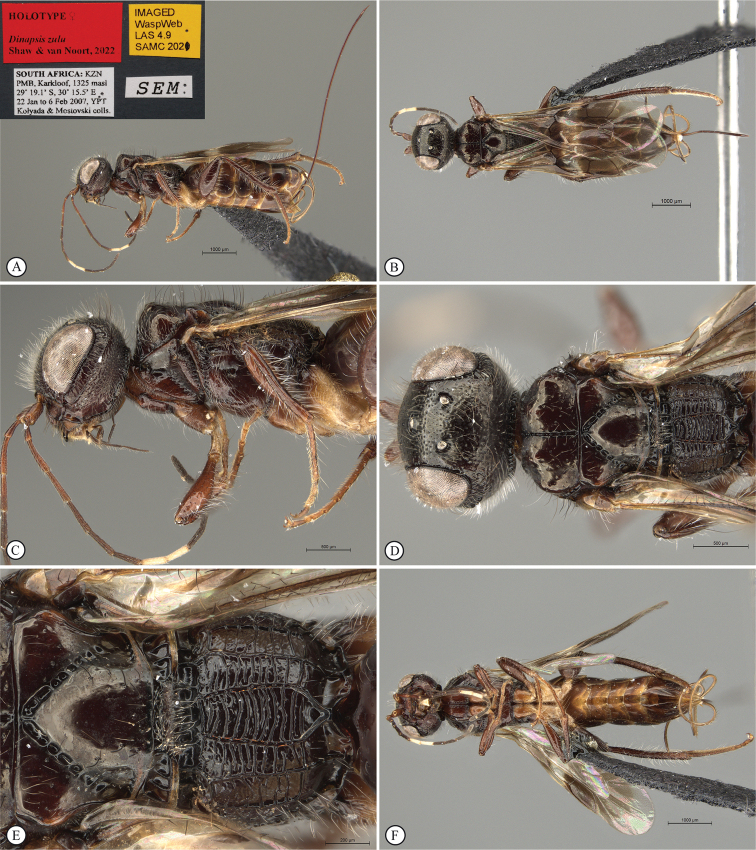
*Dinapsiszulu* Shaw & van Noort, sp. nov. holotype female (NMSA) **A** habitus, lateral view (inset: data labels) **B** habitus, dorsal view **C** head, mesosoma, lateral view **D** head, mesosoma, dorsal view **E** mesoscutellum, propodeum, dorsal view **F** habitus, ventral view. Scale bars: 1000 µm (**A, B, F**); 500 µm (**C, D**); 200 µm (**E**).

**Figure 40.**
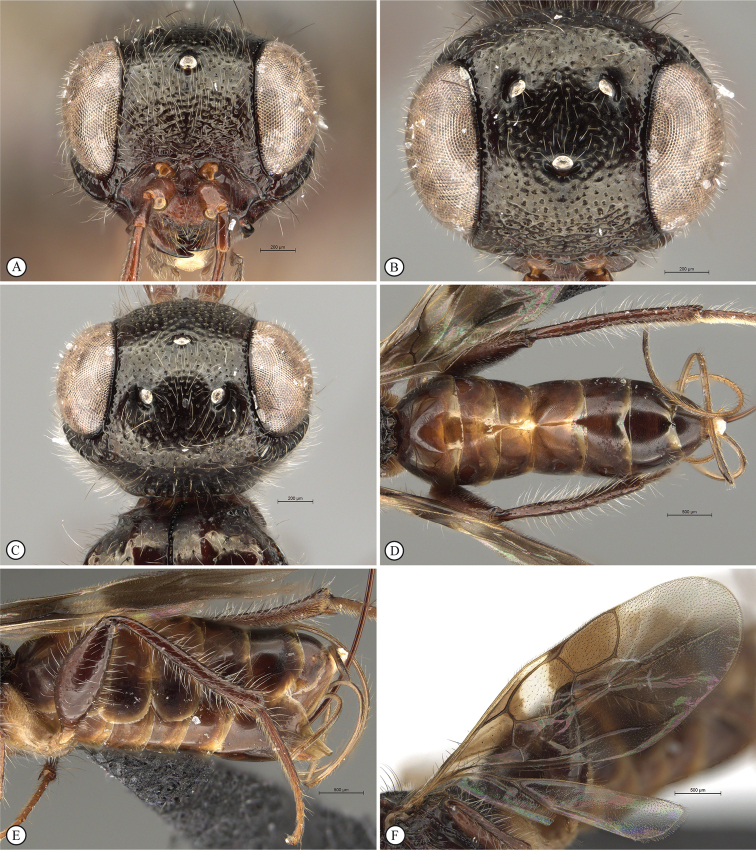
*Dinapsiszulu* Shaw & van Noort, sp. nov. holotype female (NMSA) **A** head, anterior view **B** head, dorsal view **C** head, mesoscutum, dorsal view **D** metasoma, dorsal view **E** metasoma, hind leg lateral view **F** wings, dorsal view. Scale bars: 200 µm (**A–C**); 500 µm (**D–F**).

**Figure 41.**
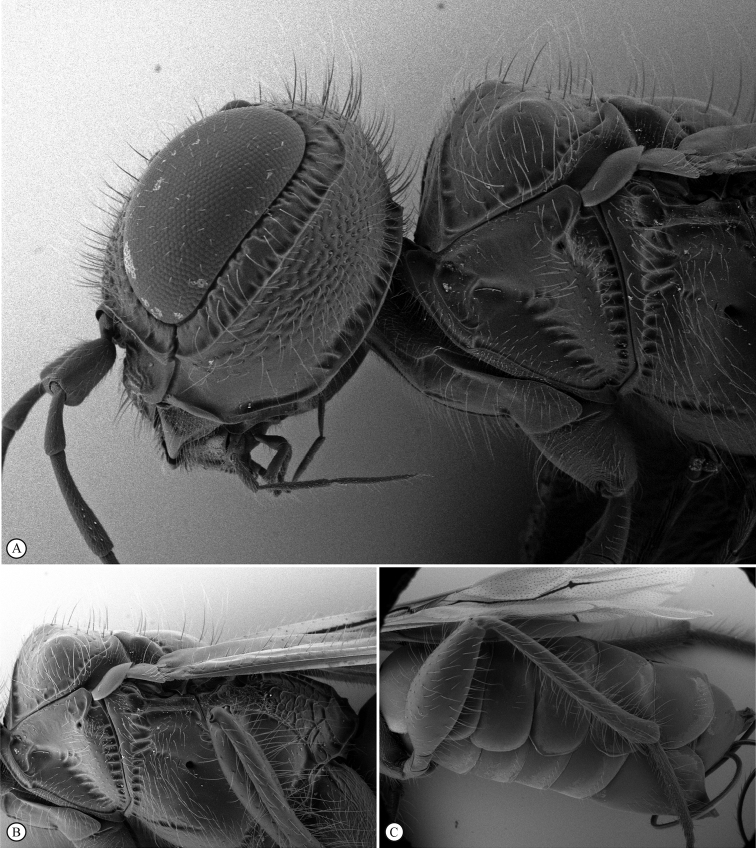
*Dinapsiszulu* Shaw & van Noort, sp. nov. holotype female, SEM (NMSA) **A** head, mesonotum, pronotum, lateral view **B** mesosoma, lateral view **C** metasoma, lateral view.

**Figure 42.**
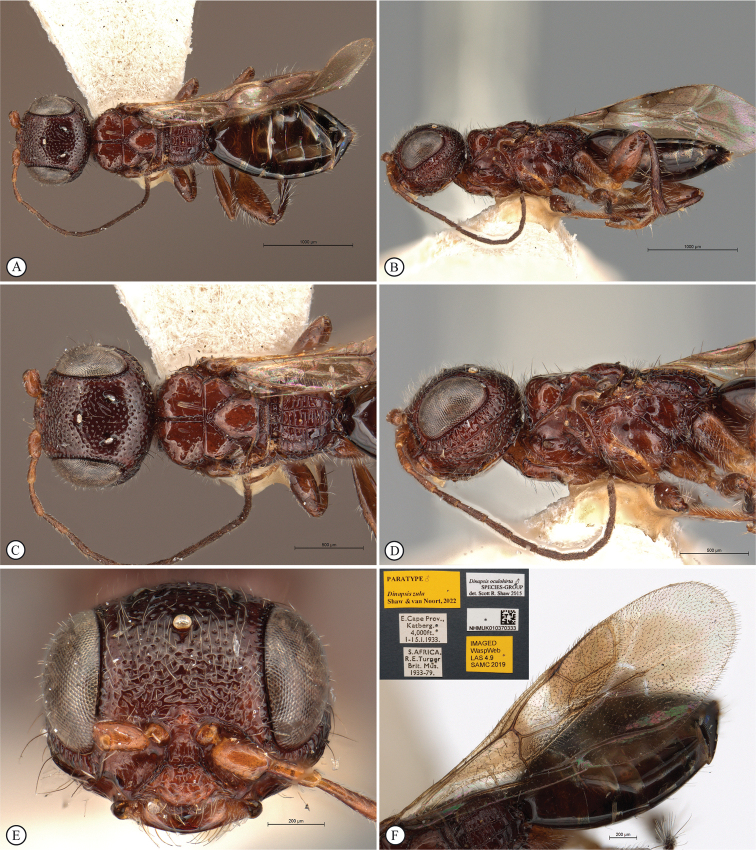
*Dinapsiszulu* Shaw & van Noort, sp. nov. paratype male NHMUK010370333 (NHMUK) **A** habitus, dorsal view **B** habitus, lateral view **C** head, mesosoma, dorsal view **D** head, mesosoma lateral view **E** head, anterior view **F** wings, dorsal view (inset: data labels). Scale bars: 1000 µm (**A, B**); 500 µm (**C, D**); 200 µm (**E, F**).

Figs 39 , 40 , 41 , 42

##### Material examined.

***Holotype*.** South Africa • ♀; KwaZulu-Natal, PMB, Karkloof, 1325 m a.s.l., 29°19.1'S, 30°15.5'E, 22 Jan.–6 Feb. 2007; YPT (yellow pan trap); Kolyada & Mostovski leg.; NMSA-HYM002031; NMSA. ***Paratypes*.** South Africa • 7 ♀♀ 1 ♂; same data as holotype; NMSA-HYM000536 to 543; NMSA • 1 ♂; same data as holotype, except for 22 Sep.–3 Oct. 2005; M. Mostovski leg.; NMSA-HYM000535; NMSA • 1 ♀; Cathedral Peak N.R., Rainbow Gorge; 1480 m a.s.l.; 28°57.60'S, 29°13.61'E; 22 Sep.–17 Nov. 2006; MT [Malaise trap]; M. Mostovski leg.; NMSA • 1 ♀; Karkloof [29.297268°S, 30.231318°E]; 48 km NE Pietermaritzburg; mist forest; 27 Nov.–11 Dec. 1991; A. Mitchell leg.; Malaise trap; NMSA • 2 ♂♂; Eastern Cape, Katberg [32.473670°S, 26.670881°E]; 4,000 ft a.s.l.; 1–15 Jan. 1933; R.E. Turner leg.; Brit. Mus. 1933–1979; *Dinapsisoculohirta* species group, det. Scott R. Shaw 2015; NHMUK010370333; NHMUK010370327; NHMUK • 4 ♀♀ 1 ♂; Cape Province, Tsitsikama Coastal N.P.; 34°02'S, 23°53'E; Jan. 1996; Malaise trap; Michael Söderlund leg.; MZLU • 1 ♀; Cape Province, Tsitsikamma National Park; 34°02'S, 23°53'E; 14–18 Dec 1994; loc. 23; R. Danielsson leg.; MZLU.

##### Diagnosis.

In the key to African *Dinapsis* species by Hedqvist (1967), *D.zulu* keys to couplet 2 because of the presence of minute ocular setae, a characteristic shared with *Dinapsisoculohirta* Hedqvist and some new species treated in this paper. *Dinapsisoculohirta* is a much smaller species (females < 3 mm) that is only known to occur in Madagascar, so should not be confused with this new species. More characters for *D.oculohirta* are discussed in the diagnosis section for *D.tricolor* from Kenya. *Dinapsiszulu* is easily distinguished from other *Dinapsis* species by its large body size, very tall and erect setae dorsally on the head vertex and metanotum (Figs 39C, 41A) (shared only with *D.gamka*), gena smooth and punctate (lacking rugose sculpture), only one postocular carina, and elongate metasoma. Females have a long ovipositor and a pale-coloured band on F6-F7. *Dinapsiszulu* can be distinguished from *D.gamka* by the head height being subequal to the mesosoma height (the head is 1.5× taller than the mesosoma in *D.gamka*), and *D.zulu* has a wider postocular furrow behind the eye (as in Fig. 41A).

##### Distribution.

(Fig. 44) South Africa (Eastern Cape and KwaZulu-Natal provinces).

##### Comments.

Based on currently known data, *Dinapsiszulu* is associated with the forest biome, which is a naturally highly fragmented habitat in South Africa (Lawes 1990, 2004; Eeley et al. 1999; Mucina and Geldenhuys 2006; Lawes et al. 2007; Deng et al. 2020), and hence the species distribution is likely to follow suit. The current distribution pattern is likely to be an artefact of under-sampling and we expect the species to be recorded from further forest localities in both provinces. While the main body colour is black to dark brown in most specimens, the specimens from Tsitsikamma have lighter tones ranging from reddish brown to yellowish brown. This might be due to different environmental conditions (the Tsitsikamma forest is more temperate) during their development, or they might be specimens that were collected soon after emergence before the cuticle had fully tanned and darkened.

##### Etymology.

*Dinapsiszulu* is named after the Zululand region encompassing the type locality and the Zulu people. Noun in apposition.

##### Barcode sequences for paratype specimen.

38754_A09_NMSA-HYM-000539 (sequence code in BOLD: FSA1901-21) BIN URI: None (sequence too short).

##### Nucleotide sequence.

GCTCATGCTTTTATTATAATTTTTTTTATAGTTATACCTTTTATAATGGGAGGTTTTGGAAATTGATTATTACCTTTAATATTAGGNGCNCCTGATATATCTTACCCCCGAATAAATAATTTAAGATTTTGATTATTA

##### Description.

**Holotype female. *Body*** length 6.8 mm excluding ovipositor.

***Colour*.** Body mostly black to dark brown with sparse minute white setae. Scape, pedicel, F1–F5, mandible, most of legs, wing venation, metasomal sternites, hypopygium apically, ovipositor and sheath dark reddish brown. F6-F7, trochanters, and hind coxa apically pale yellowish white. Eyes and ocelli silvery. Wing membrane clear except two dark brown pigmented bands across forewing.

***Head*** round, 1.11 × wider than height; vertex, frons, and face shallowly and evenly punctate; ocelli small, OOL 1.8 × ocellar diameter; ocellar triangle equilateral; eye large and slightly protuberant, nearly parallel in anterior view; eye lightly and evenly covered with minute ocular setae; eye margined posteriorly by irregular and shallowly foveate groove and one distinct postocular orbital carina; second postocular orbital carina entirely absent; antenna with 12 flagellomeres having flagellar length/width ratios as follows: F1 = 5.6, F2 = 6.6, F3 = 6.6, F10 = 4.0, F11 = 4.0, F12 = 3.25; apical flagellomere slightly wider than basal flagellomeres; temple punctate, temple width 1.3 × eye width in lateral view; gena punctate medially, smooth ventrally; malar space length 1.5 × mandible width basally; border of occipital carina evenly foveate.

***Mesosoma*.** Pronotum smooth, except laterally with foveate median depression; mesoscutum as long as wide, mesoscutal lobes smooth, with sparse punctures and scattered tall setae, antero-lateral corners lacking tubercles; median mesoscutal sulcus and axillular grooves narrowly and evenly foveate; scutellar furrow narrow and smooth; scutellar disc medially smooth and shining, with scattered punctures laterally; scutellar disc medially devoid of setae, laterally rimmed with large erect setae, and posteriorly with shorter, denser, depressed setae; axillae smooth with scattered punctures and sparse large erect setae; mesopleuron mostly smooth and lightly setose, medially, anterior border foveate, disc with large median mid-pit, and strongly depressed ventro-medially to conform to meso-femur shape; propodeum medially with well-developed transverse carinae between longitudinal carinae, submedian longitudinal carinae diverging posteriorly towards middle of propodeum.

***Legs*.** Apex of fore tibia with comb of eight stout spines; hind coxa smooth to finely shagreened, weakly covered with long, silky, white setae not obscuring surface; hind femur stout, 2.5 × longer than wide, outer surface of hind femur sparsely but evenly covered with long, erect, silky white setae, inner surface of femur smooth, shining, and mostly devoid of setae; surface of hind tibia smooth, tibia dorsally, laterally, and ventrally with long, silky white setae, dorsal setae longer and black but hair-like, lacking spatulate tips; inner median margin of hind tibia lacking a dense longitudinal patch of shorter white setae; hind basitarsus long, distinctly longer than remaining four tarsomeres combined; basitarsus ventrally with dense preening brush consisting of numerous short, white setae, inclined posteriorly; basitarsus dorsally with normal hair-like setae, lacking spatulate tips; T2 and T3 each short and compact, each ca. twice as long as wide; T2 and T3 with normal hair-like setae; T4 extremely short, distinctly shorter than wide; tarsal claw simple, strongly curved.

***Wings*.** Forewing length 4.4 mm; wing covered with scattered setae, less densely setose basally, more densely and evenly setose apically; wing clear with two dark pigmented vertical bands. Basal wing band narrowest, starting at basal corner of cell 1M, extending ventrally to cover most of cell 2CU and 3A; apical wing band wider, starting at base of pterostigma, densely covering entire marginal cell 2R1, extending apically well beyond marginal cell and diffusely approaching wing apex, posteriorly covering entire cell 1+2RS, with pigmentation extending across cells 2+3M and 3CU, to lower wing margin; forewing venation with vein Rs apically curving abruptly towards anterior wing margin to form very short, truncate marginal cell 2R1; apical segment of vein M long, extending well beyond apex of marginal cell, vein M with small white bulla situated at mid length of vein. Hind wing with apical stub of vein Rs very short, equal to ½ shortest width between the propodeal submedian longitudinal carinae basally.

***Metasoma*** in dorsal view 3.1 × longer than wide, with seven dorsally visible terga, all smooth and shining dorsally, finely shagreened laterally; exposed portion of ovipositor, in lateral view, 1.65 × longer than metasoma length; ovipositor sheaths minutely setose, strongly curled (an artefact of preservation), appearing much shorter than ovipositor due to post-mortem curling.

##### Variation in paratype females.

Body length 3.8–6.8 mm. Forewing length 3.0–4.4 mm. Pale band on F6-F7 varying from light yellowish white to light brown. Hind coxa varying from mostly dark brown or black to mostly yellowish white, but always dark brown to black basally and yellowish white apically. Ovipositor length varying from 1.6–2.0 × metasoma length. Head colour varying from black to reddish brown. Metasoma colour varying from black to light reddish or yellowish brown.

##### Variation in paratype male.

Body length 3.0 mm. Forewing length 2.4 mm. Antenna entirely dark brown, without a yellowish white band on F6-F7. Hind coxa entirely dark brown to black, lacking pale colour basally.

## ﻿Discussion

The family Megalyridae probably first evolved during the late Triassic period in tropical areas of the fragmenting supercontinent Pangaea, but no dated phylogeny has been produced yet. Subsequent evolution of megalyrid lineages has been shaped by the processes of rifting and continental drift among the southern continents (Shaw 1990b). The ancestors of the modern genus *Dinapsis* were probably isolated in the African region and Madagascar during the late Mesozoic era. The sister group of living dinapsines (species of *Neodinapsis* Shaw) was isolated and is now restricted to northern Chile (Shaw 1987). The sister group of *Dinapsis*, the genus *Ettchellsia*, was isolated in southeast Asia and is now known only from Thailand, Vietnam, Borneo, Java, China, and the Philippines (Baltazar 1962; Shaw 1990b; Mita and Shaw 2012). *Dinapsis* populations were divided after Madagascar rifted from the African continent, leaving species of the most basal species group (*D.oculohirta* species group) separated in southern Africa (Shaw and van Noort 2009). In Madagascar, three unique *Dinapsis* species groups (the *hirtipes* group, the *seyrigi* group, and the *nubilis* group) have evolved on that island and have not colonised the African mainland. This is consistent with an ancient age for the genus *Dinapsis* and an early separation of Madagascar from the African mainland during the Mesozoic. The extant species on the African mainland are probably not a monophyletic lineage but rather a paraphyletic assemblage of relatively basal species. The species from Madagascar, on the other hand, include examples of all the known *Dinapsis* species groups and may represent an ancient monophyletic lineage within the genus, however, too many Malagasy species are still rare or undescribed, so this issue is yet to be resolved.

**Figure 43.**
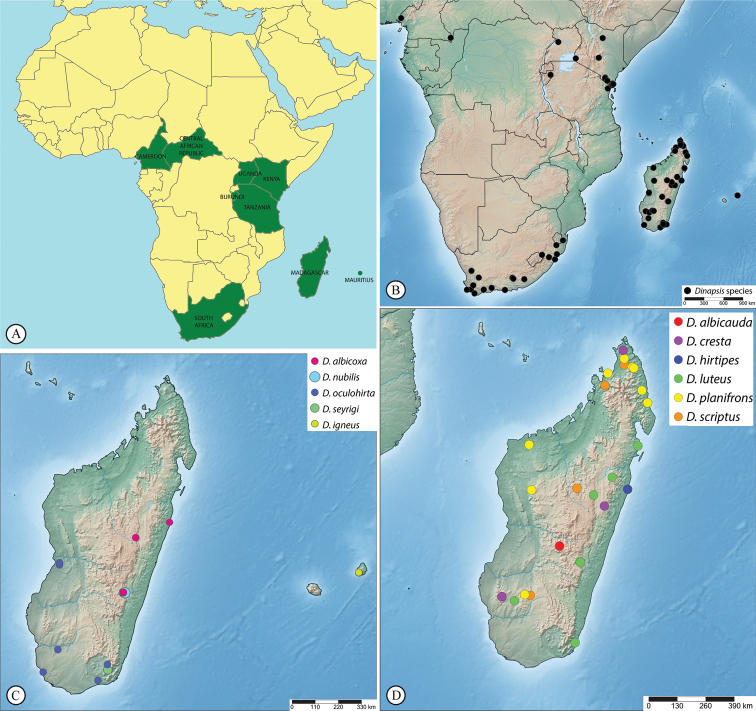
Distribution maps of *Dinapsis* species **A** country occurrences for the genus *Dinapsis***B** specimen point data of described *Dinapsis* species plotted on a topographical relief map **C***Dinapsis* species occurring in Madagascar and Mauritius, excluding the *D.hirtipes* species-group (specimen point data plotted on a topographical relief map) **D** Madagascan records of *Dinapsis* species in the *D.hirtipes* species group (specimen point data plotted on a topographical relief map).

Modern megalyrid species live in southern temperate forests of Australia (Shaw 1990a), Fynbos and Succulent Karoo of South Africa (Waterston 1922; Hedqvist 1959; Shaw 1988; van Noort and Shaw 2009), forests of East and Central Africa (Shaw and van Noort 2009), India (Binoy et al. 2020), and Chile (Shaw 1987). According to Kawada et al. (2014), some of the neotropical species of *Cryptalyra* Shaw, 1987 have been collected in rain forests, while others were collected in savannas (places much drier than rain forest, although there is a defined wet season). Mita et al. (2007) described *Carminator* Shaw, 1988 species from temperate broadleaf forests of Japan, which is both the northernmost distribution record for any modern extant megalyrid species, and also a unique habitat association for the family Megalyridae (at least for extant species). Another peak of diversity appears to be in the relict forests of Madagascar (Hedqvist 1967; Shaw pers. comm.). The most abundant and species-rich megalyrids (based on named species) appear to be the Australian species (Shaw 1990a), apparently because they have adapted to dry conditions of *Eucalyptus* woodlands and *Acacia* scrub habitats (Froggatt 1906; Fahringer 1928; Shaw 1990a). However, it is estimated that at least 20 new species remain to be described from the Madagascar *Dinapsis* fauna (Shaw pers. comm.). This suggests that eventually the Afrotropical dinapsine megalyrid fauna (including that of Madagascar) will be demonstrated to be even more species-rich than the megalyrid fauna of the Australian region. This seems especially likely since Australia has been comparatively better explored and the discovery of new species there is less likely, whereas the megalyrid-rich forests of Madagascar remain remote, little studied, and difficult to access. These forests are disappearing at an alarming rate due to anthropogenic transformation (Harper et al. 2007; McConnell and Kull 2014), with the result that some of the Madagascan *Dinapsis* species will probably be extinct before being discovered.

**Figure 44.**
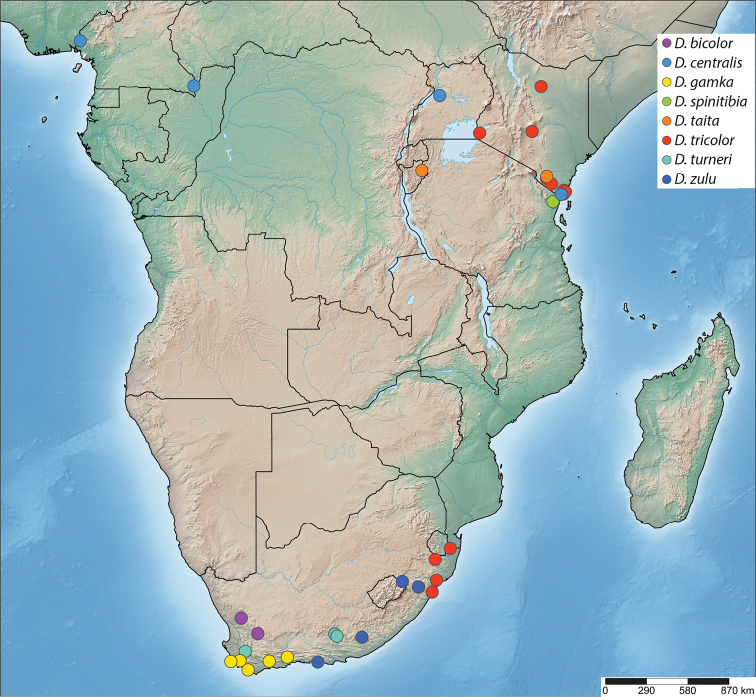
Distribution map of *Dinapsis* species occurring on the African mainland (specimen point data plotted on a topographical relief map).

Most of the *Dinapsis* species are associated with forest, but in South Africa a couple of species (*D.bicolor* and *D.gamka*) are associated with the Fynbos and Succulent Karoo biomes, collectively forming the Greater Cape Floristic Region (Born et al. 2007), and *D.turneri* is associated with Fynbos in the Western Cape Province and the Savanna biome in the Eastern Cape Province. At some localities in South Africa, e.g., Banghoek Valley near Stellenbosch (Western Cape Province), three species of Megalyridae co-occur: *Megalyridiacapensis* Hedqvist, 1959, *Dinapsisgamka*, and *D.turneri*, but this is based on deficient baseline data so these species’ co-occurrences may be more widespread than current data suggest. These three species have an overall distribution largely concordant with the Greater Cape Floristic Region encompassing the Western Cape, as well as part of the Northern and Eastern Cape provinces. They are associated with a range of habitat types including Fynbos and the arid Succulent Karoo (in the Western Cape and Northern Cape) and Savanna (in the Eastern Cape) suggesting that climate of the region is the potential delimiting factor (possibly acting on distributional range restriction of the host insect), rather than vegetation type for the distribution of these taxa. The remaining *Dinapsis* species are associated with various forest types in Africa, which have a range of ages. Afrotemperate forests (Afromontane of White 1981, 1983) are reasonably old having persisted since the Miocene (von Maltitz et al. 2003, White 1981), whereas the Indian Ocean Coastal belt forests are far younger, having been established approximately 8 000 years ago (Macdevette et al. 1989). The older Afrotemperate forests have been subjected to considerable expansion and contraction, particularly during the glacial maxima of the Pliocene and Pleistocene, resulting in climate and habitat driven speciation and extinction processes in these forests (Maley 1996; Eeley et al. 1999; Morley 2000; Lawes et al. 2007), Species (*D.spinitibia*, *D.taita*, *D.tricolor*, *D.zulu*) that have their distribution within the East African savanna are associated with the Afrotemperate forest fragments occurring at higher elevation within this biome, or with the Indian Ocean Coastal belt forests occurring at lower elevation. Lowland rainforest is far older than the other forest types but has also been considerably impacted by fragmentation over the last 36 mya because of the numerous ice ages that occurred during this period (Maley 1996; Morley 2000) suggesting that the central African species *D.centralis* that is associated with this habitat may be a basal species within the genus. No species appears to be associated with savanna grassland *per se* with the records of *D.turneri* from the savanna region in the eastern Cape being from riverine woodland. *Dinapsiszulu* is associated with the highly fragmented forest habitat present in the Savanna biome in South Africa (Lawes 1990, 2004; Eeley et al. 1999; Mucina and Geldenhuys 2006; Lawes et al. 2007), and hence the distribution of these species is likely to also be extremely localised and restricted to forest patches within the larger Savanna biome.

Although the Fynbos biome has elements that are ancient, particularly those in the families Bruniaceae and Proteaceae, diversity of this biome only escalated ca. 5 mya (Linder 2003). *Megalyridiacapensis*, which is strongly associated with Fynbos (van Noort and Shaw 2009), is a basal lineage (Shaw 1990b) within the evolution of the Megalyridae. *Dinapsis*, in contrast, is a much more recently derived genus (Shaw 1990b) whose ancestor may have adapted to the forest biome with some more recent switching back from Forest to the Fynbos and Succulent Karoo biomes. This does, however, require a phylogenetic analysis of the *Dinapsis* species to determine the evolutionary relationships within the genus, particularly of the non-forest associated species *D.bicolor* and *D.gamka*. This is preliminary conjecture, and these hypotheses require a thorough phylogeographical analysis to tease out the historical processes that have led to the contemporary distribution and habitat affinities of Megalyridae in Africa.

Field observations suggest that megalyrid wasps simply insert their ovipositor into pre-existing cavities, frass-filled holes, or cracks, rather than drilling into solid wood (Hacker 1913; Rodd 1951; Mesaglio and Shaw 2022). This “hole-poking” behaviour may provide a simple explanation for the transition from beetle larvae to wasp larvae in mud nests: the megalyrids simply poke their ovipositors into a different kind of hole, wasp nest entrances rather than frass-filled beetle galleries. These biological observations are limited to a few Australian species with comparatively longer ovipositors. Many of the Neotropical, Afrotropical, and Oriental species, especially those in the genera *Carminator*, *Cryptalyra*, *Dinapsis*, and *Ettchellsia*, have much shorter ovipositors. It seems unlikely that these species could either be attacking hosts deep in plant tissue or concealed inside nests. More likely they are attacking host insects very near the surface of plants or hosts that are not deeply concealed. Recent sampling in Madagascan primary forests and on the African continent has recovered *Dinapsis* specimens from Malaise traps, yellow pan traps, tree fogging and leaf litter sifting. This suggests that these insects are active near ground level as well as (sometimes) in the forest canopy (as is suggested by *D.centralis* and *D.spinitibia* having been obtained by tree fogging). The host associations of *Dinapsis* species could potentially be discovered by careful observation of insect activity in the forest undergrowth and leaf litter where much dead wood is available for potential xylophagous hosts. Given the rather small size of African *Dinapsis* species (as compared with Australian *Megalyra*, for example), they might possibly be attacking small beetle larvae in small stems, leaf petioles, seeds, or other aspects of ground-level leaf litter and plant debris.

The currently recorded distribution for the genus *Dinapsis* is a highly biased artefact due to undersampling. The dominance of records from Kenya, Madagascar, and South Africa are a result of more recent, focused, long-term inventory survey efforts conducted over the last 30 years by SvN (SAMC); Robert Copeland (ICIPE), and Brian Fisher and colleagues (CAS). Many further species are expected to be discovered from the large areas of the Afrotropical region currently without distribution records. *Dinapsis* species are rarely collected by hand net, and hence specimens are underrepresented in historical museum collections. Use of a variety of collecting methods in inventory surveys is important to adequately sample the faunal assemblage in any given habitat. Different methods target different species (Boulinier et al. 1998) and species complementarity between methods is usually low with high turnover of species across different methods (van Noort 2004). Malaise traps and yellow pan traps are particularly successful in returning megalyrid specimens, especially if deployed adjacent to dead branches of trees and shrubs. Malaise trap return of parasitoid wasps attacking xylophagous hosts is enhanced through the process of stacking dead branches and leaf litter from the surrounding vicinity on the ground within the trap itself, increasing capture of these wasps when they emerge from the plant material (van Noort et al. 2021) as supported by the return of *Dinapsis* specimens because of this practice during the inventory surveys conducted in South Africa. Continued deployment of rigorous, quantified inventory surveys, using a variety of collecting methods, in the largely unsampled habitats of Africa will undoubtably produce many further new species of *Dinapsis*. The revisionary treatment provided in this paper will hopefully provide a resource that will facilitate the ongoing exploration and documentation of African megalyrids.

## Supplementary Material

XML Treatment for Dinapsis

XML Treatment for Dinapsis albicauda

XML Treatment for Dinapsis albicoxa

XML Treatment for Dinapsis bicolor

XML Treatment for Dinapsis centralis

XML Treatment for Dinapsis cresta

XML Treatment for Dinapsis gamka

XML Treatment for Dinapsis hirtipes

XML Treatment for Dinapsis igneus

XML Treatment for Dinapsis luteus

XML Treatment for Dinapsis nubilis

XML Treatment for Dinapsis oculohirta

XML Treatment for Dinapsis planifrons

XML Treatment for Dinapsis scriptus

XML Treatment for Dinapsis seyrigi

XML Treatment for Dinapsis spinitibia

XML Treatment for Dinapsis taita

XML Treatment for Dinapsis tricolor

XML Treatment for Dinapsis turneri

XML Treatment for Dinapsis zulu

## Supplementary materials

Supplementary material 1 Appendix 1 Data type (measurement/occurrence/multimedia/etc.) Explanation note Lucid Interchange Format version 3 (LIF3) of the WaspWeb online Lucid matrix identification key to the Afrotropical species of Megalyridae (Hymenoptera).The LIF3 file is an XML-based file that stores all the Lucid3 key data, allowing exchange of the key with other key developers. https://binary.pensoft.net/file/714062This dataset is made available under the Open Database License (http://opendatacommons.org/licenses/odbl/1.0/). The Open Database License (ODbL) is a license agreement intended to allow users to freely share, modify, and use this Dataset while maintaining this same freedom for others, provided that the original source and author(s) are credited.Simon van Noort, Scott Richard Shaw, Robert S. Copeland

## References

[B1] Baltazar CR (1962) *Ettchellsiaphilippinensis* sp. nov. (Dinapsinae, Megalyridae, Hymenoptera). Philippine Journal of Science 90: 219–220.

[B2] Binoy C Shaw SR Kumar PG Santhosh S Nasser M (2020) First discovery of a long-tailed wasp from the Indian subcontinent (Hymenoptera: Megalyroidea: Megalyridae). International Journal of Tropical Insect Science 40(4): 751–758. 10.1007/s42690-020-00126-7

[B3] Born J Linder HP Desmet P (2007) The Greater Cape Floristic Region. Journal of Biogeography 34(1): 147–162. 10.1111/j.1365-2699.2006.01595.x

[B4] Boulinier T Nichols JD Sauer JR Hines JE Pollock KH (1998) Estimating species richness: The importance of heterogeneity in species detectability. Ecology 79(3): 1018–1028. 10.1890/0012-9658(1998)079[1018:ESRTIO]2.0.CO;2

[B5] Brues CT (1923) A fossil genus of Dinapsidae from Baltic amber (Hymenoptera). Psyche (Cambridge, Massachusetts) 30(1): 31–35. 10.1155/1923/23910

[B6] Brues CT (1933) The parasitic Hymenoptera of the Baltic amber. Part 1. Bernstein-Forchungen (Amber Studies), Heft 3. Walter de Gruyter and Co. Berlin.

[B7] Cameron P (1909) Description of a new genus and species of parasitic Hymenoptera, representing a new tribe, from Kuching, Borneo. Deutsche Entomologische Zeitschrift 208–209. 10.1002/mmnd.48019090204

[B8] Chen H Liuhe B Zhang X (2021) Two new species of the family Megalyridae (Hymenoptera) from China. ZooKeys 1043: 21–31. 10.3897/zookeys.1043.65223 34163293 PMC8211637

[B9] Deng J-Y van Noort S Compton SG Chen Y Greeff JM (2020) The genetic consequences of habitat specificity for fig trees in southern African fragmented forests. Acta Oecologica 102: e103506. 10.1016/j.actao.2019.103506

[B10] Eeley HAC Lawes MJ Piper SE (1999) The influence of climate change on the distribution of indigenous forest in KwaZulu-Natal, South Africa. Journal of Biogeography 26(3): 595–617. 10.1046/j.1365-2699.1999.00307.x

[B11] Fahringer J (1928) Die Megalyriden. Archiv für Naturgeschichte. Abteiling A 92: 98–123.

[B12] Froggatt WW (1906) Notes on the hymenopterous genus *Megalyra* Westwood, with descriptions of new species. Proceedings of the Linnean Society of New South Wales 31: 399–407.

[B13] Gauld ID (1988) Evolutionary patterns of host utilization by ichneumonoid parasitoids (Hymenoptera: Ichneumonidae and Braconidae). Biological Journal of the Linnean Society. Linnean Society of London 35(4): 351–377. 10.1111/j.1095-8312.1988.tb00476.x

[B14] Gauld ID (1991) The Ichneumonidae of Costa Rica, 1. Introduction, keys to subfamilies, and keys to the specie of the lower pimpliform subfamilies Rhyssinae, Pimplinae, Poemeniinae, Acaenitinae, and Cyllocerinae. Memoirs of the American Entomological Institute 47: 1–589.

[B15] Gauld ID Hanson PE (1995) The evaniomorph families, Chapter 8. In: Hanson PE, Gauld ID (Eds) The Hymenoptera of Costa Rica, Oxford University Press, Oxford, 185–208.

[B16] Gess FW (1964) The discovery of a parasite of the *Phoracantha* beetle (Coleoptera: Cerambycidae) in the Western Cape. Journal of the Entomological Society of Southern Africa 27: 152. https://journals.co.za/doi/10.10520/AJA00128789_3853

[B17] Gibson GAP (1985) Some pro-and mesothoracic structures important for phylogenetic analysis of Hymenoptera, with review of terms used for the structures. Canadian Entomologist 117(11): 1395–1443. 10.4039/Ent1171395-11

[B18] Hacker H (1913) Some notes on Queensland insects. Memoirs of the Queensland Museum 2: 96–100.

[B19] Hacker H (1915) Notes on the genus *Megachile* and some rare insects collected during 1913–1914. Memoirs of the Queensland Museum 3: 137–141.

[B20] Harper G Steininger M Tucker C Juhn D Hawkins F (2007) Fifty years of deforestation and forest fragmentation in Madagascar. Environmental Conservation 34(4): 325–333. 10.1017/S0376892907004262

[B21] Harris RA (1979) A glossary of surface sculpturing. Occasional papers in Entomology 28: 1–31.

[B22] Hedqvist KV (1959) Hymenoptera (Ichneumonoidea): Megalyridae. South African Animal Life 6: 485–490.

[B23] Hedqvist KV (1967) Notes on Megalyridae [Hym. Ichneumonoidea] and description of new species from Madagascar. Annales de la Société Entomologique de France 3: 239–246.

[B24] Huber JT Sharkey MJ (1993) Chapter 3, Structure. In: Goulet H, Huber J (Eds) Hymenoptera of the World: an identification guide to families, Research Branch, Agriculture Canada, Ottawa, 13–59.

[B25] Ivanova NV Dewaard JR Hebert PD (2006) An inexpensive, automation-friendly protocol for recovering high-quality DNA. Molecular Ecology Notes 6(4): 998–1002. 10.1111/j.1471-8286.2006.01428.x

[B26] Jussila R Kapyla M (1975) Observations on *Townesiatenuiventris* (Hlmgr.) (Hym., Ichneumonidae) and its hosts *Chelostomamaxillosum* (L.) (Hym., Megachilidae) and *Trypoxylonfigulus* (L.) (Hym., Sphecidae). Annales Entomologici Fennici 41: 81–86.

[B27] Kawada R Barbosa DN Azevedo CO (2014) *Cryptalyra* (Hymenoptera, Megalyridae) from Maranhão, Brazil: Three new species discovered after a large collecting effort. ZooKeys 442: 85–104. 10.3897/zookeys.442.8237 PMC4205498 25349491

[B28] Lawes MJ (1990) The distribution of the Samango monkey (*Cercopithecusmitiserythrarchus* Peters, 1852 and *Cercopithecusmitislabiatus* I. Geoffroy, 1843) and forest history in southern Africa. Journal of Biogeography 17(6): 669–680. 10.2307/2845148

[B29] Lawes MJ (2004) Conservation of fragmented populations of *Cercopithecusmitis* in South Africa: the role of reintroduction, corridors and metapopulation ecology. In: Glenn ME Cords M (Eds) The Guenons: diversity and adaptation in African monkeys.Springer, Boston, MA, 375–392. 10.1007/0-306-48417-X_24

[B30] Lawes MJ Eeley HAC Findlay NJ Forbes D (2007) Resilient forest faunal communities in South Africa: A legacy of palaeoclimatic change and extinction filtering? Journal of Biogeography 34(7): 1246–1264. 10.1111/j.1365-2699.2007.01696.x

[B31] Linder HP (2003) The radiation of the Cape flora. Biological Reviews of the Cambridge Philosophical Society 78(4): 597–638. 10.1017/S1464793103006171 14700393

[B32] Macdevette DR Macdevette K Gordon IG Bartholomew R (1989) The floristics of the Natal indigenous forests. In Gordon IG (Ed.) Natal indigenous forest: a preliminary collection of reports on indigenous forests, Natal Parks Board, 1–20.

[B33] Madl M (2010) A catalogue of the Megalyridae (Megalyroidea), Stephanidae (Stephanoidea) and Trigonalidae (Trigonaloidea) of the Malagasy Subregion (Hymenoptera). Entomofauna. Zeitschrift für Entomologie 31: 365–372.

[B34] Maley J (1996) The African rain forest - main characteristics of changes in vegetation and climate from the Upper Cretaceous to the Quaternary. Proceedings of the Royal Society of Edinburgh 104B: 31–73. 10.1017/S0269727000006114

[B35] Mason WRM (1993) Chapter 11, Superfamilies Evanioidea, Stephanoidea, Megalyroidea, and Trigonalyoidea. In: Goulet H Huber J (Eds) Hymenoptera of the World: an identification guide to families.Research Branch, Agriculture Canada, Ottawa, 510–520.

[B36] McConnell W Kull C (2014) Protecting lemurs: Madagascar’s forests. Science 344(6182): e358. 10.1126/science.344.6182.358-a 24763569

[B37] Mesaglio T Shaw SR (2022) Observations of oviposition behaviour in the long-tailed wasp *Megalyrafasciipennis* Westwood, 1832 (Hymenoptera: Megalyridae). Austral Ecology 47(4): 889–893. 10.1111/aec.13163

[B38] Mita T Shaw SR (2012) A taxonomic study on the genus *Ettchellsia* Cameron, with descriptions of three new species (Hymenoptera, Megalyridae, Dinapsini). ZooKeys 254: 99–108. 10.3897/zookeys.254.4182 PMC3561919 23378818

[B39] Mita T Shaw SR (2020) A taxonomic study of *Dinapsis* Waterson, 1922 from Madagascar (Hymenoptera: Megalyridae, Dinapsini): crested wasps of the *hirtipes* species-group. Zootaxa 4858(1): 71–84. 10.11646/zootaxa.4858.1.4 33056242

[B40] Mita T Konishi K Terayama M Yamane S (2007) Two new species of the genus *Carminator* Shaw from Japan, the northernmost record of extant Megalyridae (Hymenoptera). Entomological Science 10(2): 201–208. 10.1111/j.1479-8298.2007.00213.x

[B41] Morley RJ (2000) The African rain forest. In: Morley RJ (Ed.) Origin and evolution of tropical rainforests.John Wiley and Sons Ltd., New York, 131–161.

[B42] Mucina L Geldenhuys CJ (2006) Afrotemperate, subtropical and azonal forests. Strelitzia 19: 584–614.

[B43] Naumann ID (1983) The biology of mud nesting Hymenoptera (and their associates) and Isoptera in rock shelters of the Kakadu region, Northern Territory. In: Gillespie D et al. (Eds) The rock art sites of Kakadu National Park - some preliminary research findings for their conservation and management. Australian National Parks and Wildlife Service, Special Publication 10, ANPWS, Canberra, 129–189.

[B44] Naumann ID (1987) A new megalyrid (Hymenoptera: Megalyridae) parasitic on a sphecid wasp in Australia. Australian Journal of Zoology 26: 215–222. 10.1111/j.1440-6055.1987.tb00289.x

[B45] Penev L Sharkey M Erwin T van Noort S Buffington M Seltmann K Johnson N Taylor M Thompson F Dallwitz M (2009) Data publication and dissemination of interactive keys under the open access model. ZooKeys 21: 1–17. 10.3897/zookeys.21.274

[B46] Perrichot V (2009) Long-tailed wasps (Hymenoptera: Megalyridae) from Cretaceous and Paleogene European amber. Paleontological Contributions 1: 1–35. 10.17161/PC.1808.5468

[B47] Poinar Jr G Shaw SR (2007) *Megalyrabaltica* Poinar and Shaw n. sp. (Hymenoptera: Megalyridae), a long-tailed wasp from Baltic amber. Zootaxa 1478: 65–68.

[B48] Quicke DLJ (2015) The Braconid and Ichneumonid Parasitoid Wasps: Biology, Systematics, Evolution and Ecology. John Wiley & Sons, New York, 704 pp. 10.1002/9781118907085

[B49] Rasnitsyn AP (1975) Vysshie pereponchatokrylye mezosoya. Trudy Paleontologischeskogo Instituta Akedemii Nauk SSR 147: 1–132. [HymenopteraApocrita of the Mesozoic]

[B50] Rasnitsyn AP (1977) New Hymenoptera from the Jurassic and Cretaceous of Asia. Paleontologicheskii Zhurnal 1977: 98–108. [in Russian] [English translation in. Paleontological Journal 11: 349–357]

[B51] Ratnasingham S Hebert PDN (2007) Bold: The barcode of life data system (www.barcodinglife.org). Molecular Ecology Notes 7(3): 355–364. 10.1111/j.1471-8286.2007.01678.x 18784790 PMC1890991

[B52] Rodd NW (1951) Some observations on the biology of Stephanidae and Megalyridae (Hymenoptera). Australian Zoologist 11: 341–346.

[B53] Sharkey MJ Yu DS van Noort S Seltmann K Penev L (2009) Revision of the Oriental genera of Agathidinae (Hymenoptera, Braconidae) with an emphasis on Thailand including interactive keys to genera published in three different formats. ZooKeys 21: 19–54. 10.3897/zookeys.21.271

[B54] Shaw SR (1987) Three new megalyrids from South America (Hymenoptera: Megalyridae). Psyche 94(1–2): 189–199. 10.1155/1987/28207

[B55] Shaw SR (1988) *Carminator*, a new genus of Megalyridae (Hymenoptera) from the Oriental and Australian regions, with a commentary on the definition of the family. Systematic Entomology 13(1): 101–113. 10.1111/j.1365-3113.1988.tb00233.x

[B56] Shaw SR (1990a) A taxonomic revision of the long-tailed wasps of the genus *Megalyra* Westwood (Hymenoptera: Megalyridae). Invertebrate Taxonomy 3: 1000–1052. 10.1071/IT9891005

[B57] Shaw SR (1990b) Phylogeny and biogeography of the parasitoid wasp family Megalyridae (Hymenoptera). Journal of Biogeography 17(6): 569–581. 10.2307/2845141

[B58] Shaw SR (2003) A new *Cryptalyra* species from Colombia (Hymenoptera: Megalyridae). Zootaxa 248(1): 1–4. 10.11646/zootaxa.248.1.1

[B59] Shaw SR (2005) Megalyroidea, Megalyridae. Tree of Life web project page. [Available online:] http://tolweb.org/tree?group=Megalyridae&contgroup=Apocrita

[B60] Shaw SR van Noort S (2009) A new *Dinapsis* species from the Central African Republic (Hymenoptera: Megalyridae: Dinapsini). Zootaxa 2118(1): 30–36. 10.11646/zootaxa.2118.1.2

[B61] Shorthouse DP (2010) SimpleMappr, an online tool to produce publication-quality point maps. https://www.simplemappr.net [Accessed 2022/01/24]

[B62] van Noort S (2004) Ichneumonid (Hymenoptera: Ichneumonoidea) diversity across an elevational gradient on Monts Doudou in south-western Gabon. California Academy of Sciences Memoir 28: 187–216.

[B63] van Noort S (2022) Waspweb. https://www.waspweb.org [Accessed 2022/01/16]

[B64] van Noort S Picker M (2011) Wasps, Bees, Ants. Class Insecta, Order Hymenoptera. In: Picker M Griffiths C (Eds) Alien and Invasive Animals.A South African perspective. Struik Nature, 140–146.

[B65] van Noort S Shaw SR (2009) *Megalyridiacapensis* (Hymenoptera: Megalyridae: Megalyridiini) a relict species endemic to South Africa. African Natural History 5: 1–8.

[B66] van Noort S Belokobylskij SA Touret-Alby A (2021) Rediscovery of the endemic Afrotropical genus *Spathioplites* (Hymenoptera, Braconidae, Doryctinae) with major range extension records for *Spathioplitesphreneticus*. African Invertebrates 62(2): 497–520. 10.3897/afrinvertebr.62.74103

[B67] Vilhelmsen L Perrichot V Shaw SR (2010) Past and present diversity and distribution in the parasitic wasp family Megalyridae (Hymenoptera). Systematic Entomology 35(4): 658–677. 10.1111/j.1365-3113.2010.00537.x

[B68] von Maltitz G Mucina L Geldenhuys C Lawes M Eeley H Adie H Bailey C (2003) Classification system for South African indigenous forests: an objective classification for the Department of Water Affairs and Forestry. Environmentek Report ENV-P-C, 2003–017, 1–284.

[B69] Waterston J (1922) A new family of Hymenoptera from South Africa. Annals & Magazine of Natural History 10(9): 418–420. 10.1080/00222932208632792

[B70] Westwood JO (1832) In: Griffith E. Supplement on the Hymenoptera. In: Cuvier G (Ed.) Animal Kingdom, Class Insecta.Whittaker, Treacher and Co., London, 389–576.

[B71] White F (1981) The history of the Afromontane archipelago and the scientific need for its conservation. African Journal of Ecology 19(1–2): 33–54. 10.1111/j.1365-2028.1981.tb00651.x

[B72] White F (1983) Vegetation of Africa: a descriptive memoir to accompany the UNESCO/AETFAT/UNSO vegetation map of Africa. UNESCO, Paris.

